# A Nomothetic Span Approach to the Construct Validation of Sustained Attention Consistency: Re-Analyzing Two Latent-Variable Studies of Performance Variability and Mind-Wandering Self-Reports

**DOI:** 10.1007/s00426-023-01820-0

**Published:** 2023-06-14

**Authors:** Matthew S. Welhaf, Michael J. Kane

**Affiliations:** 1 Department of Psychological and Brain Sciences, Washington University in St. Louis, CB 1125 One Brookings Drive, St. Louis, MO 63130-4899 USA; 2 Department of Psychology, University of North Carolina at Greensboro, P.O. Box 26170, Greensboro, NC 27402-6170 USA

## Abstract

The ability to sustain attention consistency is frequently assessed using either objective behavioral measures, such as reaction time (RT) variability, or subjective self-report measures, such as rates of task-unrelated thought (TUT). The current studies examined whether the *individual-difference covariation* in these measures provides a more construct valid assessment of attention consistency than does either alone. We argue that performance and self-report measures mutually validate each other; each measurement approach has its own sources of error, so their shared variance should best reflect the attention consistency construct. We reanalyzed two latent-variable studies where RT variability and TUTs were measured in multiple tasks (Kane et al. in J Exp Psychol Gen 145:1017–1048, 2016; Unsworth et al. in J Exp Psychol Gen 150:1303–1331, 2021), along with several nomological network constructs to test the convergent and discriminant validity of a general attention consistency factor. Confirmatory factor analyses assessing bifactor (preregistered) and hierarchical (non-preregistered) models suggested that attention consistency can be modeled as the shared variance among objective and subjective measures. This attention consistency factor was related to working memory capacity, attention (interference) control, processing speed, state motivation and alertness, and self-reported cognitive failures and positive schizotypy. Although bifactor models of general attention consistency provide the most compelling construct validity evidence for a specific ability to sustain attention, multiverse analyses of outlier decisions suggested they are less robust than hierarchical models. The results provide evidence for the general ability to sustain attention consistency and suggestions for improving its measurement.

**Supplementary Information:**

The online version contains supplementary material available at 10.1007/s00426-023-01820-0.

## Introduction

People sometimes strive to keep their attention directed on their current task and goals but do so with varying success. We may neglect to attach a file to an e-mail message, forget to stop at the grocery store on the way home from work, or even fail to check our surroundings before driving our car in reverse. Everyday observations suggest that some people better and more consistently sustain their attention than do others, showing more stable performance with fewer behavioral lapses, and less frequently experiencing distracted or off-task thoughts. What might account for these individual differences?

Despite attentional lapses being responsible for real-world errors, the ability to sustain attention consistency has been less thoroughly studied by psychologists than have other components of attention, such as selective, divided, and switching attention (Esterman & Rothlein, 2019). And, despite sustained attention supporting the regulation and control of other cognitive processes and behavior, it has been understudied relative to the executive functions of inhibition, updating, and switching (Miyake & Friedman, 2012). Research has nonetheless identified distinct, yet correlated, empirical measures that reflect attentional instability—variability in task performance and self-reports of mind-wandering—but it has not yet considered that the overlap in these measures might be the most valid reflection of sustained attention (in)ability. The goal of the current study is to evaluate the construct validity of attention consistency measurement from a nomothetic span, or individual differences, perspective. We investigate whether there are stable individual differences in failures of attention consistency, as indicated by performance and self-report measures, and if so, ask what other psychological or contextual factors might predict them.

The broad construct of sustained attention is not new, of course, and several theoretical models have addressed how and why attention fluctuates (for reviews see Esterman & Rothlein, 2019; Fortenbaugh et al., 2017). Traditionally, sustained attention has been viewed—and studied empirically—as the ability to maintain performance over many task trials (and many minutes). Failures of sustained attention from this view correspond to the so-called “vigilance decrement” (e.g., Lim & Dinges, 2008; Mackworth, 1950; Parasuraman, 1986). Here, performance—be it reaction time or accuracy, usually in detecting rare target signals—worsens as time on task increases.

We are primarily concerned, instead, with the moment-to-moment stability of attention, or “attentional consistency” (Unsworth & Miller, 2021), which may be a (partially) distinct form of sustained attention from that reflected in the general worsening of performance over time (Thomson et al., 2015). Specifically, we define sustained attention consistency as *the purposeful act of maintaining optimal task focus to successfully, and steadily, perform goal-relevant actions*; we will primarily use the terms “attention consistency”, “attention inconsistency,” and “attention fluctuation” from here forward, but when we do occasionally use “sustained attention,” “sustained attention lapses,” or “sustained attention consistency,” we will also be referring to this momentary (in)stability of focus. Note that our definition of attention consistency shares conceptual overlap with other views of executive attention abilities like goal maintenance (e.g., Engle & Kane, 2004) and proactive control (e.g., Braver et al., 2007). Goal maintenance and proactive control are typically assessed using tasks that require resolving some form of response or cue conflict for successful performance (e.g., antisaccade, Stroop, or AX-CPT). Attention consistency may often be necessary, however, even in tasks that don’t have a strong conflict component but that challenge one to perform simple operations consistently over time (e.g., the psychomotor vigilance task or simple choice RT tasks).

## Sustained Attention Consistency as the Covariation of Objective and Subjective Measures

The cognitive psychology literature has taken two approaches to measuring attention consistency. In the following sections, we describe both, which we refer to as *objective* (based on performance data) and *subjective* (based on self-report data). Each section describes how these measures reflect attention (in)stability and their limitations when used in isolation. We will argue that a combination of objective and subjective indicators—and specifically their shared variance—will provide the most valid assessment of the ability to sustain attention consistency and its individual-differences variation.

We build this argument on the precedent that in some traditional sustained attention tasks, different performance indicators seem to reflect different types or degrees of sustained attention failure (Cheyne et al., 2009; Unsworth et al., 2021). For example, Cheyne et al. (2009) found that three performance measures all predicted unique variance in no-go accuracy in a go/no-go task. Each of these measures also mapped onto distinct, hypothetical attentional states with increasing levels of disengagement. RT variability reflects State 1 (*focal inattention)*, characterized by brief periods of attentional instability, which produces near misses and variable performance; anticipation errors reflect State 2 (*global inattention)*, where top-down attention is disengaged from the current task to the point where automatic, “mindless” behaviors take over. Finally, omission errors reflect State 3 (*behavioral/response disengagement)*, where subjects’ attention is withdrawn from the task to the point where they fail to engage in any task-appropriate responding. Although more theoretical and empirical work needs to be done to convincingly establish an inattention or disengagement continuum (Tay & Jebb, 2018), modeling the overlap of various measures (and different methodological approaches) may be a more construct valid way of assessing the ability to sustain momentary attention than relying solely on any one type of error-prone measure.

### Objective (Performance-Based) Measures of Attention Consistency

#### Reaction Time Variability

Optimal attention performance can be measured as the magnitude of a subject’s RT variability across a task, or as the rate or durations of a subject’s relatively long RTs within a task (Bunce et al., 2004; West et al., 2002). That is, if a subject is effectively sustaining focused attention across a task that makes consistent cognitive demands across trials, then their RTs should be similar from trial to trial. RT variability and extremely long RTs reflect how *consistently* (or *inconsistently*) a subject performs a repetitive task.

Early work by Bills (1931, 1935) showed that after extended periods of continuous work on a task, subjects occasionally showed very long RTs (e.g., twice the mean; “blocks”), which were often followed by more variable or erroneous performance (see also Bertelson & Joffe, 1963; Fiske & Rice, 1955; Sanders & Hoogenbroom, 1970). In modern tasks, like the psychomotor vigilance task, subjects must maintain focus for some variable and unpredictable duration (typically 1–10 s) before the stimulus numbers begin counting upwards, which is the signal for subjects to hit a key to stop the clock. Here, the number of “lapses” (i.e., RTs > 500 ms) is frequently used as a dependent measure that represents sustained attention failure (e.g., Lim & Dinges, 2008; Unsworth & Robison, 2016). Like blocks, the number of lapses reflects variation in attention consistency because they seem to capture instances where subjects are not optimally task-focused (i.e., they’re not optimally ready to respond to the target digits beginning to count upward).

Attention consistency is also assessed via trial-to-trial RT variability across a task. Here, subjects with better attention consistency should show less variability, with few very short or long RTs. Common approaches to measuring RT variability include intra-individual standard deviation (RTsd), coefficient of variation (CoV), or Rhythmic Response Times (RRTs). RTsd takes the SD of RTs across correct trials for each subject, whereas CoV expresses RTsd as a function of subjects’ *M* RT (CoV = [SD/*M*] × 100). RRTs reflect the time between response and stimulus onsets (Laflamme et al., 2018; Seli et al., 2013a, 2013b), so they can be positive (responding after the stimulus) or negative (responding before the stimulus); RRTs are often taken across a number of trials (e.g., 5) to create a moving window of variability calculated across the task.^1^

Finally, momentary fluctuations in sustained attention can be assessed by fitting a subject’s RTs to a distributional model. The ex-Gaussian model, for example, a convolution of a Gaussian (normal) and an exponential distribution, provides three parameters: *μ* and *σ* represent the mean and standard deviation of the Gaussian component, respectively, while *τ* represents the *M* and SD of the exponential component (i.e., the tail). In general, *τ* reflects longer-than-average RTs and may capture attention (in)consistency to some degree (although ex-Gaussian parameters are not purely mapped onto any one or several psychological processes; Matzke & Wagenmakers, 2009).

#### Performance Accuracy

Accuracy-based measures may also reflect momentary lapses of sustained attention, at least in part. In the Sustained Attention to Response Task, errors of omission (i.e., not responding to a “go” trial) and errors of commission (i.e., erroneously responding to a “no-go” trial) might reflect even greater task disengagement than is captured by variable response speed (Cheyne et al., 2009; Unsworth et al., 2021). That is, errors of omission might reflect a complete disengagement from the task whereas errors of commission might reflect being captured enough by monotonous responding that individuals keep making repetitive responses when they are not supposed to. Likewise, during continuous tracking tasks, in which subjects attempt to closely follow an object onscreen with a stylus or cursor, subjects may occasionally exhibit “flat spots,” or brief instances where they fail to respond to the stimuli (Peiris et al., 2006; Unsworth et al., 2021).

#### Limitations of Objective Indicators of Attention Consistency

Like other cognitive ability measures, objective indicators of attention consistency are not process-pure. Longer-than-normal RTs can certainly be caused by attention lapses. But subjects can also experience long RTs simply because they are generally slower than other subjects, or because they momentarily changed their response strategy (e.g., speed-accuracy trade-offs or post-error slowing). Long RTs can also result from involuntary actions (e.g., sneezing or yawning) or cases where subjects take intentional “rest breaks” during a trial. Further, task-specific processes, unrelated to attentional consistency, may affect performance variability, especially if a task presents trial types with differing cognitive demands (e.g., Stroop tasks or Sternberg item-recognition tasks). Table 1 describes the sustained attention demands, as well as the non-sustained attention sources of measurement error, for commonly studied sustained attention tasks. T﻿hus, when assessing attention consistency using solely objective indicators, any one performance measure won’t fully capture all types or all instances of disengagement (and it will capture extraneous sources of measurement error). The performance variance that is common, or shared, across several of these objective measures should better reflect abilities to sustain attention consistency. Moreover, combining additional, non-performance indicators with performance assessments may provide for still more valid measurement of attention consistency, as we discuss below.

**Table 1:** Descriptions of commonly used attention consistency tasks

Task/citation	Description	How is sustaining attention consistency necessary?	Non-sustained attention influences
Psychomotor Vigilance Task (PVT; Lim & Dinges, 2008)	Simple RT task that presents subjects with a set of 0 s on-screen (like a stopwatch: “00.000”) and requires them to respond as quickly as possible when they notice that the numbers begin counting up after a variable delay	Necessary to maintain task focus/engagement and intrinsic alertness during unpredictable periods between the start of the trial and stimulus onset. Failing to sustain attention would result in longer than normal RT	Processing speed; SOA guessing strategy; impulsivity
Sustained Attention to Response Task (SART; Robertson et al., 1997)	A go/no-go task that requires subjects to respond to frequently presented items from one category (~ 89% of the trials) and withhold responses to rare targets (~ 11% of the trials)	High “go” trial frequency can lead to mindless, habitual, responding. Sustained attention is needed to overcome the mindless, and potentially erratic, responding and maintain consistency. Rare “no-go” trials also require sustained attention in order to prevent commission errors that might occur because of habitual responding	Response inhibition; response strategies (i.e., speed-accuracy tradeoff); processing speed; impulsivity; knowledge of stimuli used in task (i.e., knowledge of different animals and fruits)
Metronome Response Task (MRT; Laflamme et al., 2018; Seli et al., 2013a, 2013b)	A continuous performance task, in which visual or auditory stimuli are presented at a constant rate, that requires subjects to respond in synchrony with the presentation of the stimuli	Repetitive presentation of low arousing stimuli for extended durations can elicit inconsistent responding. Sustained attention is needed to maintain consistent responding and not mis-time responses to stimuli	Familiarity and skill with rhythm or music; time estimation; counting strategies
Continuous Temporal Expectancy Task (CTET; O’Connell et al., 2009)	Subjects view a series of abstract images that are perceptually similar to each other; their goal is to respond to rare target stimuli that are presented for longer-than-usual durations (1000–1200 ms) among frequent non-targets that are presented briefly (600–800 ms). Attention-capturing stimulus onsets/offsets are non-diagnostic to target detection	Sustained attention is needed to focus and notice small temporal discrepancies among perceptually similar and repetitive stimuli	Visual and temporal discrimination ability; response criterion setting/strategy
Gradual Onset Continuous Performance Task (gradCPT; Rosenberg et al., 2013)	A go/no-go continuous performance task that presents subjects with frequent non-target stimuli and infrequent targets (similar to the SART). However, in the gradCPT, the stimuli gradually fade into one another, eliminating stimulus onsets/offsets which can capture attention	High “go” trial frequency can lead to mindless, habitual, responding. Sustained attention is needed to overcome the mindless, and potentially erratic, responding and maintain consistency. Rare “no-go” trials also require sustained attention in order to prevent commission errors that might occur because of habitual responding	Visual discrimination ability; response inhibition; processing speed; impulsivity; response strategies (i.e., speed-accuracy tradeoff; response criterion setting)

### Subjective (Self-Report-Based) Indicators of Attention Consistency

Objective indicators, like RTsd, may capture relatively subtle fluctuations in attention. However, some attention-consistency failures may be more obvious, and perhaps conspicuous enough to be easily reported by subjects when asked. Self-report measures of attention consistency aim to capture off-task thought experiences that are characteristic of everyday attention failures.

One commonly used, subjective approach to assessing attention consistency, both in the lab and in everyday life, is the thought-probe method. This technique is most frequently used to capture subjects’ mind-wandering (or task-unrelated thought; TUT) experiences as they occur, and has been used in a variety of tasks and contexts, including attention tasks (e.g., Hutchison et al., 2020; Kane et al., 2016; McVay & Kane, 2012a), reading tasks (e.g., Franklin et al., 2014; McVay & Kane, 2012b; Unsworth & McMillan, 2014), live classroom or virtual learning environments (e.g., Hollis & Was, 2016; Kane et al., 2021a; Wammes et al., 2016), and in everyday life (e.g., Kane et al., 2007, 2017a; Killingsworth & Gilbert, 2010; Marcusson-Clavertz et al., 2016). Here, subjects are repeatedly and unpredictably interrupted during a task or activity and asked to report on the contents of their thoughts in the moment immediately preceding the probe appearance. Subjects typically indicate whether they were focused on the task or experiencing TUTs.

#### Thought-probe Methods and Measures

Various aspects of mind-wandering have been investigated using thought-probe methods (see Seli et al., 2018; Weinstein, 2018). In some studies, subjects answer a simple “yes/no” question about whether they were focused on the task (e.g., Franklin et al., 2014; Song & Wang, 2012; Szpunar et al., 2013). Other studies present thought-choice menus that allow subjects to select among categories or qualities of thoughts, such as thought content (e.g., worries, fantastical daydreams), temporal orientation (e.g., past events, future goals), emotional valence (e.g., positive, negative), or intentionality (e.g., deliberate, spontaneous; Banks et al., 2016; Smallwood et al., 2009; Stawarczyk et al., 2011, 2013; Unsworth & McMillan, 2014). Still others have used Likert scales to rate depth or intensity of mind-wandering (e.g., Allen et al., 2013; Christoff et al., 2009; Kane et al., 2021b). Thus, much like there are different tasks in which performance variability is measured as objective indicators of attention consistency, there are a variety of ways to subjectively assess momentary attention fluctuations or failures that are experienced as mind-wandering.

The typical measure derived from thought probes is TUT rate (number of TUT reports/number of probes) which estimates the frequency with which subjects are not focusing on their task. Subjects report being off-task 30–60% of the time, on average, suggesting that TUTs occur frequently across contexts. At the same time, individual differences around those averages are substantial. TUT reports are associated with poorer task performance in the moment, and people who report more TUTs tend to perform their tasks more poorly, further validating that TUTs reflect momentary failures of attention consistency.

#### Limitations of Subjective Indicators of Attention Consistency

Like performance measures of attention consistency, TUT reports come with confounds and concerns to consider (Kane et al., 2021b; Weinstein, 2018). Most obviously, as these self-reports rely on introspection, we must consider the potential influence of reporting biases and errors (Hurlburt & Heavey, 2001; Nisbett & Wilson, 1977).

Thought reports to probes might be impacted by the frequency with which probes occur in the task. Too frequent probing might provide reminders to stay on task or not give enough time for subjects’ minds to wander between probes, whereas too infrequent probing may miss instances of mind-wandering that occur between probes (Welhaf et al., 2023). Only a few studies have explicitly examined this possibility and the findings are mixed. Robison et al. (2019) found that more frequent probing (13 vs. 7% of total trials) did not influence TUT rates. However, studies by both Seli et al., (2013a) and Schubert et al. (2019) found that more frequent probing (across ranges of 1–6% of trials) resulted in lower TUT rates, suggesting that frequent probes act as on-task reminders or thought-flow disruptors.

An additional concern about probing during a task is that responses to probes might be biased by reactivity to performance. That is, when subjects make an error and a thought probe follows that error, subjects may use their performance as evidence for where their thoughts were focused. Few studies have examined this possibility (Head & Helton, 2018; Kane et al., 2021b; Schubert et al., 2019), but they suggest some reactivity in tasks that elicit salient errors (e.g., go/no-go tasks). Schubert et al. (2019), for example, found that TUT reports in a SART were more frequent following “no-go” compared to “go” trials and that TUT reports were more frequent following “no-go” errors compared to correct “no-go” trials.

Although thought probes vary across studies, recent work suggests that some findings are robust across different thought-probe variations. For example, Kane et al. (2021b) found similarities in *M* TUT rate, TUT rate reliability across tasks, within-person associations between TUTs and go/no-go performance, and between-person TUT rate associations with theoretically relevant constructs (e.g., executive-control ability) across four different probe types. These findings provide generally supportive evidence for acceptable construct validity of the thought-probe method.

Kane et al. (2021b) also noted some concerns, however, about specific probe types (i.e., asking about intentionality or depth of mind-wandering). One common finding is that “no-go” accuracy in the sustained attention to response task (SART) is worse on trials before TUT reports compared to on-task reports. Kane et al. (2021b) replicated this finding but found it was more pronounced for probes asking about the intentionality or depth of mind-wandering (versus its content), suggesting that these TUT reports might be especially influenced by reactivity to performance. Thus, it’s possible that not all TUT reports equally reflect attention consistency failures or are equally affected by sources of measurement error.

Just as the field should not rely solely on any one objective measure of attention consistency in any one task, it also should not rely solely on any one self-report measure. Rather, the variance that is common across subjective indicators from multiple contexts and tasks (and perhaps across different types of thought probes) should yield a more accurate attention consistency measure. And, further, as argued previously, variance that is common across multiple subjective indicators and multiple objective indicators should provide an optimally construct valid assessment of general ability to sustain attention consistency.

### Correlations between Objective and Subjective Measures

Objective and subjective indicators provide starkly different approaches to measuring attention abilities. If they are, nonetheless, both influenced by a general ability to sustain attention consistency, then then they should be correlated. Indeed, at the between-person level of analysis, latent variable correlations between RT variability and TUT rate factors typically range from 0.30–0.50 (Kane et al., 2016; Unsworth, 2015; Unsworth et al., 2021; Welhaf et al., 2020; for similar RT variability–TUT correlations in single experimental tasks, see Löffler et al., 2021; Stawarczyk et al., 2014; Yamashita et al., 2021). These factors are thus only *moderately* correlated: subjects who report more off-task thoughts also show more inconsistent responding in simple attention and RT tasks, but the association between these measures is not strong. Despite this moderate correlation, the shared variance between subjective and objective measures of attention consistency should provide its most construct valid measurement.

Indeed, we argue that *because* of this moderate correlation, using the shared objective–subjective variance to measure attention consistency is especially important. These factors are not redundant and cannot be used interchangeably. Performance and self-report measures may not only capture different dimensions or depths of attention (in)consistency (e.g., Cheyne et al., 2009), but these approaches are also subject to different confounds, which uniquely influence their measurement. Relying on only one type of indicator as *the* measure of attention consistency in a study may lead to incorrect conclusions about how other theoretically relevant constructs correlate with the ability to sustain momentary attention. Instead, using what is common between these measures should be a more construct valid way to measure attention consistency than using either in isolation: researchers should assess the covariation between performance and self-report measures of attention consistency not *despite* their moderate correlation, but *because of* it.

At the within-person level of analysis, one would also expect poorer performance (i.e., more errors) and greater RT variability in the moments preceding TUT reports compared to on-task reports. Indeed, commission errors on the SART, where subjects erroneously press a key on “no-go” trials, are more likely to occur prior to TUTs than to on-task reports (e.g., Kane et al., 2021b; McVay & Kane, 2009, 2012a; Smallwood & Schooler, 2006; Stawarczyk et al., 2011). These findings are potentially supportive of construct validity, but also ambiguous, because TUT reports that follow errors might be reactively biased by subjects’ knowledge of their performance, as noted earlier (Schubert et al., 2019). Because subjects are likely less aware of their RT variability on the trials leading up to thought probes, however, examining RTs that precede thought reports should provide a less biased assessment of behavioral correlates of TUT experiences. In fact, RTs preceding TUTs are more variable than those preceding on-task reports (Bastian & Sackur, 2013; Kane et al., 2021b; Seli et al., 2013b).

### Summary of Attention Consistency Measurement

Objective and subjective measures of attention consistency frequently correlate with each other at between- and within-subject levels, suggesting they may both be impacted by a common underlying ability. At the same time, these correlations are of only moderate strength because each measurement type may capture different degrees of attention failure, and each has independent limitations and sources of error. We therefore argue that the optimal way of capturing people’s general ability to sustain attention consistency is to quantify the individual-differences variance that is common to both objective and subjective indicators.

## Evidence for the Construct Validity of Attention Consistency Measures

Considerable research has examined associations that attention-consistency indicators have with other theoretically relevant variables, taking a “nomothetic span” approach to construct validation (Cronbach & Meehl, 1955; Embretson, 1983). Studying these theoretically relevant variables, as part of the nomological network, provides evidence regarding convergent and discriminant validity.

### Correlations with Executive Attention Ability

Executive Attention theory (e.g., Burgoyne & Engle, 2020; Engle & Kane, 2004) argues that working memory capacity (WMC) broadly predicts performance on higher-order tasks (e.g., language comprehension, reasoning) because it reflects, in part, how effectively people can maintain ready access to goal-relevant information in the face of distraction or interference. According to this view, lower-WMC subjects have poorer goal maintenance ability, and so they should show more frequent attention lapses compared to higher-WMC subjects. Indeed, WMC measures (and related attention control measures, such as Stroop and antisaccade performance) correlate moderately with objective attention consistency measures, like RT variability, across a variety of tasks and measurement approaches (Kane et al., 2016; McVay & Kane, 2009, 2012a; Schmiedek et al., 2007; Schweizer & Moosbrugger, 2004; Unsworth, 2015; Unsworth et al., 2010, 2012a, 2012b, 2012c, 2021). Higher-WMC subjects are less variable in performance than are lower-WMC subjects.

WMC and attention control abilities are also frequently negatively associated with TUT rates in lab tasks (e.g., McVay & Kane, 2012b; Meier, 2019; Rummel & Boywitt, 2014; Unsworth & McMillan, 2017; Unsworth et al., 2012a, 2012b, 2012c, 2021) and in certain everyday-life contexts (Kane et al., 2007, 2017a, 2017b). In latent-variable studies that use multiple tasks to test construct-level correlations, the association between TUT rate and WMC often yields *r* =|0.20–0.30|, whereas associations between TUT rate and attention control performance is stronger, *r* =|0.35–0.45|. WMC and attention control abilities reliably predict attention consistency, whether derived from objective or subjective measures. At the same time, WMC and attention control ability appear to predict these different indicators of attention consistency to differing degrees. Thus, the field must examine how these constructs correlate with the shared variance between objective and subjective indicators to better understand their relationships with attention consistency.

### Correlations with Processing Speed

An important consideration with any RT measure, including RT variability, is that it may capture partly, or primarily, individual differences in general processing speed. Regarding attention consistency measurement, individuals may be more prone to extremely long or variable RTs because they have an overall slower processing rate. RT variability and speed can be highly collinear across experimental conditions and tasks (*r* ~ 0.90; Jensen, 1987, 1992; Wagenmakers & Brown, 2007). As well, both mean RT and RT variability are influenced by long RTs that might reflect attentional lapses. Thus, it is possible that the *apparent* inability to sustain attention consistency might simply be due to poor processing speed.

Measures of processing speed and objective indicators of attention consistency correlate substantially. For example, Unsworth et al. (2021) operationalized processing speed as subjects’ fastest 20% of trials within three attention tasks. A latent variable of objective attention consistency indicators correlated with the speed latent variable (*r* = 0.47): subjects with slower processing also exhibited poorer sustained attention. However, speed was also highly correlated with other cognitive ability measures like attention control, so structural equation models tested whether speed predicted any unique variance in objective attention consistency measures. It did not: after accounting for shared variance with other measures (like attention control), processing speed did not significantly predict the objective attention consistency latent variable. Attention control (but not WMC) predicted unique variance in objective attention consistency after accounting for shared variance among all the predictor constructs, suggesting that attention control, and not speed of processing or WMC, might be critical in explaining variation in attention lapses.

Additionally, latent-variable studies provide mixed evidence regarding correlations between processing speed and self-report indicators of attention consistency. Unsworth et al. (2021) found that processing speed measures were weakly associated with TUT rates (*r* = 0.24), whereas Welhaf et al. (2020) did not find a significant association between subjects’ shortest RTs and TUT rates (*r* = 0.09). Given that processing speed is more strongly related to objective than subjective indicators, measuring attention consistency as a latent variable reflecting the covariation between objective and subjective measures should best distinguish ability to sustain momentary attention from processing speed.

### Correlations with Cognitive Self-Report Variables

People who are more prone to cognitive failures in daily life should also show poorer attention consistency in lab tasks. Indeed, scores on retrospective self-report measures of everyday attention failures, like the Cognitive Failures Questionnaire (CFQ; Broadbent et al., 1982) and Attention-Related Cognitive Errors Scale (ARCES; Cheyne et al., 2006), correlate with both performance measures of attention consistency (e.g., Cheyne et al., 2006; McVay & Kane, 2009; Smilek et al., 2010; Steinborn et al., 2016) and TUT rates (e.g., McVay & Kane, 2009, 2013; Smallwood et al., 2004; Unsworth et al., 2021). In general, such cognitive failures may be slightly more strongly correlated with subjective (*r*s ~ 0.20) than with objective measures (*r*s ~ 0.15). Thus, measuring attention consistency as the individual-differences overlap in objective and subjective measures should provide a better estimate of its correlation with everyday cognitive failures.

### Correlations with Contextual-State Variables

People who are more motivated or interested in a task should exhibit better attention consistency in that task; being more willing to expend effort to focus on the task, or finding it rewarding to do so, should allow them to perform more optimally. Indeed, objective measures of attention consistency correlate with post-task self-report measures of motivation, interest, and arousal, with greater RT variability associated with lower state reports (*r* =  − 0.65 to − 0.30; Robison & Unsworth, 2018; Seli et al., 2015; Unsworth et al., 2021). As well, subjects who are more motivated, interested, or aroused report fewer TUTs across a variety of tasks and activities (*r* =  − 0.60 to − 0.27; Brosowsky et al., 2020; Hollis & Was, 2016; Kane et al., 2021a; Robison & Unsworth, 2015, 2018; Unsworth & McMillan, 2013, Unsworth et al, 2021; but see Rummel et al., 2021). While the ranges of these contextual variable correlations are quite similar, correlations with TUTs are often stronger than those with objective measures, perhaps due to similar self-report biases at play. Thus, by measuring attention consistency as the overlap in objective and subjective measures, we should better assess its relation to these contextual factors.

### Correlations with Personality Traits

Individual differences in certain personality traits may affect or reflect the ability to sustain attention consistency. People who experience high levels of emotion dysregulation (i.e., neuroticism), for example, may show greater inconsistency of attention due to ruminative tendencies or intrusive worries. People who are more willing to work toward goals or follow task instructions (i.e., high in conscientiousness or agreeableness), in contrast, may exhibit more consistent attention, perhaps especially in mundane tasks.

In terms of objective indicators of attention consistency, people high in neuroticism tend to show more variable RTs and more frequent behavioral lapses in simple tasks (Klein & Robinson, 2019; Robinson & Tamir, 2005; Unsworth et al., 2021). In terms of subjective indicators, correlations are less consistent. Students high in neuroticism frequently report more TUTs in the lab (Jackson et al., 2013; Kane et al., 2017a, 2017b; Robison et al., 2017; Unsworth et al., 2021), whereas students who are more goal-oriented (i.e., high in conscientiousness) report fewer TUTs in some studies (Jackson & Balota, 2012; Robison et al., 2020; Unsworth et al., 2021), but not in others (Jackson et al., 2013; Kane et al., 2017a, 2017b). Likewise, students who are more likely to comply with task instructions (i.e., high in agreeableness) reported fewer TUTs in one study (Unsworth et al., 2021), but not in another (Kane et al., 2017a, 2017b). Finally, openness to experience often fails to predict TUT rates in the lab (Smeekens & Kane, 2016; Unsworth et al., 2021), but does predict TUTs in daily life (Kane et al., 2017a, 2017b). Thus, neuroticism, which is unique in robustly correlating with both objective and subjective measures (in the lab, at least), might be related to a general ability to sustain attention consistency.

### Nomothetic Span Summary

Correlational studies provide evidence of convergent validity of attention consistency measures. Constructs that should predict it tend to do so: people with (a) better cognitive abilities, such as WMC, (b) higher motivation and interest in performing well, and (c) lower dispositional tendencies to experience sustained attention failures, all show less variable responding and lower TUT rates in simple tasks.

Although it is—and should be—rare to find constructs with *no* association (i.e., a null correlation) with attention consistency, given how fundamental sustained attention should be to so many domains of performance and experience, *relative* differences in correlation magnitudes can provide evidence for discriminant validity. First, attention control ability (typically measured with response-competition or interference-control tasks) frequently correlates more strongly with attention consistency measures (RT variability and TUT rate) than does WMC; one possible explanation for this difference is that WMC tasks are influenced by processes like memory storage or strategy choices that are less relevant to attention regulation. Second, RT variability indicators do not share unique variance with processing speed after accounting for other cognitive abilities, and TUT rates correlate weakly (if at all) with processing speed, suggesting that attention consistency is not simply a speed factor. Third, and lastly, some personality traits (e.g., conscientiousness, agreeableness) are not correlated with objective attention consistency measures, but are weakly and inconsistently correlated with subjective measures, suggesting they may not be related to a general ability to sustain momentary attention. Neuroticism and self-reported cognitive failures, however, correlate with both types of attention consistency measures.

As previously argued, the modest correlations between objective and subjective indications of attention consistency indicates a need to use their covariation as a more construct valid approach to assessing attention consistency than either measure on their own. Our perspective follows from how very different behavioral performance measures and self-report measures are, with each possibly reflecting different degrees of attentional disengagement (à la Cheyne et al., 2009) and each affected by unique sources of measurement error, both of which drive down their correlation.

A competing argument, however, is that objective and subjective indicators do not correlate strongly enough to indicate convergent validity and so they must instead reflect two *different* constructs (i.e., they provide discriminant validity evidence for one another). This argument implies that only one of these indicator types can be a construct valid measure of attention consistency. We raise several objections to this. First, objective and subjective indicators of attention consistency regularly correlate with each other at both within- and between-subject levels, suggesting that both reflect, at least partially, a failure to sustain momentary attention. Further, each type of indicator correlates with other nomological network constructs in theory-consistent ways. For example, people with higher WMC and attention control abilities show better scores on objective and subjective attention consistency measures. Likewise, certain dispositional characteristics (e.g., agreeableness) show reliable null associations with *both* indicator types, suggesting that constructs that should not correlate with attention consistency do not, regardless of the indicator used. Proposing that these two types of indicators reflect two different constructs implies that one of these two literatures is simply wrong about the body of relevant evidence and the claims that their measures (i.e., objective performance measures or subjective self-reports) reflect the ability to sustain momentary attention.

## Goals of the Current Studies

Many studies have investigated the nomological network of attention consistency, or how objective and subjective measures correlate with each other or with theoretically relevant variables. If these two forms of measurement are both presumed to reflect variation in attention consistency, albeit imperfectly, then their covariation should best reflect the general ability to sustain momentary attention: each indicator type may reflect different degrees of attention failure and each has its own source of measurement error that may impact attention consistency measurement if used on its own, but what they measure in common should reflect the attention consistency construct especially well.

The present studies’ goals were (1) to assess whether a general attention consistency construct exists that reflects the individual-differences overlap in objective and subjective measures and, if so, (2) to examine how theoretically relevant constructs like cognitive ability (e.g., WMC, attention control, and processing speed), contextual-state variables (e.g., task-specific motivation and alertness), and dispositional characteristics (e.g., everyday cognitive failures and personality traits) correlate with this common attention consistency factor. We reanalyzed data from two large latent-variable studies that had (a) multiple tasks with objective performance measures of attention consistency and (b) probed self-report assessments of TUTs within multiple tasks (Kane et al., 2016; Unsworth et al., 2021). These datasets allowed us to use confirmatory models to test whether there was enough variance shared between the objective and subjective measures to model a general factor of attention consistency, and to model influences unique to both objective and subjective attention measures in the form of bifactor models; in this way, we could assess whether the general factor demonstrated incremental validity beyond the measurement-specific factors.

## Study 1

### Methods

We analyzed data from Unsworth et al. (2021), a study on individual differences in attention lapses. Details of our preregistration are available on the Open Science Framework (https://osf.io/xeu63/). Full-information maximum likelihood estimation was used for missing data (see Unsworth et al., 2021, for details and sample demographics).

#### Subjects

Three hundred fifty-eight subjects from the University of Oregon were individually tested in a 2-h session.

#### Tasks and Materials

**Objective Attention Consistency Indicators.** For each objective indicator task, we present multiple dependent measures that theoretically should reflect variation in attention consistency; we describe our preregistered procedures for selecting among these dependent variables for analysis below, under “Selection of Objective Attention Consistency and Processing Speed Indicators.” For each task, we first list our a priori measure, while also considering different measurement approaches and dependent variables across tasks (i.e., not choosing RTsd as the primary measure for all tasks). We set these a priori measures as the primary indicator for each task and assessed reliability, distribution characteristics, and bivariate correlations of the secondary measures against them; that is, we planned to use only the a priori measure for each task if all other measures were redundant with it. We preregistered that any measures correlated  ≥ 0.70 would be considered redundant and thus would only retain the a priori measure for each task. Measures that correlated  < 0.70 would be retained and included in structural models, as they may reflect different types or degrees of momentary failures of sustained attention.

**Psychomotor Vigilance Task (PVT).** Subjects were presented with a row of zeros onscreen. After an unpredictable period (from 2 to 10 s), the zeros began counting up in 17 ms intervals. The goal of the task was to press the spacebar as quickly as possible to stop the numbers. The RT was displayed for 1 s to provide feedback. The task lasted for 10 min (roughly 75 trials). The potential dependent variables derived from this task will be average RT of the slowest 20% of trials, number of lapses (RTs > 500 ms), intra-individual standard deviation of all RTs (RTsd), intra-individual median absolute deviation of all RTs (RTmad), and the *τ* estimate from an ex-Gaussian model of all RTs.

**Semantic SART.** Subjects were instructed to respond quickly by pressing the spacebar to frequently presented non-target stimuli from one category (animals, presented on 89% of trials) while withholding responses to infrequent target stimuli from a different category (vegetables, presented on 11% of trials). Stimuli were presented for 300 ms followed by a 900 ms mask. There were 315 trials, 35 of which were no-go targets. The potential dependent variables derived from this task will be intra-individual RTsd to correct “go” trials, intra-individual RTmad to correct “go” trials, omission errors on “go” trials, average RT of the slowest 20% of correct “go” trials, RMSSD to correct “go” trials, the *τ* estimate from an ex-Gaussian model using correct “go” trials, and fastest 20% of correct “go” trials.^2^

**Choice RT (CRT).** Subjects responded as quickly as possible to a stimulus (a white cross) in one of four horizontally spaced locations onscreen. The cross appeared after a random interval (300–550 ms in 50 ms increments) and could not appear in the same location on consecutive trials. Subjects indicated the location of the cross by pressing one of four keys on the keyboard (F, G, H, J) mapped to the four locations. Subjects completed 15 practice trials and 210 real trials. The potential dependent variables derived from this task will be the *τ* estimate from an ex-Gaussian model of correct trials, the number of “blocks,” defined as RTs that were twice each individual’s mean RT (Bills, 1931, 1935; see also Bertleson & Joffe, 1963), intra-individual RTsd to correct trials, intra-individual RTmad to correct trials, average RT of slowest 20% of correct trials, and RMSSD to correct trials.

**Continuous Tracking.** Subjects saw a small black circle moving against a gray background onscreen. The goal was to follow the black circle as closely as possible with the mouse cursor. Each block began with a screen saying, “Please focus on the dot,” for 3 s. The circle moved in a pseudorandom fashion within a centered 400 × 440 pixel region. The circle moved at a constant speed in vertical, horizontal, or diagonal directions. Subjects completed a 30 s practice block, followed by (in a random order) one 30 s and one 120 s block, and two 60 and 90 s blocks. The potential dependent variables derived from this task will be tracking distance variability (calculated as a moving window average tracking error in pixels of 5 trials), the number of flat spots (instances where subjects stopped responding for at least 1.5 s), overall average tracking error (i.e., the distance between the cursor and the circle in pixels on each trial across each block), and intra-individual standard deviation in tracking error (calculated as the standard deviation of the distance, in pixels, between the circle location and the cursor location). Tracking distance variability, overall average tracking error, and intra-individual standard deviation in tracking error will be calculated at the block level first and then averaged for each subject to account for tracking duration differences of each block.^3^

**Subjective Attention Consistency Indicators.** Subjects responded to thought probes in four tasks: the PVT (15 probes), the SART (21 probes), a working memory task (8 probes), and Stroop task (12 probes). The probes asked subjects to classify their immediately preceding thoughts into one of five categories. Subjects reported via keypress whether their conscious experience was: (1) *I am totally focused on the current task*, (2) *I am thinking about my performance on the task, (3) I am distracted by sights/sounds/physical sensations*, (4) *I am daydreaming/my mind is wandering about things unrelated to the task*, or (5) *My mind is blank.* Consistent with Unsworth et al. (2021), we operationalized TUTs as the proportion of responses 3–5.

**Working Memory Capacity (WMC) tasks.** Subjects completed three complex span tasks of WMC. For each complex span task, subjects completed three practice stages: the first provided practice in memorizing small sets of the memoranda for each task (e.g., letters or grid locations); the second practice was for processing-only (e.g., math equations, symmetry decisions, sentence comprehension). RTs were recorded during this processing-only practice for each subject. During the real trials, if a processing decision was not made within 2.5 SDs of the processing-only mean, that trial was counted as a processing error; the third practice consisted of both the memory and processing task combined (as in the real trials).

**Operation Span.** Subjects verified whether math operations were true or false while trying to remember a set of letters. After each math operation, a letter was presented for 1 s, and then the next math operation was presented. At the end of the set, subjects were asked to recall the letters from the set by clicking the letters onscreen in the presented serial order. Subjects were granted credit only if the item letters were recalled in the correct serial position. Set sizes ranged from 3 to 7 items and each set size was presented twice (for a max score of 50). Higher scores reflected better recall.

**Symmetry Span.** Subjects verified whether an abstract image presented in an 8 × 8 matrix was symmetrical along the vertical axis. Following the verification, they were presented with a red square for 650 ms in a 4 × 4 grid for memory. At the end of each set, subjects recalled the location of each red square presented; subjects earned credit for items recalled in correct serial position. Set sizes ranged from 2 to 5 items and each set size was presented twice (for a max score of 28). Higher scores reflected better recall.

**Reading Span.** Subjects decided whether sentences made sense or not while remembering a set of letters. Sentences were made nonsensical by altering one word. After deciding whether a sentence made sense, subjects saw the to-be-remembered letter for 1 s. After the final letter of the set, subjects recalled the set; subjects earned credit for items recalled in correct serial position. Set sizes ranged from 3 to 7 items, and each set size was presented twice (for a max score of 50). Higher scores reflected better recall.

**Attention Control Tasks.** Subjects completed three tasks measuring attention control.

**Antisaccade.** Subjects completed 60 trials in which they were told to direct their focus away from a flashing cue (a white flashing “=”) to identify a masked letter (B, P, or R) presented briefly to the opposite side of the screen. The flashing cue and target letter location were 12.7 cm to the left or right of central fixation. The target stimuli appeared onscreen for 100 ms and then were masked (by an *H* for 50 ms then an 8, which remained onscreen until response). Subjects pressed the corresponding key on the numeric keyboard (4, 5, and 6 were used for B, P, and R, respectively) to identify the target letter. Before completing the antisaccade trials, subjects completed 10 response-mapping trials and 10 prosaccade trials (where the flashing cue and letter appeared on the same side). The dependent variable was the proportion of correct antisaccade trials.

** Cued Visual Search.** Subjects decided whether a target F located in a 5 × 5 array of 25 letters (comprised of distractors including forward and backward Es and rotated Ts) was either normal facing (by pressing the “/” key) or mirror-reversed (by pressing the “Z” key). Subjects first completed 8 response-mapping trials. On each trial, subjects received a central arrow cue (500 ms) indicating which two or four possible locations (of eight) the target F could appear in. Following the cue, a blank screen (50 ms) appeared before the 5 × 5 grid of 25 possible locations appeared as dots for 1500 ms, followed by another 50 ms blank screen. Finally, the array of 25 letters was shown, at which time subjects responded to the target F (the array was shown until response, but no longer than 4000 ms). Other Fs also appeared in uncued, nontarget locations as distractors, and so to respond correctly, subjects must selectively maintain focus on the cued locations. Subjects completed 8 practice trials followed by 80 scored trials. Cue type, target direction and location were all randomly and equally presented during the scored trial block. The dependent measure was mean RT for correct responses.

**Stroop.** Subjects were presented with a color word (red, green, or, blue) in one of three different font colors (red, green, or blue). The goal of the task was to indicate the font color as quickly and accurately as possible via key press (1 = red, 2 = green, 3 = blue). Subjects completed 15 response-mapping practice trials and 6 practice trials of the real task. Subjects then completed 100 scored trials (67 congruent trials [e.g., the word “red” was presented in red font color]; 33 incongruent trials [e.g., the word “green” presented in blue font color]). The dependent measure was the Stroop RT effect (correct incongruent RT—correct congruent RT).

**P﻿rocessing Speed.** As in Unsworth et al. (2021), we assessed processing speed in three tasks where RT was one of the primary measures recorded (the PVT, Stroop, and CRT). In these tasks, RTs were ranked from fastest to slowest and the fastest 20% of trials were used as indicators. Here, however, in addition to using RT for the fastest 20% of trials, processing speed will also be calculated using the μ parameter from the ex-Gaussian model from the SART, PVT, Stroop, and CRT (reflecting the mean of the Gaussian component). Additionally, Median RT of the 10 prosaccade practice trials in the antisaccade task will also be used as a measure of processing speed. Selection of processing speed measures from each task for analyses followed a similar approach to the selection of objective attention consistency measures (see “Selection of Objective Attention Consistency and Processing Speed Indicators”).

**Cognitive Failures Questionnaire—memory and attention lapses (CFQ-MAL).** Subjects responded to 40 questionnaire items about their everyday memory and attention lapses. Subjects indicated via keypress that they experienced such failures on the following scale: (1) *never*, (2) *rarely*, (3) *once in a while*, (4) *often*, (5) *very often*. The dependent variable was an item sum score.

**Non-cognitive predictor measures.** Subjects completed the following self-report scales.

**Motivation and Alertness.** Following the completion of four tasks (PVT, CRT, continuous tracking, and antisaccade) subjects responded to one question each about their motivation and alertness on a 1–6 scale (higher scores meaning more motivated or alert). Specifically, they were asked “How motivated were you to perform well on the task?” and “How alert do you feel right now?”.

**Big Five Inventory (BFI).** Subjects completed a 44-item version of the Big Five personality inventory. Extraversion was assessed by eight items, agreeableness by nine, conscientiousness by nine, neuroticism by eight, and openness by 10. Each item asked the subject to respond based on how well it described them using a 5-point scale (1 = disagree strongly, 5 agree strongly). The dependent variable was the average rating across items for each factor.

#### Procedures

After providing informed consent, subjects completed the cognitive battery in the following order: operation span, symmetry span, reading span, antisaccade, cued visual search, PVT, Stroop, SART, choice RT, continuous tracking, and whole-report visual WM. Following completion of the cognitive tasks, subjects completed questionnaire measures in the following order: BFI, Boredom Proneness Scale, CFQ-MAL, Mindful Attention Awareness Scale, and self-reported sleep quality and quantity.

### Results

Below we report the results of our preregistered analyses and note explicitly wherever we deviated from the preregistered plan. Data and Rmarkdown files for all analyses are available on the Open Science Framework (https://osf.io/xeu63/). For all latent variable correlations, effect sizes are discussed in the traditional Cohen (1988) framework where *r* = 0.10, 0.30, and 0.50 are cut-offs for small, moderate, and large effect sizes, respectively.

### Data Analysis Exclusions

Consistent with Unsworth et al. (2021), we excluded the same subject data from the PVT (*n* = 2), Stroop (*n* = 1), and Choice RT tasks (*n* = 1) for having extremely long *M* RTs in each task (in the PVT, one subject had *M* RT > 1200 ms and one had *M* RT  > 18 s; for Stroop, the subject had *M* RT  > 2400 ms; for the Choice RT task, the subject had *M* RT  > 1200 ms). Also following Unsworth et al. (2021), we dropped 16 subjects’ data from the SART for having  > 50% omission errors. Prior to calculating any of the DVs for the current study, we calculated “go” trial accuracy for the remaining subjects and identified 3 who had “go” trial accuracy  < 70%. We therefore deviated from Unsworth et al. (2021) and *deviated from our preregistration* by excluding SART data from these subjects, too, as such low accuracy might indicate a failure to understand or comply with task instructions, rather than attention lapses. As preregistered (but deviating from Unsworth et al., 2021), we also dropped subjects’ TUT data from a task if their performance data were dropped from that probed task. Finally, and although *not preregistered* (and not specified in Unsworth et al., 2021), we also dropped Choice RT task data from two subjects with 0% accuracy, indicating they did not follow or understand task instructions.

### RT Cleaning Procedures

In tasks where RT was the primary measure of interest (e.g., objective attention consistency indicator tasks and processing speed tasks), we implemented a preregistered multistep procedure for trial-level cleaning. First, we identified and removed RTs for error and post-error trials (and, in tasks that included thought probes, post-probe trials). Next, we removed RTs for trials that were likely anticipations (i.e., RTs < 200 ms). From the remaining trials, we next calculated for each subject, in each task, a value equal to their Median RT + 3*IQR. Any trials outside of this value were replaced with this value. Details on the average number of trials cleaned in each task can be found in Appendix A.

Finally, we calculated the number of usable trials each subject had following our RT cleaning protocol. As preregistered, we dropped task data for subjects who did not have at least 40 trials and thus could not reliably contribute to our primary measures of interest. This resulted in 6 additional subjects’ data being dropped for the PVT only.

### Selection of Objective Attention Consistency and Processing Speed Indicators

After trial-level cleaning, we calculated all possible DVs of interest for each task. As preregistered, for each DV, we used the Median + 3*IQR rule that we had applied to trial-level data to censor outlying subjects (replacing outlying subjects’ data with a value equal to the Median + 3*IQR for each DV). Supplemental Tables 1 and 2 presents the descriptive statistics for each possible DV, for each task, as well as the number of subjects censored for each measure. Note that many of the measures originally used in Unsworth et al. (2021) had potentially problematic skewness and kurtosis, but after our trial- and subject-level cleaning procedures, these values were acceptable for all potential dependent measures.

As preregistered, our first step in selecting which DVs from each task to include in our structural models focused on examining the univariate distributions for possible issues of skewness and kurtosis. Per the guidelines suggested by Kline (2011), problematic skew was identified as > 3.0 and problematic kurtosis was identified as > 10.0. No variables were removed from consideration for problematic distributions.

We next examined the reliability of the measures. Consistent with Unsworth et al. (2021), we calculated split-half reliability for each measure where applicable. (Note that in the PVT and Stroop, splitting the task resulted in  < 40 trials in each grouping, which prohibited reliable estimation of ex-Gaussian models; we thus do not report reliability for PVT *τ*, PVT μ, or Stroop μ). We preregistered that any measures with poor split-half reliability (< 0.50) would not be considered for models, but no variables needed to be removed from consideration for poor reliability.

We next examined within-task bivariate correlations to see whether any measure combinations captured different degrees of attention consistency failure than the a priori measure (preregistered criterion for redundancy: *r* ≥ 0.70). As seen in Supplemental Table 3, many proposed measures were redundant (for a similar result in the PVT see Unsworth et al., 2020). For the PVT, CRT, and Continuous Tracking Task, we retained only the a priori measure (*M* RT of the slowest 20% for the PVT, *τ* for the CRT, and the Tracking Variability measure for Continuous Tracking). For the SART, however, correlations suggested that several measures reflected differing degrees or types of attention lapses (Cheyne et al., 2009; Unsworth et al., 2021). Thus, by our selection criteria, we would retain not only SART RTsd (a priori), but also *τ*, *M* RT from the fastest 20% of trials, and Omissions. We had not expected to find evidence for four nonredundant measures from the SART, particularly while finding no additional non-a priori measures from the other tasks. To avoid oversaturating the objective attention consistency latent variable with SART measures—with four SART indicators but only one indicator each from the other tasks—we retained only SART RTsd, *τ*, and Omissions for all structural models (*thus deviating from preregistration*).^4^

For processing speed, our proposed measures showed good split-half reliability and distributional characteristics. To diversify speed factor indicators (and prevent overlap with other DVs for other factors), we selected the following: *μ* from the PVT, *M* RT of the fastest 20% of trials for the CRT and the Stroop, and Median RT of the Prosaccade practice trials; *this deviated from the preregistration*, which indicated using *M* RT of the fastest 20% of trials for the PVT. Supplemental Table 4 provides the bivariate correlations among the possible speed of processing measures for each task. (Note that we also *deviated from preregistration* by not including SART μ, given the large number of SART indicators we included as objective attention consistency indicators and given its poor zero-order correlations with the other processing speed measures.)

### Multivariate Outliers

As preregistered, once we established our primary indicators, we checked for multivariate outliers in the final dataset. To do this, we used the *Routliers* package (Leys et al., 2013) to calculate Mahalanobis distance for each observation. This analysis indicated there were 11 multivariate outliers in the dataset (~ 3% of subjects). These subjects’ data were removed case-wise before conducting structural modeling.

### Descriptive Statistics and Correlations for Final Dataset

Table 2 provides descriptive statistics for the final dataset. Unsworth et al. (2021) reported adequate to strong reliabilities for many of their measures of WMC (0.64–0.76), Attention Control (0.48–0.87; the lowest reliability occurring with the Stroop RT difference score), and TUT rates (0.60–0.89). Reliabilities for the new objective attention consistency and processing speed measures used in the final dataset were also highly reliable (> 0.85). Table 3 reports bivariate correlations among all measures of interest. Consistent with Unsworth et al. (2021), measures from the same putative construct (e.g., WMC, attention control, and motivation) all correlated more strongly with each other than with measures of other constructs. Importantly, our newly selected objective attention consistency indicators also showed evidence of convergent validity (median |*r|*= 0.30), suggesting that subjects who showed more variable responding in one task also tended to do so in other tasks.

**Table 2:** Descriptive statistics for Study 1 measures

Construct/Measure	Mean	SD	Min	Max	Skew	Kurtosis	*N*
**Objective Attention Consistency**							
PVT Bin 5	455.01	94.34	307.08	789.01	1.35	2.34	333
SART RTsd	132.35	48.48	44.07	299.83	1.09	1.80	322
SART Omissions	18.48	14.23	0.00	70.00	1.36	2.17	322
SART *τ*	100.64	67.60	0.00	328.59	0.95	1.23	322
CRT *τ*	90.06	36.29	26.32	229.52	1.22	2.11	335
Continuous Tracking Variability	1.14	0.41	0.24	2.56	0.70	0.62	322
**Subjective Attention Consistency**							
PVT TUTs	0.43	0.29	0.00	1.00	0.23	− 0.92	333
SART TUTs	0.44	0.33	0.00	1.00	0.24	− 1.19	322
WRWM TUTs	0.53	0.37	0.00	1.00	− 0.12	− 1.43	271
Stroop TUTs	0.21	0.28	0.00	1.00	1.42	1.01	341
**Working Memory Capacit﻿y**							
OPERSPAN	38.00	8.04	10.00	50.00	− 0.70	0.11	345
READSPAN	37.41	8.48	1.00	50.00	− 1.04	1.36	346
SYMSPAN	18.85	5.19	2.00	28.00	− 0.48	− 0.12	346
**Attention C﻿ontrol**							
Antisaccade Accuracy	0.60	0.15	0.25	0.93	0.03	− 0.60	337
Cued Visual Search RT	1276.70	290.03	596.24	2316.29	0.64	0.42	344
Stroop RT	147.81	97.70	− 224.04	509.78	0.60	1.13	341
**Processing S﻿peed**							
PVT μ	286.22	28.01	227.26	380.01	0.61	0.09	333
CRT Bin 1	293.81	35.52	227.19	434.92	1.03	1.48	335
Stroop Bin 1	437.86	63.46	304.45	675.58	0.93	1.35	340
Prosaccade *M* RT	703.05	240.33	305.50	1628.00	1.04	1.54	311
**Alertness**							
PVT	3.30	1.28	1.00	6.00	0.13	− 0.55	341
CRT	3.31	1.33	1.00	6.00	0.08	− 0.71	338
Continuous Tracking	2.31	1.43	1.00	6.00	0.79	− 0.48	314
Antisaccade	3.64	1.29	1.00	6.00	− 0.01	− 0.78	337
**Motivation**							
PVT	4.00	1.31	1.00	6.00	− 0.40	− 0.53	341
CRT	4.03	1.37	1.00	6.00	− 0.54	− 0.35	338
Continuous tracking	2.70	1.60	1.00	6.00	0.39	− 1.16	314
Antisaccade	4.00	1.35	1.00	6.00	− 0.39	− 0.61	337
**Dispositional M﻿easures**							
Openness	3.57	0.58	1.80	4.90	− 0.12	− 0.10	274
Conscientiousness	3.61	0.63	1.22	4.89	− 0.49	0.47	274
Extraversion	3.22	0.87	1.14	4.57	− 0.38	− 0.80	274
Agreeableness	3.92	0.64	1.67	5.00	− 0.68	0.57	274
Neuroticism	3.16	0.86	1.00	5.00	− 0.00	− 0.76	274
Cognitive Failures	111.01	26.16	52.00	191.00	0.26	− 0.17	274

*PVT* = psychomotor vigilance task. *SART* = sustained attention to response task. *CRT* = choice reaction time task. *WRWM* = whole-report working memory task. *TUTs* = rate of task-unrelated thoughts in specified task. *OPERSPAN* = operation span. *READSPAN =* reading span. *SYMSPAN* = symmetry span. *Bin 5* = mean RT of slowest 20% of correct trials. *RTsd* = intra-individual RT variability. *Bin 1* = mean RT of fastest 20% of correct trials.* μ* = Mu from ex-Gaussian model

**Table 3:** Zero-order correlations among variables for structural models in Study 1

Variable	1	2	3	4	5	6	7	8	9	10	11	12	13	14	15	16
1. PVT Bin 5	1															
2. SART RTsd	0.31	1														
3. SART Omissions	0.21	0.26	1													
4. SART *τ*	0.26	0.65	0.39	1												
5. CRT *τ*	0.36	0.17	0.33	0.15	1											
6. Continuous Tracking Variability	0.37	0.30	0.34	0.22	0.38	1										
7. PVT TUTs	0.38	− 0.03	− 0.04	0.04	0.12	0.12	1									
8. SART TUTs	0.14	0.11	0.26	0.17	0.14	0.24	0.39	1								
9. WRWM TUTs	0.11	0.07	0.05	0.08	0.04	0.13	0.40	0.55	1							
10. Stroop TUTs	0.20	0.07	0.02	0.06	0.13	0.20	0.50	0.48	0.35	1						
11. OPERSPAN	− 0.04	− 0.09	− 0.20	− 0.11	− 0.14	− 0.19	− 0.05	− 0.04	0.00	− 0.09						
12. READSPAN	− 0.20	− 0.12	− 0.15	− 0.17	− 0.09	− 0.20	− 0.07	− 0.05	− 0.10	− 0.05	1					
13. SYMSPAN	− 0.17	− 0.15	− 0.17	− 0.10	− 0.14	− 0.22	− 0.14	− 0.13	0.00	− 0.17	0.49	1				
14. Antisaccade	− 0.36	− 0.26	− 0.22	− 0.22	− 0.31	− 0.29	− 0.13	− 0.08	0.03	− 0.09	0.42	0.36	1			
15. Cued Visual Search RT	0.24	0.24	0.18	0.15	0.34	0.34	0.08	0.07	− 0.03	0.15	0.17	0.14	0.22	1		
16. Stroop RT	0.04	− 0.03	− 0.04	0.05	− 0.05	0.01	0.03	− 0.07	− 0.03	0.05	− 0.20	− 0.06	− 0.24	− 0.30	1	
17. PVT μ	0.34	0.13	0.05	0.11	0.22	0.09	0.31	0.01	0.08	0.08	− 0.22	− 0.16	− 0.12	− 0.14	0.10	1
18. CRT Bin 1	0.13	0.09	0.03	− 0.05	0.29	0.11	0.02	− 0.06	0.05	0.06	− 0.12	− 0.12	− 0.17	− 0.24	0.11	0.06
19. Stroop Bin 1	0.32	0.19	0.12	0.08	0.31	0.21	0.13	0.02	0.05	0.20	− 0.05	− 0.08	− 0.18	− 0.19	0.25	0.01
20. Prosaccade *M* RT	0.06	0.25	0.23	0.18	0.17	0.10	0.02	0.09	− 0.07	0.13	− 0.16	− 0.15	− 0.24	− 0.26	0.33	0.18
21. PVT Alertness	− 0.40	− 0.19	− 0.14	− 0.23	− 0.17	− 0.29	− 0.57	− 0.41	− 0.38	− 0.40	− 0.19	− 0.05	− 0.19	− 0.30	0.38	0.06
22. CRT Alertness	− 0.15	− 0.13	− 0.06	− 0.12	− 0.15	− 0.14	− 0.39	− 0.33	− 0.31	− 0.36	0.04	0.15	0.18	0.23	− 0.17	− 0.09
23. Continuous Tracking Alertness	− 0.08	− 0.06	− 0.10	− 0.06	− 0.07	− 0.23	− 0.35	− 0.27	− 0.25	− 0.23	0.07	0.10	0.13	0.07	− 0.08	− 0.06
24. Antisaccade Alertness	− 0.21	− 0.08	− 0.10	− 0.08	− 0.13	− 0.20	− 0.36	− 0.25	− 0.27	− 0.27	0.07	− 0.01	0.14	0.09	− 0.18	0.05
25. PVT Motivation	− 0.36	− 0.13	− 0.10	− 0.14	− 0.15	− 0.24	− 0.46	− 0.31	− 0.25	− 0.28	0.04	0.07	0.13	0.30	− 0.15	− 0.08
26. CRT Motivation	− 0.18	− 0.17	− 0.10	− 0.15	− 0.21	− 0.20	− 0.28	− 0.25	− 0.30	− 0.29	0.02	0.13	0.13	0.12	− 0.10	− 0.04
27. Continuous Tracking Motivation	− 0.08	− 0.07	− 0.14	− 0.10	− 0.05	− 0.28	− 0.25	− 0.25	− 0.22	− 0.20	0.13	0.16	0.15	0.08	− 0.07	− 0.10
28. Antisaccade Motivation	− 0.28	− 0.18	− 0.12	− 0.14	− 0.15	− 0.18	− 0.30	− 0.26	− 0.24	− 0.22	0.04	0.01	0.16	0.04	− 0.16	0.06
29. Openness	− 0.11	0.02	0.02	0.07	0.06	− 0.09	− 0.09	− 0.04	0.00	− 0.07	0.04	0.04	0.13	0.30	− 0.15	− 0.14
30. Conscientiousness	0.05	0.03	− 0.03	0.02	− 0.02	0.03	− 0.16	− 0.11	− 0.09	− 0.19	− 0.02	0.02	0.01	0.04	0.05	0.00
31. Extraversion	0.06	0.04	0.11	− 0.01	0.07	0.10	− 0.03	0.03	0.05	− 0.04	− 0.09	0.01	− 0.05	− 0.08	− 0.04	0.04
32. Agreeableness	0.00	0.00	0.10	0.01	0.07	0.02	− 0.14	− 0.09	− 0.08	− 0.12	− 0.02	0.03	− 0.02	− 0.04	− 0.04	− 0.13
33. Neuroticism	0.12	− 0.03	0.02	− 0.03	0.11	0.07	0.23	0.08	− 0.02	0.15	− 0.07	0.03	0.06	− 0.09	0.00	0.06
34. Cognitive Failures	0.12	0.07	0.09	0.07	0.05	0.12	0.20	0.14	0.07	0.09	− 0.09	− 0.08	− 0.18	− 0.08	0.18	0.05

Variable	17	18	19	20	21	22	23	24	25	26	27	28	29	30	31	32	33
1. PVT Bin 5																	
2. SART RTsd																	
3. SART Omissions																	
4. SART *τ*																	
5. CRT *τ*																	
6. Continuous Tracking Variability																	
7. PVT TUTs																	
8. SART TUTs																	
9. WRWM TUTs																	
10. Stroop TUTs																	
11. OPERSPAN																	
12. READSPAN																	
13. SYMSPAN																	
14. Antisaccade																	
15. Cued Visual Search RT																	
16. Stroop RT																	
17. PVT μ	1																
18. CRT Bin 1	0.34	1															
19. Stroop Bin 1	0.39	0.57	1														
20. Prosaccade *M* RT	− 0.05	0.16	0.19	1													
21. PVT Alertness	− 0.18	− 0.01	− 0.15	− 0.02	1												
22. CRT Alertness	− 0.09	− 0.04	− 0.10	− 0.07	0.54	1											
23. Continuous Tracking Alertness	− 0.01	0.03	− 0.01	− 0.24	0.43	0.49	1										
24. Antisaccade Alertness	− 0.09	− 0.05	− 0.10	− 0.03	0.53	0.32	0.28	1									
25. PVT Motivation	− 0.11	0.03	− 0.12	0.02	0.62	0.37	0.33	0.42	1								
26. CRT Motivation	− 0.07	0.00	− 0.10	− 0.08	0.38	0.67	0.34	0.28	0.55	1							
27. Continuous Tracking Motivation	0.05	0.03	0.00	− 0.21	0.36	0.36	0.79	0.21	0.44	0.38	1						
28. Antisaccade Motivation	− 0.06	− 0.07	− 0.10	− 0.13	0.40	0.26	0.21	0.59	0.47	0.33	0.25	1					
29. Openness	− 0.03	− 0.06	− 0.03	− 0.01	0.11	0.14	0.12	0.04	0.02	0.04	0.07	0.02	1				
30. Conscientiousness	0.04	0.00	0.03	− 0.03	0.12	0.12	0.09	0.08	0.06	0.16	0.15	0.03	− 0.04	1			
31. Extraversion	0.04	− 0.03	0.00	− 0.09	0.01	0.09	0.09	0.01	0.01	0.02	0.10	0.06	0.15	0.10	1		
32. Agreeableness	0.11	− 0.02	− 0.02	0.04	0.13	0.24	0.08	0.08	0.09	0.17	0.07	0.07	− 0.01	0.25	0.11	1	
33. Neuroticism	0.03	− 0.03	0.09	0.03	− 0.08	− 0.09	− 0.05	− 0.10	− 0.03	− 0.03	− 0.04	− 0.07	− 0.03	− 0.19	− 0.26	− 0.32	1
34. Cognitive Failures	− 0.03	− 0.06	− 0.01	− 0.02	− 0.10	− 0.08	− 0.08	− 0.06	0.01	− 0.08	− 0.13	− 0.03	0.01	− 0.40	− 0.05	− 0.19	0.40

*PVT* = psychomotor vigilance task, *SART* = sustained attention to response task, *CRT* = choice reaction time task, *WRWM* = whole-report working memory task, *TUTs* = rate of task-unrelated thoughts in specified task, *OPERSPAN =* operation span, *READSPAN =* reading span, *SYMSPAN =* symmetry span, *Bin* 5 = mean RT of slowest 20% of correct trials, *RTsd* intra-individual RT variability, *Bin 1* = mean RT of fastest 20% of correct trials, *μ* = Mu from ex-Gaussian model

### Measurement Models of Attention Consistency

As preregistered, our first set of analyses attempted to simply replicate the latent variable correlation between objective and subjective indicators of attention consistency reported by Unsworth et al. (2021). We first tested a 2-factor model with separate latent variables for objective (i.e., RT variability and omissions) and subjective (i.e., TUT reports) indicators; these latent variables were allowed to correlate. As seen in Table 4, the model adequately fit the data. *Although not preregistered*, we included residual correlations among any performance and TUT indicators from the same task (e.g., PVT Slowest 20% with PVT TUTs); a model without these residuals did not adequately fit the data and we retained these residual correlations for all subsequent models. Our measures and analysis conceptually replicated the lapse–TUT correlation in Unsworth et al. (2021), although this relationship was slightly weaker here (*r* = 0.32 vs 0.44 in the original study; see Table 5 for factor loadings).^5^ Again, this moderate*—*but not strong*—*correlation confirms that these two indicator types of attention consistency are not redundant. Instead, as we’ve argued, each indicator type may reflect different degrees of disengagement and each is influenced by confounding processes that are unique to that measurement type, so modeling the shared variance among the indicators may provide a more construct valid measure of attention consistency than either objective or subjective measures alone.

**Table 4:** Fit statistics for latent variable models for Study 1

Model	*χ*^2^ (*df*)	*χ*^2^/*df*	CFI	TLI	RMSEA [90% CI]	SRMR
**Measurement Models**						
2-factor	54.362 (27)	2.01	0.967	0.944	0.054 [0.033–0.075]	0.042
True bifactor	–	–	–	–	–	–
Bifactor subjective residual	51.369 (24)	2.14	0.967	0.937	0.058 [0.036–0.079]	0.040
Bifactor objective residual	44.357 (22)	2.02	0.973	0.944	0.054 [0.031–0.077]	0.035
Hierarchical model	54.362 (27)	2.01	0.967	0.944	0.054 [0.033–0.075]	0.042
**Confirmatory Factor Analyses**						
2-factor	760.400 (442)	1.72	0.907	0.882	0.046 [0.040–0.051]	0.055
True bifactor	–	–	–	–	–	–
Bifactor subjective residual	757.589 (439)	1.73	0.907	0.881	0.046 [0.040–0.051]	0.055
Bifactor objective residual	748.092 (437)	1.71	0.909	0.883	0.045 [0.040–0.051]	0.054
Full hierarchical	850.309 (453)	1.88	0.884	0.856	0.050 [0.045–0.056]	0.067
Reduced predictor hierarchical	324.822 (190)	1.71	0.914	0.886	0.045 [0.037–0.054]	0.057

**Table 5:** Standardized factor loadings (and standard errors) for latent variable measurement models for Study 1

Construct and Measure	M﻿odel Name			
	Two Factor Measurement	Bifactor Sub-Res Measurement	Bifactor Obj-Res Measurement	Hierarchical Measurement
**Attention** **Consistency**				
PVT Bin 5		0.62 (0.05)	0.24 (0.06)	
SART RTsd		0.46 (0.06)	0.06 (0.07)	
SART Omissions		0.49 (0.06)	0.05 (0.07)	
SART *τ*		0.37 (0.06)	0.11 (0.07)	
CRT *τ*		0.58 (0.05)	0.18 (0.06)	
Continuous Tracking Variability		0.66 (0.05)	0.28 (0.06)	
PVT TUTs		0.17 (0.07)	0.64 (0.05)	
SART TUTs		0.28 (0.07)	0.74 (0.04)	
WRWM TUTs		0.14 (0.07)	0.65 (0.04)	
Stroop TUTs		0.24 (0.06)	0.67 (0.04)	
**Objective**/**Objective**^**resid**^				
PVT Bin 5	0.62 (0.05)		0.57 (0.05)	0.62 (0.05)
SART RTsd	0.46 (0.06)		0.48 (0.06)	0.46 (0.06)
SART Omissions	0.48 (0.06)		0.51 (0.06)	0.48 (0.06)
SART *τ*	0.37 (0.06)		0.36 (0.07)	0.37 (0.06)
CRT *τ*	0.58 (0.05)		0.54 (0.05)	0.58 (0.05)
Continuous Tracking Variability	0.66 (0.05)		0.60 (0.05)	0.66 (0.05)
**Subjective**/**Subjective**^**resid**^				
PVT TUTs	0.61 (0.05)	0.59 (0.05)		0.61 (0.05)
SART TUTs	0.76 (0.04)	0.70 (0.05)		0.76 (0.04)
WRWM TUTs	0.66 (0.05)	0.66 (0.05)		0.66 (0.05)
Stroop TUTs	0.67 (0.04)	0.62 (0.05)		0.67 (0.04)

*Bifactor Sub-Res* = bifactor model with a subjective-indicator residual factor, *Bifactor Obj-Res =* bifactor model with an objective indicator residual factor, *OPERSPAN* = operation span, *READSPAN* = reading span, *SYMMSPAN* = symmetry span, *PVT Bin 1 = * mean RT of the fastest 20% of trials in the PVT, *PVT Bin 5* = mean RT of the slowest 20% of trials in the PVT, *SART RTsd* = intrasubject standard deviation in RT from SART, *PVT* = psychomotor vigilance task, *SART =* sustained attention to response task, *CRT* = choice reaction time task, *WRWM* = whole-report working memory task, *TUTs* = TUT rate from task,  *μ* = Mu from ex-Gaussian model

Our next preregistered measurement model was a bifactor model, which attempted to account for common variance across all the objective and subjective indicators of attention consistency, while also modeling residual shared variance that was unique to each indicator type. Unfortunately, there were signs of misfit (e.g., warnings of negative error variances), so we could not successfully fit a full bifactor model.

As preregistered, then, we next attempted to fit separate bifactor models where each had only one residual factor modeled (e.g., a common attention consistency factor plus a residual objective indicator factor, with no residual TUT factor). As seen in Table 4, each of these models adequately fit the data. Table 5 presents the factor loadings for each model. In the Subjective-Residual model, all indicators aside from one (TUT rate from the WM task) significantly loaded onto the general Attention Consistency factor, although the TUT rate loadings were weak. Additionally, there was enough remaining shared variance in TUT reports to successfully model a Subjective-Residual factor. In the Objective-Residual model, many of the indicators significantly loaded onto the general Attention Consistency variable, but none of the objective SART indicators did, and all performance indicator loadings were weak. After accounting for general attention consistency, there was still enough shared variance left over to successfully model an objective residual latent variable. Thus, in these two separate models, we were able to assess general ability to sustain attention consistency as the individual-differences overlap among objective and subjective measures.

We emphasize, however, that the loadings on the general factor were heavily favored by the “absent-residual” factor in each model. In the Subjective-Residual model, the general factor reflected mostly variance from the objective indicators, and in the Objective-Residual model, the general factor mostly reflected the subjective indicators. This imbalance of factor-loading weights will impact correlations between the “general” factor and other constructs (see below), and they suggest that these reduced bifactor models might inadequately describe the data, despite reasonable global fit indices (Bornovalova et al., 2020).

### Confirmatory Factor Analyses of Individual Differences in Attention Consistency

Our next set of preregistered analyses assessed correlations between our nomological-net predictor constructs with attention consistency factors. While our focus was on the bifactor models, we first present correlations between our predictors and the 2-factor model to replicate Unsworth et al. (2021). A model with latent variables for WMC, Attention Control, Processing Speed, Motivation, Alertness, and manifest variables for openness, conscientiousness, extraversion, agreeableness, neuroticism, and cognitive failures adequately fit the data (Table 4) and all predictor indicators loaded onto their respective constructs [see Table 6; as in Unsworth et al. (2021), we fixed the loadings of the dispositional manifest variables equal to one]. We note, however, that the TLI for this model, and for all subsequent structural models that included predictor constructs, was just below the minimum cut-off for adequate fit. We therefore interpret these CFA models with some caution and discuss implications of these findings in the Study 1 Discussion.

**Table 6:** Standardized factor loadings (and standard errors) for latent variable confirmatory factor analysis (CFA) models for Study 1

Construct and Measure	M﻿odel Name			
	Two factor CFA	Bifactor Sub-Res CFA	Bifactor Obj-Res CFA	Hierarchical CFA
**Working Memory Capacity**				
OPERSPAN	0.71 (0.05)	0.71 (0.05)	0.71 (0.05)	0.71 (0.05)
READSPAN	0.64 (0.05)	0.64 (0.05)	0.64 (0.05)	0.65 (0.05)
SYMSPAN	0.62 (0.05)	0.62 (0.05)	0.62 (0.05)	0.61 (0.05)
**Attention Control**				
Antisaccade	0.55 (0.05)	0.55 (0.05)	0.55 (0.05)	
Cued Visual Search	− 0.56 (0.05)	− 0.56 (0.05)	− 0.56 (0.05)	
Stroop	− 0.18 (0.06)	− 0.18 (0.06)	− 0.18 (0.06)	
**Processing Speed**				
CRT Bin 1	0.68 (0.04)	0.68 (0.04)	0.68 (0.04)	0.67 (0.05)
PVT *μ*	0.47 (0.05)	0.47 (0.05)	0.47 (0.05)	0.46 (0.05)
Stroop Bin 1	0.82 (0.04)	0.82 (0.04)	0.82 (0.04)	0.85 (0.04)
Prosaccade *M* RT	0.27 (0.06)	0.27 (0.06)	0.27 (0.06)	0.23 (0.06)
**Alertness**				
PVT	0.84 (0.03)	0.84 (0.03)	0.84 (0.03)	
CRT	0.68 (0.04)	0.68 (0.04)	0.68 (0.04)	
Continuous Tracking	0.55 (0.04)	0.55 (0.04)	0.55 (0.04)	
Antisaccade	0.58 (0.04)	0.58 (0.04)	0.58 (0.04)	
**Motivation**				
PVT	0.82 (0.03)	0.82 (0.03)	0.82 (0.03)	
CRT	0.68 (0.04)	0.68 (0.04)	0.68 (0.04)	
Continuous Tracking	0.53 (0.04)	0.53 (0.04)	0.53 (0.04)	
Antisaccade	0.55 (0.05)	0.55 (0.05)	0.55 (0.05)	
**Attention Consistency**				
PVT Bin 5		0.65 (0.04)	0.29 (0.06)	
SART RTsd		0.46 (0.06)	0.06 (0.06)	
SART Omissions		0.46 (0.06)	0.05 (0.06)	
SART *τ*		0.36 (0.06)	0.11 (0.06)	
CRT *τ*		0.59 (0.05)	0.17 (0.06)	
Continuous Tracking Variability		0.65 (0.04)	0.26 (0.06)	
PVT TUTs		0.25 (0.06)	0.74 (0.04)	
SART TUTs		0.23 (0.06)	0.65 (0.06)	
WRWM TUTs		0.12 (0.07)	0.61 (0.05)	
Stroop TUTs		0.25 (0.06)	0.66 (0.04)	
**Objective**/**Objective**^**resid**^				
PVT Bin 5	0.65 (0.04)		0.58 (0.05)	0.62 (0.05)
SART RTsd	0.47 (0.05)		0.48 (0.06)	0.41 (0.05)
SART Omissions	0.46 (0.05)		0.48 (0.05)	0.48 (0.06)
SART *τ*	0.37 (0.06)		0.35 (0.06)	0.32 (0.06)
CRT *τ*	0.59 (0.05)		0.56 (0.05)	0.60 (0.05)
Continuous Tracking Variability	0.65 (0.04)		0.59 (0.05)	0.64 (0.05)
**Subjective**/**Subjective**^**resid**^				
PVT TUTs	0.73 (0.04)	0.68 (0.04)		0.63 (0.04)
SART TUTs	0.67 (0.04)	0.64 (0.04)		0.75 (0.04)
WRWM TUTs	0.61 (0.05)	0.61 (0.05)		0.65 (0.05)
Stroop TUTs	0.67 (0.04)	0.61 (0.04)		0.70 (0.04)

*Bifactor Sub-Res* = bifactor model with a subjective-indicator residual factor, *Bifactor Obj-Res* = bifactor model with an objective indicator residual factor, *OPERSPAN* = operation span, *READSPAN* = reading span, *SYMMSPAN* = symmetry span, *PVT Bin 1* = mean RT of the fastest 20% of trials in the PVT, *PVT Bin 5* = mean RT of the slowest 20% of trials in the PVT, *SART RTsd* = intrasubject standard deviation in RT from SART, *PVT* = psychomotor vigilance task, *SART* = sustained attention to response task, *CRT* = choice reaction time task, *WRWM* = whole-report working memory task, *TUTs* = TUT rate from task, *μ* = Mu from ex-Gaussian model

*Although not preregistered*, we also included residual correlations between PVT μ with other PVT measures in all models that included processing speed indicators, following from a post-hoc residual correlation added in Unsworth et al. (2021). Table 7 displays correlations with the two attention consistency factors. In general, we replicated the correlations reported in Unsworth et al. (2021), even after changing the data-processing pipeline and some indicators. For example, the objective factor correlated strongly with attention control, (*r* = − 0.86 vs. *r* = − 0.69 in Unsworth et al., 2021) and processing speed (*r* = 0.51 vs. *r* = 0.47. in Unsworth et al., 2021), and the subjective factor correlated strongly with alertness (*r* =  − 0.78 vs. − 0.78 in Unsworth et al., 2021) and motivation (*r* =  − 0.67 vs. − 0.65 in Unsworth et al., 2021).

**Table 7:** Latent variable correlations from Study 1 two-factor model

Construct/Measure	1	2	3	4	5	6	7	8	9	10	11	12
1. Objective Attention Consistency												
2. Subjective Attention Consistency	0.33											
3. WMC	− 0.39	− 0.18										
4. Attention Control	− 0.86	− 0.24	0.50									
5. Processing Speed	0.51	0.18	− 0.33	− 0.67								
6. Alertness	− 0.47	− 0.78	0.19	0.42	− 0.17							
7. Motivation	− 0.52	− 0.64	0.21	0.33	− 0.14	0.77						
8. Openness	− 0.06	− 0.09	0.01	− 0.01	− 0.05	0.15	0.04					
9. Conscientiousness	0.02	− 0.22	− 0.07	− 0.02	0.04	0.14	0.14	− 0.04				
10. Extraversion	0.13	− 0.01	− 0.02	0.02	− 0.00	0.05	0.03	0.15	0.10			
11. Agreeableness	0.04	− 0.18	0.00	− 0.07	0.02	0.19	0.13	− 0.01	0.25	0.11		
12. Neuroticism	0.12	0.21	− 0.18	− 0.25	0.06	− 0.13	− 0.08	− 0.03	− 0.19	− 0.26	− 0.32	
13. Cognitive Failures	0.15	0.21	− 0.01	− 0.09	− 0.04	− 0.12	− 0.08	0.01	− 0.40	− 0.05	− 0.19	0.40

*WMC* = working memory capacity

We next ran two separate nomological network CFAs with each of the reduced bifactor models. We first report results for the Subjective-Residual-only model, and then from the Objective-Residual-only model. In each model, we allowed correlations among the predictor variables to be estimated, and they were consistent across the models and similar to those presented in the two-factor CFA model (exact correlations among predictors in these models can be found in Supplemental Table 5).

**Fig. 1:**
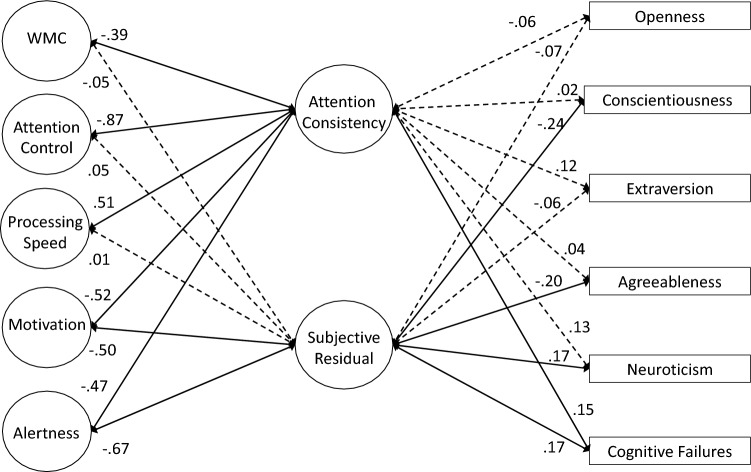
Confirmatory factor analysis of the Subjective-Residual Model. *WMC =* working memory capacity. Standardized path estimates are presented. For clarity, factor loadings are not presented here; see Table 6 for factor loadings for all models included in the primary analyses

The Subjective-Residual-Only model is presented in Fig. 1 (for clarity, Table 6 presents individual indicators and their factor loadings). Several correlations appeared consistent with our predictions. First, individual differences in WMC (− 0.39) and attention control ability (− 0.87) both were negatively correlated with general attention (in)consistency, with a stronger correlation for attention control. That is, subjects with greater WMC and attention control showed fewer attention lapses. As well, subjects with slower processing speed exhibited poorer attention consistency (0.51). Finally, subjects who reported higher motivation (− 0.52) and alertness (− 0.47) also showed fewer sustained attention failures.

In terms of dispositional constructs, only self-reported cognitive failures significantly (but weakly) correlated (0.17) with general attention (in)consistency: subjects who reported more daily failures also showed poorer attention consistency in the lab. None of the personality measures significantly correlated with the general factor. Finally, only the self-report measures (motivation, alertness, agreeableness, neuroticism, and cognitive failures) correlated with the subjective-residual factor. These correlations are unsurprising, as the subjective-residual factor likely captures variance related to self-assessments and beliefs, self-reporting biases, and socially desirable responding that might also influence responding to the contextual and dispositional self-rating measures.

**Fig. 2:**
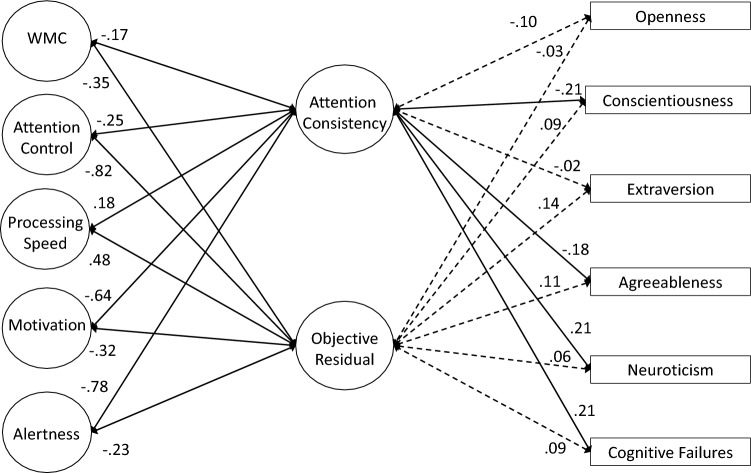
Confirmatory factor analysis of the Objective-Residual Model. *WMC* = working memory capacity. Standardized path estimates are presented. For clarity, factor loadings are not presented here; see Table 6 for factor loadings for all models included in the primary analyses

The Objective-Residual Model is displayed in Fig. 2. Here, the cognitive individual-differences variables again correlated with general attention consistency, albeit more weakly (≈ − 0.20). Self-reported alertness (− 0.78) and motivation (− 0.64) again strongly correlated with the general factor. Lastly, conscientiousness, agreeableness, neuroticism, and cognitive failures all correlated significantly (but weakly) with general ability to sustain momentary attention, most in the hypothesized directions: higher conscientiousness (− 0.21) and agreeableness (− 0.18) were related to less attention failure, while higher neuroticism (0.21) and more cognitive failures (0.21) were related to more attention failure. Correlations with the objective-residual factor were limited to our cognitive and contextual variables; none of the dispositional variables correlated significantly with the objective- residual factor.

In general, the correlations across these two separate models appear to follow the trends of the two-factor model. When general attention consistency is captured primarily by objective indicators (i.e., when the bifactor model includes a subjective-residual factor), associations with *cognitive and contextual variables* are more aligned with predictions. On the other hand, when general attention consistency is primarily captured by variance in subjective indicators (i.e., when the bifactor model includes an objective-residual factor), associations with *contextual and dispositional variables* are more in line with predictions. We will return to the complexities of interpreting the general factors from these reduced bifactor models below.^6^

### Exploratory Hierarchical Model of Attention Consistency

Because we had to conduct the bifactor models as separate reduced models (each with a different residual factor), the “general” factor did not clearly represent a general (in)ability to sustain momentary attention. This could be seen in the factor loadings of each model. Specifically, the general factor was primarily a reflection of objective measures in the subjective-residual model, and a primary reflection of subjective measures in the objective-residual model, suggesting that the reduced bifactor models did not adequately describe the data (Bornovalova et al., 2020). Thus, this imbalance in the loadings on the general factor impacted the correlations with the constructs within the nomological network.

To remedy this, we ran an *exploratory (non-preregistered)* hierarchical model to represent the general factor. Our intention was to see whether a second-order attention consistency factor that was equally loaded by first-order objective and subjective latent variables would provide some clarity about the associations between the general attention consistency factor and other constructs. We first ran a measurement model with general attention consistency as a second-order factor (with first-order factors loaded by the objective and subjective indicators), rather than a first-order general factor across the individual indicators. To identify a hierarchical model with only two first-order factors, we set the unstandardized paths of both the objective and subjective factors to 1 (Kline, 2011). This hierarchical model adequately fit the data (Table 4). Again, all individual indicators loaded onto their respective first-order latent variables (see Table 5). Additionally, the first-order latent variables were both predicted by a second-order attention consistency latent variable (Objective *β* = 0.73, Subjective *β* = 0.44). Note, however, that the residual variances for the first-order factors were large (Objective *ζ* = 0.46, Subjective *ζ* = 0.81). Despite the model fitting the data well, there was still variance that could not be explained in each first-order factor by the higher-order factor (as expected from the two-factor model showing only a moderate correlation between objective and subjective factors).

We next ran a CFA including the individual-differences constructs of interest to assess their correlations with the general attention consistency factor. When including all constructs of interest in the model, the data did not provide adequate fit (see Table 4). Inspection of the model summary indicated that the paths from attention control, alertness, and motivation to the general factor were all |*r*|> 1.0. We thus ran a second model without these nomological network constructs included. Model fit was improved and consistent with our previous models (although the TLI was still slightly below threshold). We again allowed the predictor constructs to correlate (see Supplemental Table 6).

**Fig. 3:**
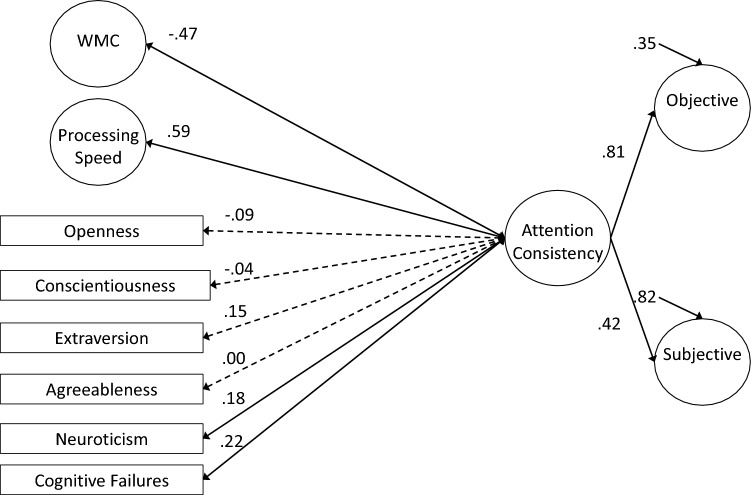
Confirmatory factor analysis of the reduced hierarchical model. *WMC* = working memory capacity. Standardized path estimates are presented. For clarity, factor loadings are not presented here; see Table 6 for factor loadings for all models included in the primary analyses

As seen in Fig. 3, WMC (− 0.47) and processing speed (0.59) both correlated with the general factor: subjects with better WMC and faster processing speed showed less sustained attention failure. Only two dispositional variables showed significant (but modest) correlations with the general (in)attention variable: individuals high in neuroticism (0.18) and those who reported more everyday attention and memory failures (0.22) had more failure of attention consistency. Given the exploratory nature of the measurement model, and the selectivity of this nomological-network model, we interpret the results with caution. Future work should consider this hierarchical structure of attention consistency as a possible model (as we will do in Study 2) and preregister analyses to investigate such associations.

### Mini-Multiverse Analyses

Extremely long RTs may reflect occasional attention lapses and will necessarily increase variability in RTs when aggregated by subject. It is also possible, however, that these RTs might sometimes result from behaviors or events completely unrelated to attention consistency (e.g., sneezing, or intentionally looking away to check the time). Thus, researchers face the challenging question of how to handle especially long RTs. Researcher degrees of freedom for treating outlying RTs are infinite, and so there is no single answer.

Choices about the cutoff values and consequences for outlying RTs (and subjects) can alter RT distributions and bias estimates of RT measures and their correlations. To investigate the robustness of our findings, then, we next conducted a preregistered mini-multiverse analysis (Steegen et al., 2017; see also Simonsohn et al., 2015). Our previous work (Welhaf et al., 2020) has found that in a large-scale dataset with hundreds of subjects and multiple tasks per construct [i.e., the Kane et al. (2016) dataset examined in present Study 2], decisions about outlying RT and subject treatments yielded negligible changes in estimates for correlations between latent variables for sustained-attention lapses and cognitive ability.

For the current study’s primary analyses above, we based RT outlier decisions on a cutoff value equal to each subject’s Median RT + 3*IQR (for each task). Here, however, for each subject, and each task, we created different datasets that either (a) retained outlying RTs, (b) censored outlying RTs to the cutoff value, or (c) cut outlying RTs. We also extended this process to univariate outlier subjects after aggregating the objective attention consistency measures (i.e., retained outlying subjects, censored outlying subjects’ scores to the cutoff value, or dropped outlying subjects’ data).

We focused our analyses on the Subjective-Residual bifactor, Objective-Residual bifactor, and hierarchical models, as these provided estimates of general attention consistency. The results are visually depicted in Supplemental Materials (Supplemental Figs. 3–7), which show that the findings were robust to varying outlier treatments. Across models, correlations with the general factor (both from the bifactor and hierarchical models) were consistent (0.03–0.10 of the primary model). For the residual factors in the bifactor models, correlations were somewhat more varied (0.06–0.10), but largely followed the pattern presented in the primary analyses. The most variability in significant correlations across these models was focused on the self-report measures, but they usually only varied in one or two iterations of the mini-multiverse aside from the neuroticism–general factor association in the hierarchical model, which was nonsignificant in about half of the iterations. Overall, the results of our mini-multiverse analyses indicate that associations between our predictor variables and attention consistency factors were robust to outlier-definition and outlier-treatment criteria.

### Discussion

Our reanalysis of Unsworth et al. (2021) provided preliminary evidence for the construct validity of objective and subjective attention consistency measures, and their covariation. First, objective measures showed within-task redundancy, with most indicators correlating above *r* = 0.70. Second, our chosen objective and subjective indicators all loaded onto their respective latent variables, which correlated moderately with each other (*r* = 0.33). This objective–subjective correlation indicated the potential for modeling a common factor, an ostensibly superior way to assess the attention consistency construct. Moreover, because bifactor models assess a common factor (attention consistency) that is orthogonal to any residual factor (objective-specific or subjective-specific), they can represent strong incremental-validity evidence for or against the claim that the covariation between objective and subjective measures provides a construct valid assessment of attention consistency.

We attempted to fit a full bifactor model to the data but were unsuccessful, but alternative (preregistered) bifactor models that separately modeled the individual residual factors fit the data. These reduced models allowed us to examine the associations of a common attention consistency factor and the residual factors with other constructs in the nomological network. Results from CFAs were somewhat in line with our predictions: individual differences in cognitive ability (e.g., WMC, attention control, and processing speed) were significantly correlated with the common factor—independently of the residual factor—such that subjects with better abilities showed better attention consistency. Individual differences in contextual factors (e.g., self-reported alertness and motivation) also correlated with the common factor—independently of the residual factor—with higher levels of each being associated with better attention consistency. Finally, dispositional characteristics provided some evidence for convergent and discriminant validity of the general attention consistency factor. Cognitive failures correlated with the common attention consistency factor in each model, indicating convergent validity. Openness and extraversion, in contrast, were uncorrelated with the general factor, suggesting discriminant validity. Correlations with other personality traits were variable across models and future work should is needed to determine how these variables fit in the nomological network of attention consistency measures.

Given that the full bifactor model (with separate residual factors for the objective and subjective indicators) did not converge, we could not adequately assess the common attention consistency factor. Each reduced bifactor model had a concerning degree of bias that muddied interpretation of the correlations with the general factor (see Bornovalova et al., 2020). Thus, to better investigate the general attention consistency factor, we conducted a series of *exploratory (non-preregistered)* analyses using a hierarchical model. Our goal was to see whether a second-order factor that had equally loading first-order objective and subjective factors could clarify any associations between a general attention consistency construct and other constructs.

The second-order factor significantly correlated with WMC and processing speed (a model with attention control and the contextual factors yielded correlations > 1.0) and with neuroticism and cognitive failures, which suggests some replicability across the models and with the literature (e.g., Kane et al., 2016; Unsworth et al., 2021). Future research should consider this hierarchical model as a worthy approach to assessing individual differences in general ability to sustain momentary attention, especially given the potential challenges of fitting bifactor models with both objective and subjective indicators residuals.

We must highlight some areas of concern for Study 1. First, despite the measurement models adequately fitting the data, none of the bifactor models that included predictor constructs fit adequately across all indices (i.e., TLI values were slightly below conventional cut-offs). Second, due to model fit issues (i.e., correlations > 1.0), motivation and alertness had to be dropped from the nomological network model. Motivation and alertness ratings were not strongly correlated with objective and subjective indicators at the task level, so the jump in latent-variable correlations was surprising. Perhaps measurement error associated with motivation and alertness is contributed to this problem, given that each was assessed using a single post-task item that was taken from the same tasks as the attention consistency measures. Future work should consider assessing these constructs with validated multi-item questionnaires (e.g., Matthews et al., 1999), or more behavioral measures of motivation and effort like cognitive discounting paradigms (Westbrook et al., 2013). All our CFA models exploring the nomological network of attention consistency should therefore be interpreted with caution pending replications. With that said, the results of Study 1 were largely robust to different outlier treatments and so we have confidence that the models can provide some preliminary evidence of the structure of attention consistency.

## Study 2

Study 1 provided preliminary evidence for the construct validity of a general attention consistency construct measured across objective and subjective indicators. Study 2 serves as a conceptual replication using an independent dataset (Kane et al., 2016) to test whether: (a) the proposed bifactor structure of attention consistency measures can be modeled, as it provides the strongest evidence for construct validity (i.e., it can demonstrate incremental validity of the general factor beyond measurement-specific factors), (b) the exploratory hierarchical model of attention consistency measures from Study 1 could be replicated, and (c) these general attention consistency factors correlate with theoretically relevant constructs.

Previous work (e.g., Unsworth, 2015) analyzed RT CoV in some attention control tasks that elicited conflict on some trials (i.e., incongruent Stroop trials). Doing so creates a potential confound of the experimental effect (i.e., Stroop interference) and CoV measurement. That is, someone with relatively slow RTs on incongruent trials will show both a larger Stroop RT effect and greater RT variability across trials. To avoid such confounds, Kane et al. (2016) reported models using CoV from only non-conflict trials in their attention control tasks. If high CoV and other objective measures reflect momentary lapses of attention, then these failures should be captured on trials that do not contain conflict.

Study 2 assessed, in addition to WMC, a new construct—positive schizotypy—to further investigate convergent and discriminant validity. Prior work has demonstrated that positive schizotypy, reflecting the proneness to have unusual beliefs and perceptual experiences (and a risk factor for schizophrenia and related disorders), is related to both objective and subjective indicators of attention consistency. Specifically, subjects with higher positive schizotypy scores (from self-report questionnaires) show more variable RTs in basic attention tasks (Kane et al., 2016; Schmidt-Hansen & Honey, 2009), as well as higher TUT rates (Kane et al., 2016), than do those with lower scores. Thus, positive schizotypy should be negatively correlated with an assessment of general ability to momentarily sustain attention.

### Methods

Below we describe the general procedure and materials from Kane et al. (2016). We provide detailed descriptions of the tasks and measures selected for the current study in their respective sections.

#### Subjects

Kane et al. (2016) enrolled 545 undergraduates into their study from the University of North Carolina at Greensboro, a comprehensive state university with a diverse undergraduate population. Of these, 541 completed the first of three 2-h sessions, 492 completed the second, and 472 completed all three. Full-information maximum likelihood estimation was used for missing data (see Kane et al., 2016, for details and sample demographics).

#### Materials

**Objective Attention Consistency Measures.** As in Study 1, for each objective indicator task, we assessed multiple dependent measures that theoretically should reflect variation in attention consistency. We focus the present analyses on tasks where RT was the primary outcome in Kane et al. (2016). Our procedure for selecting a primary indicator for each task was identical to that for Study 1. Again, we list the possible dependent measures for each task with the preregistered, a priori measure listed first. For many of the tasks in Study 2, however, there was no measure that has traditionally been used to reflect attention consistency (aside from the SART), so we tried to balance RTsd, *τ*, and slowest 20% of trials across the tasks such that each was the primary indicator for at least one of the “attention restraint” tasks (i.e., SART and Stroop-like tasks), and one was the primary measure among the “attention constraint” tasks (i.e., flanker tasks).

**Semantic SART.** In this go/no-go task, subjects pressed the space bar for words from one category (*animals*; 89% of trials) but withheld responding to words from another category (*vegetables*; 11% of trials). Stimuli were presented for 300 ms followed by a 1500 ms mask. There were 675 trials, 75 of which were no-go targets. The potential dependent variables derived from this task were intra-individual RTsd, intra-individual RTmad, omission errors, mean RT of the slowest 20% of trials, mean RT of the fastest 20% of trials, the *τ* estimate from an ex-Gaussian model, and RMSSD, all for correct “go” trials.

**Number Stroop.** Subjects reported the number of digits presented on each trial while ignoring the digits’ identity. Each trial presented 2–4 identical digits in a row and subjects responded with one of three labeled keys to indicate the number of digits on-screen. There were 300 total trials: 240 were congruent (e.g., “*333*”) and 60 were incongruent (e.g., “*222*”). The potential dependent variables derived from this task were the *τ* estimate from an ex-Gaussian model, RTsd, RTmad, and mean RT of the slowest 20% of trials, all for correct congruent trials.

**Spatial Stroop.** Subjects reported the relative position of a word to an asterisk (left, right, above, below), with the word and asterisk both presented to the left or right, or above or below, fixation; subjects ignored both the identity of the word (“*LEFT*,” “*RIGHT*,” “*ABOVE*,” “*BELOW”*) and absolute location of the word and asterisk on screen. Subjects responded to the relative position of the word to the asterisk by pressing the corresponding arrow on the numeric keypad arrow keys. Subjects completed a total of 120 trials: 60 presenting words congruent for absolute and relative location, 30 presenting words congruent for absolute location but incongruent for relative location, and 30 presenting words incongruent both for absolute and relative location. Here, the potential dependent variables derived from this task were mean RT of the slowest 20% of trials, RTsd, RTmad, and the *τ* estimate from an ex-Gaussian model, all for correct responses to trials where words were congruent for both absolute and relative position.

**Arrow Flanker.** Subjects reported the direction of a centrally presented arrow (“<” vs. “>”) via keypress, with the arrow flanked horizontally by 4 distractors. Subjects completed two blocks of 96 trials: 24 neutral trials (target arrow presented amid dots), 24 congruent trials (all arrows pointing the same direction), 24 stimulus–response incongruent trials (central arrow pointing opposite direction of flankers), and 24 stimulus-stimulus incongruent trials (central arrow presented amid upward-pointing arrows). Here, the potential dependent variables derived from this task were mean RT of the slowest 20% of trials, RTsd, RTmad, and the *τ* estimate from an ex-Gaussian model, all for correct responses to both neutral and congruent trials.

**Letter Flanker.** Subjects reported whether a centrally presented “F” appeared normally or backwards via keypress, with that letter flanker horizontally by 6 distractors. Subjects completed 144 trials: 24 neutral trials (normal or backwards F presented amid dots), 48 congruent trials (target and distractor Fs all facing the same direction), 24 stimulus–response incongruent trials (target facing opposite direction of distractors), and 24 stimulus-stimulus incongruent trials (target presented amid right- and left- facing Es and Ts tilted at 90 and 270 degrees). Here, the potential dependent variables derived from this task were RTsd, RTmad, mean RT of the slowest 20% of trials, and the *τ* estimate from an ex-Gaussian model, all for correct responses to neutral and congruent trials.

**Circle Flanker.** Subjects reported whether a target letter was an X or N, via keypress, with the target flanked by two distractors. Targets appeared in one of eight possible locations in a circle, with distractors appearing on either side of the target; all other locations were occupied by colons. Subjects completed 160 trials: 80 neutral trials (target letter surrounded by colons) and 80 conflict trials (target flanked by two different distractors from the set H, K, M, V, Y, Z). Here, the potential dependent variables derived from this task were the *τ* estimate from an ex-Gaussian model, RTsd, RTmad, and mean RT of the slowest 20% of trials, all using correct responses to neutral trials.

**Subjective Attention Consistency Measures.** Thought probes were randomly presented in 5 tasks (45 in SART, 20 in Number Stroop, 20 in Arrow Flanker, 12 in Letter Flanker, and 12 in an otherwise-unanalyzed 2-back task). Each probe presented subjects with eight categories of thoughts they might have just experienced. Subjects selected their options by pressing the number on the keyboard that most closely matched the content of their immediately preceding thoughts. The options were: (1) “The task” (thoughts about the stimuli or responses); (2) “Task experience/performance” (thoughts about how one was performing on the task); (3) “Everyday things” (thoughts about normal life concerns, goals, and activities); (4) “Current state of being” (thoughts about one’s physical, cognitive, or emotional states); (5) “Personal worries” (thoughts about current worries); (6) “Daydreams” (fantastical, unrealistic thoughts); (7) “External environment” (thoughts about task-unrelated things or events in the immediate environment); (8) “Other.” TUTs were assessed as the proportion of responses with options 3–8, as in Kane et al. (2016).

**WMC tasks.** Subjects completed four complex span tasks and two updating tasks. As in Study 1, for each complex span task, subjects completed three practice stages: the first provided practice in memorizing small sets of the memoranda (e.g., letters, grid, or arrow locations); the second practice was for processing-only (e.g., math equations, symmetry decisions, sentence comprehension, letter direction). RTs were recorded during this processing-only practice for each subject. During the real trials, if a processing decision was not made within 2.5 SDs of the processing-only mean, that trial was counted as a processing error; the third practice consisted of both the memory and processing task combined (as in the real trials).

**Operation Span.** Same as Study 1. Here, however, the set sizes of 3–7 were presented three times in a random order rather than twice (max score of 75).

**Reading Span.** Same as Study 1. Here, however, the set sizes of 2–6 were presented three times in a random order rather than twice (max score of 60).

**Symmetry Span.** Same as Study 1. Here, however, the set sizes of 2–5 were presented three times in a random order rather than twice (max score of 42).

**Rotation Span.** Subjects were presented with random sequences of large and small arrows to remember, radiating from a center location in one of 8 possible directions. Between presentation of each arrow, a rotated letter (F, G, J, or R) was presented facing its normal direction or mirror-reversed (50% of the time) and subjects had to verify its direction. At the end of the set, subjects recalled the arrows from the set by clicking the location onscreen in the presented serial order. Subjects were granted credit only if the arrow was recalled in the correct serial position. Set sizes ranged from 2 to 5 items; each set size was presented three times (for a max score of 42). Higher scores reflected better recall.

**Running Span.** Subjects were presented with a sequence of letters and were asked to recall only the final 3–7 letters from the trial. Trials were unpredictably 0, 1, or 2 items longer than the set size (e.g., set size 5 had list lengths of 5, 6, and 7 items in the task). Each trial started with a number to indicate the set size (i.e., the number of items to be recalled at the end of the list). At the end of the list, all 12 possible letters appeared on screen along with the corresponding set size and subjects selected via mouse-click the appropriate letters from the set (in serial position). Subjects completed 15 total trials. Credit was granted for items that were recalled in the correct serial position (for a max of 75). Higher scores reflected better recall.

**Updating Counters.** Subjects recalled the numerical values presented in boxes, some of which were updated from their original values. Each trial began with 3–5 boxes presented horizontally on-screen. There were three phases for each trial: (1) the learning phase, where a digit (1 thru 9) was presented in a random order in each box; (2) the updating phase, where 2–6 of the box values were changed by presenting a simple addition or subtraction (e.g., + 4; − 1; updates ranged from − 7 to + 7). Updates appeared randomly and some boxes could have been updated multiple times, or not at all; (3) the recall phase, where subjects were tasked with recalling the final updated value for each box (cued in a random order). Set sizes of 3–5 boxes were crossed with the number of updates (2–6) yielding a total of 15 trials. Credit was granted for correct answers and the score was proportion correct (out of 60). Higher scores reflected better recall.

**Positive Schizotypy.** Positive schizotypy was assessed using two of the Wisconsin Schizotypy Scales (WSS; Eckblad & Chapman, 1983; Chapman et al. 1978)—the Perceptual Aberration scale and Magical Ideation scale—and the Referential Thinking subscale of the Schizotypal Personality Questionnaire (Raine, 1991). Subjects saw each item on-screen individually and responded via mouse-click if the item was true for them (scored as 1) or false (scored as 0). After appropriate reverse-scoring, items were summed for each scale where higher scores indicated more endorsement of the schizotypic belief or experience. [Note that in Kane et al. (2016), Social Anhedonia item parcels were also included (as cross-loadings) in the positive schizotypy factor; however, their factor loadings were weak (< 0.30) and, as expected, they loaded more strongly on the negative schizotypy factor, and so they will not be included for the current analyses.]

### Results

We again report our preregistered analyses and results and note explicitly where we deviated from the preregistered plan. Data and Rmarkdown files for all analyses are available on the Open Science Framework (https://osf.io/xeu63/).

#### Data Analysis Exclusions

Prior to calculating any of the primary DVs for the current study, we calculated “go” trial accuracy for the SART and identified 6 subjects who had “go” trial accuracy < 70%. Consistent with Study 1, *we deviated from our preregistration* and excluded SART data from these subjects, as such low accuracy might indicate a failure to understand or comply with task instructions, rather than failures of sustained attention.

#### RT Cleaning Procedures

We implemented the same preregistered multi-step procedure for trial-level cleaning as Study 1. First, we identified and removed RTs for error and post-error trials (and post-probe trials in tasks that included thought probes). Next, we removed RTs for trials that were likely anticipations (i.e., RTs < 200 ms). From the remaining trials, we next calculated for each subject, in each task, a value equal to their Median RT + 3*IQR. Any trials outside of this value were replaced with this value. Appendix B reports the relevant descriptive information for the trial-level cleaning (e.g., mean number of trials outlying trials replaced per task). Finally, we calculated the number of usable trials each subject had following our RT cleaning protocol. As preregistered, we dropped task data from subjects who did not have at least 40 trials and thus could not reliably contribute to our primary measures of interest. This resulted in dropping data from 2 subjects in the Spatial Stroop task and 6 in the Arrow flanker task.

#### Selection of Objective Indicators

We followed the same preregistered procedures for selecting and objective attention consistency indicators as in Study 1. Supplemental Table 10 presents the descriptive statistics for each possible DV, for each task, as well as the number of subjects whose data were censored for each potential measure.

No variables were dropped for problematic distributions (skew > 3.0 or kurtosis > 10.0). We next examined split-half reliability to remove unreliable indicators (i.e., < 0.50). No variables were removed for poor reliability. As in Study 1, we next examined within-task correlations to see whether any combination of measures captured different degrees of attention failure than the a priori measure for each task. As seen in Supplemental Table 11, many of the proposed measures were redundant (*r*s > 0.70) within each task, for the Number Stroop, Spatial Stroop, Arrow flanker, Letter flanker, and Circle flanker tasks. Because of this, we selected only the preregistered a priori measure for each of these tasks. However, as in Study 1, we found evidence in the SART that some potential indicators were not redundant. Specifically, RTsd (the a priori measure) and Omissions were correlated at less than 0.70 (*r* = 0.51), so we retained both.

#### Multivariate Outliers

As preregistered, once we established primary indicators, we again checked for multivariate outliers in the final dataset using the *Routliers* package (Leys et al., 2013) to calculate Mahalanobis distance for each observation in the dataset. This analysis indicated there were 10 multivariate outliers in the dataset (~ 2% of the subjects). These subjects were removed case-wise before any structural modeling was conducted.

#### Descriptive Statistics and Correlations for Final Dataset

Table 8 provides the descriptive statistics for the final dataset of Study 2. Kane et al. (2016) reported adequate to strong reliabilities for the measures we used here: WMC range = 0.54–0.85, Positive Schizotypy range = 0.70–0.85, TUT rates range = 0.78–0.93). For the new objective attention consistency measures used in the final analyses, reliabilities were strong (> 0.90). Table 9 provides the bivariate correlations of all measures of interest. Consistent with Kane et al. (2016), measures from the same proposed construct (e.g., WMC, Positive Schizotypy, TUT rates) all correlated more strongly with each other than with measures of other constructs. Importantly, and consistent with Study 1, our newly selected objective attention consistency indicators also correlated moderately with each other (median |*r|*= 0.30) suggesting that subjects who showed variable responding in one task also tended to do so in other tasks.

**Table 8:** Descriptive statistics for Study 2 measures

Construct/measure	Mean	SD	Min	Max	Skew	Kurtosis	*N*
**Objective Attention Consistency**							
SART RTsd	159.30	58.75	36.64	361.34	1.20	1.73	510
SART Omissions	22.96	24.86	0.00	94.00	1.43	1.14	510
Number Stroop *τ*	91.14	39.74	14.96	220.92	1.31	1.65	458
Spatial Stroop Bin 5	974.20	289.51	511.50	1857.01	1.34	1.55	446
Arrow Flanker Bin 5	630.31	111.51	426.90	1011.21	0.92	0.67	464
Letter Flanker RTsd	121.79	54.88	40.56	307.44	1.21	1.37	452
Circle Flanker *τ*	113.31	58.01	0.00	284.81	1.16	1.37	458
**Subjective Attention Consistency**							
SART TUTs	0.51	0.24	0.00	1.00	− 0.04	− 0.81	510
Number Stroop TUTs	0.43	0.29	0.00	1.00	0.38	− 0.90	458
Arrow Flanker TUTs	0.49	0.30	0.00	1.00	0.11	− 1.07	464
Letter Flanker TUTs	0.58	0.26	0.00	1.00	− 0.47	− 0.54	452
N-Back TUTs	0.42	0.31	0.00	1.00	0.31	− 1.09	451
**Working Memory Capacity**							
OPERSPAN	0.00	1.00	− 3.54	1.70	− 0.75	0.31	465
READSPAN	0.00	1.00	− 2.77	2.27	− 0.23	− 0.44	413
SYMSPAN	0.01	0.99	− 3.22	2.01	− 0.37	− 0.17	457
ROTSPAN	0.02	0.97	− 3.19	2.10	− 0.48	− 0.09	377
RUNNSPAN	0.00	0.99	− 2.72	2.84	0.22	− 0.10	452
COUNTERS	− 0.01	0.99	− 2.04	3.24	0.55	0.17	470
**Positive Schizotypy**							
PERCABER	6.37	4.96	0.00	31.00	1.55	3.43	523
MAGIDEA	11.43	5.57	0.00	28.00	0.24	− 0.52	523
REFTHINK	3.35	2.06	0.00	7.00	0.09	− 1.03	469

*Working Memory Capacity* scores are *z* scores, *SART =* sustained attention to response task, *TUTs* = rate of task-unrelated thoughts in specified task, *OPERSPAN* = operation span, *READSPAN* = reading span, *SYMSPAN =* symmetry span, *ROTSPAN =* rotation span, *RUNNSPAN* = running span, *COUNTERS* = updating counters task, *PERCABER* = perceptual aberration total score, *MAGIDEA* = magical ideation total score, *REFTHINK= * referential thinking score, *Bin 5* = mean RT of slowest 20% of correct trials, *RTsd* = intra-individual RT variability, *Bin 1* = mean RT of fastest 20% of correct trials

**Table 9:** Zero-order correlations of variables for structural models in Study 2

Variable	1	2	3	4	5	6	7	8	9	10	11	12	13	14	15	16
1. SART RTsd	1															
2. SART Omissions	0.51	1														
3. Number Stroop *τ*	0.39	0.31	1													
4. Spatial Stroop Bin 5	0.20	0.14	0.28	1												
5. Arrow Flanker Bin 5	0.26	0.15	0.34	0.41	1											
6. Letter Flanker RTsd	0.37	0.37	0.33	0.29	0.41	1										
7. Circle Flanker *τ*	0.29	0.26	0.49	0.30	0.44	0.37	1									
8. SART TUTs	0.24	0.24	0.18	0.09	0.02	0.22	0.14	1								
9. Number Stroop TUTs	0.13	0.18	0.29	0.06	0.15	0.15	0.26	0.44	1							
10. Arrow Flanker TUTs	0.12	0.15	0.18	0.05	0.15	0.10	0.21	0.42	0.70	1						
11. Letter Flanker TUTs	0.11	0.14	0.19	0.06	0.08	0.28	0.10	0.53	0.37	0.37	1					
12. N-Back TUTs	0.22	0.20	0.19	0.07	0.12	0.18	0.13	0.38	0.47	0.40	0.32	1				
13. OPERSPAN	− 0.15	− 0.10	− 0.08	− 0.09	− 0.11	− 0.05	− 0.04	− 0.01	− 0.05	0.00	0.09	− 0.07	1			
14. READSPAN	− 0.10	− 0.18	− 0.04	− 0.04	− 0.05	− 0.09	− 0.06	− 0.13	− 0.13	− 0.10	0.00	− 0.10	0.57	1		
15. SYMSPAN	− 0.18	− 0.14	− 0.18	− 0.13	− 0.16	− 0.16	− 0.10	− 0.06	− 0.04	− 0.04	− 0.08	− 0.06	0.39	0.39	1	
16. ROTSPAN	− 0.10	− 0.01	− 0.07	− 0.10	− 0.15	− 0.12	− 0.10	0.00	− 0.03	− 0.01	0.02	− 0.14	0.43	0.32	0.54	1
17. RUNNSPAN	− 0.24	− 0.22	− 0.12	− 0.13	− 0.17	− 0.11	− 0.11	− 0.05	− 0.13	− 0.12	0.03	− 0.21	0.45	0.37	0.26	0.16
18. COUNTERS	− 0.27	− 0.19	− 0.22	− 0.17	− 0.25	− 0.21	− 0.25	− 0.06	− 0.04	− 0.04	− 0.02	− 0.13	0.36	0.22	0.38	0.29
19. PERCABER1	0.02	0.07	0.04	0.04	0.08	0.10	0.12	0.05	0.15	0.15	0.11	0.14	− 0.03	− 0.03	0.04	0.03
20. PERCABER2	0.06	0.10	0.07	0.03	0.06	0.02	0.05	0.00	0.11	0.11	0.10	0.10	0.05	0.05	0.09	0.08
21. PERCABER3	0.13	0.14	0.07	0.10	0.16	0.12	0.09	0.08	0.14	0.17	0.15	0.14	− 0.07	− 0.07	0.02	0.04
22. MAGIDEA1	0.09	0.14	0.08	0.06	0.05	0.11	0.08	0.07	0.12	0.13	0.11	0.06	0.00	− 0.04	0.09	0.05
23. MAGIDEA2	0.03	0.10	0.07	0.00	0.02	0.07	0.06	0.02	0.10	0.11	0.10	0.11	0.06	0.00	0.10	0.07
24. MAGIDEA3	0.01	0.09	0.02	0.02	− 0.02	0.09	− 0.02	0.06	0.10	0.13	0.12	0.12	− 0.04	− 0.07	0.05	0.04
25. REFTHINK	0.07	0.15	0.14	0.11	0.03	0.07	0.11	0.13	0.16	0.18	0.09	0.09	− 0.06	− 0.10	− 0.01	0.03

Variable	17	18	19	20	21	22	23	24
17. RUNNSPAN	1							
18. COUNTERS	0.39	1						
19. PERCABER1	− 0.10	− 0.06	1					
20. PERCABER2	− 0.12	− 0.04	0.67	1				
21. PERCABER3	− 0.19	− 0.08	0.64	0.64	1			
22. MAGIDEA1	− 0.10	− 0.09	0.51	0.49	0.53	1		
23. MAGIDEA2	− 0.05	0.00	0.50	0.48	0.55	0.63	1	
24. MAGIDEA3	− 0.11	− 0.06	0.45	0.42	0.46	0.56	0.63	1
25. REFTHINK	− 0.14	− 0.12	0.35	0.36	0.41	0.56	0.54	0.46

*SART* = sustained attention to response task, *TUTs* = rate of task-unrelated thoughts in specified task, *OPERSPAN* = operation span, = *READSPAN* reading span, = *SYMSPAN* symmetry span, *ROTSPAN* = rotation span, *RUNNSPAN* = running span, *COUNTERS* = updating counters task, *PERCABER =* perceptual aberration total score, *MAGIDEA =* magical ideation total score, *REFTHINK= * referential thinking score, *Bin 5 =* mean RT of slowest 20% of correct trials, *RTsd =* intra-individual RT variability, *Bin 1* = mean RT of fastest 20% of correct trials

#### Measurement Models of Attention Consistency

Our first set of preregistered analyses conceptually replicated the latent variable correlation between performance and self-report indicators of attention consistency from Study 1. We tested a 2-factor model with latent variables for objective (i.e., RT variability) and subjective (i.e., TUT reports) measures; these latent variables were allowed to correlate. Consistent with Study 1 (*but not preregistered for either study*), we included within-task residual correlations between the TUT rate and performance indicator (e.g., Number Stroop *τ* with Number Stroop TUTs). We retained these residual correlations for all subsequent models. As seen in Table 10, the model fit the data adequately. Moreover, the latent variables for objective and subjective measures again correlated moderately, as in Study 1 (*r* = 0.38, here, and *r* = 0.32 in Study 1; see Table 11 for factor loadings).^7^ This *moderate* correlation suggests that these two types of attention consistency measures are not redundant. Modeling their shared variance should provide a more construct valid measure of attention consistency, free from measurement error specific to either indicator type.

**Table 10:** Fit statistics for latent variable models for Study 2

Model	*χ*^2^ (*df*)	*χ*^2^/df	CFI	TLI	RMSEA [90% CI]	SRMR
**Measurement Models**						
2-factor	109.213 (45)	2.43	0.959	0.939	0.052 [0.040–0.065]	0.042
Bifactor	66.444 (34)	1.95	0.979	0.959	0.043 [0.027–0.058]	0.028
Hierarchical	109.213 (45)	2.43	0.959	0.939	0.052 [0.040–0.065]	0.042
**Confirmatory Factor Analysis**						
2-factor	453.284 (253)	1.79	0.951	0.941	0.039 [0.033–0.044]	0.053
Bifactor	403.998 (240)	1.68	0.959	0.949	0.036 [0.030–0.042]	0.049
Hierarchical	460.799 (255)	1.81	0.949	0.940	0.039 [0.033–0.045]	0.056
Limited AC bifactor	417.746 (242)	1.73	0.955	0.945	0.037 [0.031–0.042]	0.052
Limited AC hierarchical	454.008 (256)	1.77	0.949	0.941	0.038 [0.032–0.044]	0.056

*AC* = attention control

**Table 11:** Standardized factor loadings (and standard errors) for latent variable models for Study 2

Construct and Measure	Model Names					
	Two Factor Measurement	Bifactor Measurement	Hierarchical Measurement	Two Factor CFA	Bifactor CFA	Hierarchical CFA
**Working Memory Capacity**						
OPERSPAN				0.64 (0.04)	0.64 (0.04)	0.65 (0.04)
READSPAN				0.52 (0.05)	0.52 (0.05)	0.53 (0.05)
SYMSPAN				0.59 (0.05)	0.59 (0.05)	0.59 (0.05)
ROTSPAN				0.49 (0.06)	0.48 (0.06)	0.49 (0.05)
RUNSPAN				0.60 (0.04)	0.60 (0.04)	0.60 (0.04)
COUNTERS				0.62 (0.04)	0.62 (0.04)	0.61 (0.04)
**Positive Schizotypy**						
PERCABER1				0.60 (0.03)	0.60 (0.03)	0.60 (0.03)
PERCABER2				0.58 (0.03)	0.58 (0.03)	0.58 (0.03)
PERCABER3				0.64 (0.03)	0.64 (0.03)	0.64 (0.03)
MAGIDEA1				0.84 (0.03)	0.85 (0.03)	0.84 (0.03)
MAGIDEA2				0.83 (0.03)	0.83 (0.03)	0.83 (0.03)
MAGIDEA3				0.72 (0.04)	0.72 (0.04)	0.72 (0.04)
REFTHINK				0.66 (0.03)	0.66 (0.03)	0.66 (0.03)
**Attention Consistency**						
SART RTsd		0.54 (0.06)			0.61 (0.07)	
SART Omissions		0.52 (0.06)			0.62 (0.08)	
Number Stroop *τ*		0.69 (0.07)			0.62 (0.07)	
Spatial Stroop Bin 5		0.32 (0.10)			0.30 (0.09)	
Arrow Flanker Bin 5		0.35 (0.13)			0.36 (0.13)	
Letter Flanker RTsd		0.55 (0.07)			0.55 (0.07)	
Circle Flanker *τ*		0.59 (0.08)			0.53 (0.08)	
SART TUTs		0.28 (0.06)			0.28 (0.07)	
Number Stroop TUTs		0.34 (0.06)			0.32 (0.06)	
Arrow Flanker TUTs		0.27 (0.06)			0.26 (0.06)	
Letter Flanker TUTs		0.25 (0.06)			0.24 (0.06)	
N-Back TUTs		0.30 (0.06)			0.33 (0.06)	
**Objective**/**Objective**^**resid**^						
SART RTsd	0.52 (0.04)	0.14 (0.10)	0.52 (0.04)	0.54 (0.04)	0.10 (0.13)	0.52 (0.04)
SART Omissions	0.44 (0.04)	0.02 (0.10)	0.44 (0.04)	0.46 (0.04)	− 0.05 (0.14)	0.45 (0.04)
Number Stroop *τ*	0.67 (0.04)	0.17 (0.13)	0.67 (0.04)	0.67 (0.04)	0.24 (0.12)	0.66 (0.04)
Spatial Stroop Bin 5	0.49 (0.04)	0.42 (0.11)	0.49 (0.04)	0.50 (0.04)	0.46 (0.08)	0.50 (0.04)
Arrow Flanker Bin 5	0.64 (0.04)	0.78 (0.14)	0.64 (0.04)	0.64 (0.04)	0.70 (0.12)	0.63 (0.04)
Letter Flanker RTsd	0.64 (0.04)	0.31 (0.15)	0.64 (0.04)	0.63 (0.04)	0.31 (0.10)	0.63 (0.04)
Circle Flanker *τ*	0.69 (0.03)	0.32 (0.12)	0.69 (0.03)	0.68 (0.03)	0.38 (0.11)	0.68 (0.03)
**S﻿ubjective**/**Subjective**^**resid**^						
SART TUTs	0.60 (0.04)	0.55 (0.05)	0.60 (0.04)	0.60 (0.04)	0.55 (0.06)	0.62 (0.04)
Number Stroop TUTS	0.74 (0.04)	0.66 (0.06)	0.74 (0.04)	0.74 (0.04)	0.67 (0.06)	0.74 (0.04)
Arrow Flanker TUTS	0.70 (0.04)	0.65 (0.06)	0.70 (0.04)	0.70 (0.04)	0.66 (0.06)	0.69 (0.04)
Letter Flanker TUTS	0.51 (0.05)	0.45 (0.06)	0.51 (0.05)	0.51 (0.05)	0.45 (0.06)	0.51 (0.05)
N-Back TUTS	0.62 (0.04)	0.54 (0.05)	0.62 (0.04)	0.62 (0.04)	0.52 (0.06)	0.63 (0.04)

*OPERSPAN* = operation span, *READSPAN* = reading span, *SYMMSPAN* = symmetry span, *ROTASPAN* = rotation span, *RUNNSPAN* = running span, *COUNTERS* = updating counters, *SART RTsd =* intrasubject standard deviation in RT from SART, *Letter Flanker RTsd =* intrasubject standard deviation in RT from Letter Flanker, *PERCABER1* = perceptual aberration scale (parcel 1), *PERCABER2* = perceptual aberration scale (parcel 2), *PERCABER3* = perceptual aberration scale (parcel 3), *MAGIDEA1* = magical ideation scale (parcel 1), *MAGIDEA2* = magical ideation scale (parcel 2), *MAGIDEA3 =* magical ideation scale (parcel 3), *REFTHINK =* referential thinking subscale from the Schizotypal Personality Questionnaire (SPQ), *SART* = sustained attention to response task, *TUTs* = TUT rate from task

O﻿ur next preregistered measurement model was a bifactor model, which attempted to model common attention consistency variance across all the objective and subjective indicators, as well as residual variance unique to each indicator type. Unlike Study 1, this full bifactor model adequately fit the data (see Table 10). All indicators loaded significantly onto the general factor (although the loadings were mostly stronger for the objective than subjective indicators) and there was also enough shared variance among the measures to model both an objective- and subjective-residual factor (see Table 11 for factor loadings).

#### Preregistered Confirmatory Factor Analyses of Individual Differences in Attention Consistency

Our next set of preregistered analyses assessed the correlations between our nomological network constructs with our different attention consistency models. Although our focus was the bifactor model, we first present the correlations between our predictors and the two-factor attention (in)consistency model. A model with latent variables for WMC and positive schizotypy adequately fit the data (Table 10) and all indicators loaded onto their respective factors (Table 11). WMC correlated negatively with the objective (*r* = − 0.42) and subjective factors (*r* = − 0.19): subjects with higher WMC exhibited fewer performance lapses and TUT reports. In contrast, positive schizotypy correlated positively with both the objective (*r* = 0.16) and subjective factors (*r* = 0.21): individuals who endorsed more positive schizotypy experiences exhibited more performance lapses and more TUT reports. WMC did not correlate with positive schizotypy (*r* = − 0.04).

**Fig. 4:**
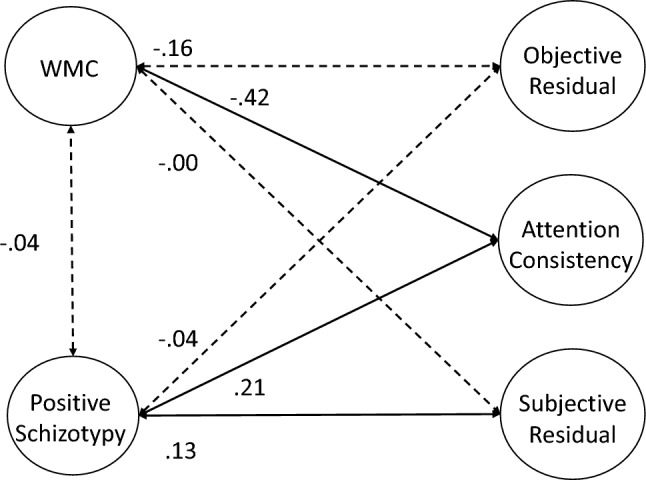
Confirmatory factor analysis of the bifactor model of attention consistency (failures) for Study 2. *WMC* = working memory capacity. Standardized path estimates are presented. For clarity, factor loadings are not presented here; see Table 11 for factor loadings for all models included in the primary analyses

To assess whether our predictor constructs correlated with a general factor of attention (in)consistency, we next ran a CFA with the bifactor model. As seen in Fig. 4, both WMC and positive schizotypy significantly correlated with the general attention consistency factor in predicted directions: subjects with higher WMC and those who reported lower positive schizotypy ratings had fewer failures to sustain attention consistency, independent of any associations with the object-residual or subjective-residual factors; that is, WMC and positive schizotypy were associated with the variance that was common to both objective and subjective measures, above and beyond their potential associations with variance specific to objective or subjective measures (i.e., the general factor demonstrated incremental validity). Neither WMC nor positive schizotypy was correlated significantly with the objective-residual factor. However, there was a weak but significant positive association between positive schizotypy and the subjective-residual factor (WMC did not correlate with this factor).

#### Exploratory Hierarchical Model of Attention Consistency

As a final exploratory model, following from Study 1, we modeled attention consistency as a second-order factor above the first-order objective- and subjective-indicator factors. We again set the unstandardized paths of the objective and subjective factors to 1 to yield an identified model. The measurement model showed acceptable fit (see Table 10), with both the objective (*β* = 0.68) and subjective (*β* = 0.57) factors loading significantly onto the second-order attention consistency factor. Again, we note that the variances on the first-order factors were large, suggesting there was still unexplained variance in the model (Objective *ζ* = 0.57, Subjective *ζ *= 0.67), as expected given the moderate correlation between objective and subjective factors.

**Fig. 5:**
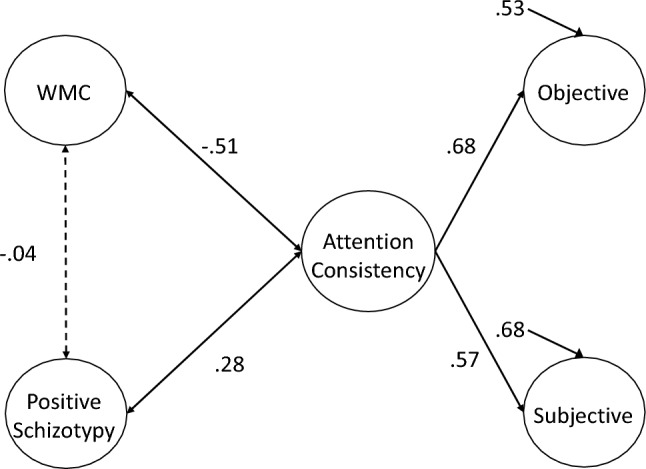
Confirmatory factor analysis of the hierarchical model of attention consistency (failures) for Study 2. *WMC* = working memory capacity. Standardized path estimates are presented. For clarity, factor loadings are not presented here; see Table 11 for factor loadings for all models included in the primary analyses

We next ran a CFA including WMC and positive schizotypy and the model adequately fit the data (Table 10). As seen in Fig. 5, both WMC and positive schizotypy significantly correlated with the second-order attention (in)consistency factor, with similar magnitudes to those with the general factor from the bifactor model, although they were a bit larger here (the WMC path was also of nearly identical magnitude here to that from Study 1 [− 0.47]): higher WMC was again related to fewer attention failures whereas higher positive schizotypy scores were again related to more attention failures, with attention (in)consistency reflecting the variance shared between objective-specific and subjective-specific factors.

#### Exploratory CFAs Including a Narrow “Attention Control” Factor

A limitation of our preregistered structural models is that they left us unable to address questions about the association between general attention consistency and other factors of attention control. Recall that in the Study 1 hierarchical model, attention control correlated > 1.0 with the attention consistency factor, which led us to drop that factor (along with the motivation and alertness factors) in our reduced model. Although the original study on which Study 2 is based (Kane et al., 2016) included separate latent factors for “attention restraint” (response-conflict-type tasks) and “attention constraint” (flanker distractor-control tasks), here we included indicators from most of these tasks (using their congruent and or neutral-baseline conditions) to model our attention consistency performance factor.

*As an exploratory, non-preregistered approach* to the question, however, here we modeled an attention control factor using the two antisaccade tasks and the SART dʹ measure (reflecting part of the “restraint” factor from Kane et al., 2016), and we removed all SART indicators (RTsd, omissions, TUT rate) from the attention consistency factors. Our measurements of attention control and attention consistency constructs were thus independent. Otherwise, the models matched those represented in Fig. 5, including WMC, positive schizotypy, and either the bifactor or hierarchical model of attention consistency.

Both the bifactor and hierarchical models again fit the data (see Table 10 for fit statistics and Table 12 for factor loadings). In the bifactor model, attention control (failure) was moderately correlated with the general attention consistency factor (*r* = 0.31), the objective-residual factor (*r* = 0.38), and, in contrast to WMC, also with the subjective-residual factor (*r* = 0.22). In the hierarchical model, attention control (failure) was strongly correlated with the second-order attention consistency factor (*r* = 0.71). Thus, in both cases, subjects with poorer attention control, assessed by conflict tasks, also showed poorer attention consistency. Unlike Study 1, then, here we were provisionally able to dissociate attention consistency from attention control, which suggests these may be distinct forms of general executive attentional ability.

**Table 12:** Standardized factor loadings (and standard errors) for limited attention control latent variable models for Study 2

Construct and Measure	Model Names	
	Bifactor CFA	Hierarchical CFA
**Working Memory Capacity**		
OPERSPAN	0.62 (0.04)	0.62 (0.04)
READSPAN	0.50 (0.05)	0.50 (0.05)
SYMSPAN	0.62 (0.05)	0.62 (0.05)
ROTSPAN	0.52 (0.06)	0.52 (0.06)
RUNSPAN	0.58 (0.04)	0.58 (0.04)
COUNTERS	0.63 (0.04)	0.62 (0.04)
**Positive Schizotypy**		
PERCABER1	0.60 (0.03)	0.60 (0.03)
PERCABER2	0.58 (0.03)	0.58 (0.03)
PERCABER3	0.64 (0.03)	0.64 (0.03)
MAGIDEA1	0.84 (0.03)	0.85 (0.03)
MAGIDEA2	0.83 (0.03)	0.83 (0.03)
MAGIDEA3	0.72 (0.04)	0.72 (0.04)
REFTHINK	0.66 (0.03)	0.66 (0.03)
**Attention Control**		
SART *d*′	− 0.45 (0.04)	− 0.46 (0.04)
Antisaccade Letters	0.77 (0.03)	0.77 (0.03)
Antisaccade Arrows	0.76 (0.04)	0.76 (0.04)
**Attention Consistency**		
Number Stroop *τ*	0.69 (0.10)	
Spatial Stroop Bin 5	0.25 (0.12)	
Arrow Flanker Bin 5	0.37 (0.12)	
Letter Flanker RTsd	0.40 (0.11)	
Circle Flanker *τ*	0.63 (0.09)	
Number Stroop TUTs	0.41 (0.08)	
Arrow Flanker TUTs	0.30 (0.07)	
Letter Flanker TUTs	0.22 (0.07)	
N-Back TUTs	0.27 (0.06)	
**O﻿bjective**/**Objective**^**resid**^		
Number Stroop *τ*	0.20 (0.16)	0.61 (0.04)
Spatial Stroop Bin 5	0.50 (0.09)	0.51 (0.04)
Arrow Flanker Bin 5	0.64 (0.09)	0.67 (0.04)
Letter Flanker RTsd	0.47 (0.11)	0.62 (0.04)
Circle Flanker *τ*	0.32 (0.13)	0.68 (0.04)
**Subjective**/**Subjective**^**resid**^		
Number Stroop TUTS	0.62 (0.07)	0.74 (0.05)
Arrow Flanker TUTS	0.61 (0.07)	0.68 (0.05)
Letter Flanker TUTS	0.45 (0.06)	0.50 (0.05)
N-Back TUTS	0.56 (0.06)	0.63 (0.05)

*OPERSPAN* = operation span, *READSPAN* = reading span, *SYMMSPAN* = symmetry span, *ROTASPAN =* rotation span, *RUNNSPAN* = running span, *COUNTERS* = updating counters, *SART RTSD =* intrasubject standard deviation in RT from SART, *Letter Flanker RTSD =* intrasubject standard deviation in RT from Letter Flanker, *PERCABER1* = perceptual aberration scale (parcel 1), *PERCABER2* = perceptual aberration scale (parcel 2), *PERCABER3* = perceptual aberration scale (parcel 3), *MAGIDEA1* = magical ideation scale (parcel 1), *MAGIDEA2 =* magical ideation scale (parcel 2), *MAGIDEA3 =* magical ideation scale (parcel 3), *REFTHINK* = referential thinking subscale from the Schizotypal Personality Questionnaire (SPQ), *SART* = sustained attention to response task, *TUTs* = TUT rate from task

#### Mini-Multiverse Analyses

The Study 2 mini-multiverse analyses focused on the preregistered bifactor model and the exploratory hierarchical model (without including the exploratory attention control factor, which required removing all SART indicators from the attention consistency models). Our multiverse decisions on outlying RTs and outlying subjects were identical to those for Study 1. Details of the results are presented in Supplemental Materials (see Supplemental Figs. 8 and 9), and we summarize them here.

A series of CFAs on the bifactor model included WMC and positive schizotypy constructs. These iterations resulted in only five of the nine models converging. Although discouraging, these results might not be too surprising given the general instability of bifactor models (e.g., Eid et al., 2017, 2018). Of the models that converged, the resulting correlations were generally consistent with the primary model: WMC was negatively associated, and positive schizotypy was positive associated, with the general attention (in)consistency factor. Further, WMC was not associated with the objective residual (aside from one iteration) and was not associated with the subjective residual in any iteration. Positive schizotypy was not associated with the objective residual in any iteration and was positively associated with the subjective residual in all but one iteration. Thus, despite some iterations failing to converge, those that did presented a reliable pattern. We suggest that the bifactor model is still a promising and theoretically informative way to measure variation in attention consistency, as the individual-differences overlap in objective (performance) and subjective (self-report) measures. At the same time, both Study 1 and Study 2 indicated that bifactor models including both theoretically desirable residual factors (objective and subjective) do not always fit the data adequately and they are not as robust as other models to variation in outlier definitions and treatments.

For the hierarchical model, associations between WMC and positive schizotypy with the second-order attention consistency factor were remarkably consistent across all multiverse iterations. Estimates of the correlation between the second-order factor and WMC were all within 0.03 of the primary correlation and estimates of the correlation with positive schizotypy were all within 0.02. As in Study 1, the hierarchical model of general attention consistency was more robust than the bifactor model. Thus, if one is simply interested in capturing general sustained attention ability, and less so about the residual or separate first-order factors, then the hierarchical model provides a suitable assessment. We note again, however, that *we did not preregister our exploration of the hierarchical model* and so we suggest further independent replication of its fit to attention consistency data and its correlations with other constructs. (It is, of course, encouraging that the hierarchical model results were similar across both Studies 1 and 2.) Moreover, although bifactor models should be assessed for their robustness, their results are important to the central claim that we make here, that the *shared variance* among objective and subjective assessments provides a construct valid measurement of attention consistency. Because the general factor from the bifactor model reflects variance common to objective and subjective measures that is orthogonal to objective measure-specific and subjective-measure-specific variance, its associations and dissociations with other constructs provides the most compelling incremental-validity evidence for (or against) the general factor’s construct validity.

### Discussion

Study 2 provided additional and strong evidence for the construct validity of general attention consistency factors that reflect the shared individual-differences variance in performance variability and self-reported TUT rates. Using an independent dataset (Kane et al., 2016) from that in Study 1 (Unsworth et al., 2021), we captured the proposed full bifactor structure of attention consistency, as well as the hierarchical structure explored in Study 1. We also assessed the associations of the general attention consistency factors (from the bifactor and hierarchical models) with WMC and positive schizotypy. Consistent with Study 1, WMC correlated negatively with the general attention (in)consistency factor (i.e., the shared variance among objective and subjective measures) with a moderate effect size (≈ − 0.40 to − 0.50), despite correlating only weakly with the subjective attention consistency factor from the 2-factor model (− 0.19): higher-WMC subjects exhibited better attention consistency than did lower-WMC subjects. WMC was only associated with the general factor, and not with the objective or subjective residual factors, from the bifactor model.

As predicted, positive schizotypy was weakly to moderately related to general attention (in)consistency in the bifactor and hierarchical models (≈ 0.20–0.30) and also (weakly) positively related to the subjective residual factor in the bifactor model (0.13). Subjects scoring high in positive schizotypy may therefore have general reporting biases or self-beliefs that guide their answers to self-report questions about their thoughts or behaviors, in addition to difficulties with sustaining attention consistency.

Our *non-preregistered* analyses featuring a modified attention (interference) control factor also provided additional validity evidence that we were unable to examine in Study 1. The attention control factor (comprised of antisaccade-arrows errors, antisaccade-letters errors, and SART dʹ) was moderately correlated with the attention consistency factor (0.31) from the bifactor model, indicating that poor attention control of interference and distraction was related to less effective sustaining of momentary attention. This was also true in the hierarchal model, where the correlation between attention control and attention consistency was stronger (0.71). Note that in the 2-factor model of Study 1, attention control was strongly correlated with the objective attention consistency factor (*r* = − 0.86), which at first glance, would suggest that these two constructs may be isomorphic. However, the results from Study 2 suggest that attention consistency and attention control constructs are not redundant.^8^

We conducted a multiverse analysis like that of Study 1 to assess the robustness of our primary CFA results. The results provided some confirmatory evidence for the reliability of the results but also raised some concerns. Specifically, only half of the multiverse iterations for the bifactor model converged, indicating that it is not robust to different outlier treatments (although the converging models yielded consistent estimates of associations with WMC and positive schizotypy). In contrast, the exploratory (*non-preregistered*) hierarchical model of attention consistency was reliable across different outlier decisions: all iterations converged and all estimates of the general factor’s correlations with WMC and positive schizotypy replicated well across the iterations. Despite not being preregistered, then, the hierarchical model was robust across the multiverse, and robust across Studies 1 and 2.

The consistency of our findings across two studies using different attention consistency tasks provides strong supportive evidence for our proposed measurement approach. We argue that it is important to measure individual differences in attention consistency using both objective and subjective indicators, given the possibility of their tapping different degrees of disengagement, as well their independent sources of measurement error. Both the bifactor and hierarchical approaches to modeling the common variance across objective and subjective measures will provide further insight into the nomological network of the general ability to sustain momentary attention.

## General Discussion

The present studies examined the construct validity of attention consistency measures (Unsworth & Miller, 2021) in two independent datasets using different tasks (Kane et al., 2016; Unsworth et al., 2021). The primary goals were to (1) test whether the individual-differences covariation between objective and subjective measures provided a construct valid assessment of the ability to sustain attention consistency and (2) examine how cognitive, contextual, and dispositional nomological network constructs were associated with attention consistency to assess convergent and discriminant validity of the general attention consistency factor. As a secondary goal, we conducted a “mini-multiverse” analysis on each dataset to assess the robustness of our findings against plausible trial-level and subject-level outlier definitions and decisions.

Regarding our first goal, we found that objective and subjective attention consistency indicators share variance and thus load onto a common factor. Although we could not successfully model a full bifactor structure in Study 1, we were able to fit reduced bifactor models that separately captured unique variance to each indicator type. In Study 2, moreover, we were able to fit a full bifactor model to the attention consistency data. These bifactor models presented some problems, however. In Study 1, the reduced bifactor models yielded general factors that were each dominated by the non-residual-factor indicators, which biased the measurement of the general factor toward either the objective or the subjective measures. In Study 2, the full bifactor model was not robust across all mini-multiverse iterations, as some models did not converge. Although the full bifactor model in Study 2 was informative regarding construct validity, as it demonstrated across the converging iterations that a common attention consistency factor correlated with theoretically relevant constructs independently of objective measure-specific and subjective-measure-specific variance (i.e., it indicated incremental validity), the bifactor approach may not always be viable or pragmatic for assessing the general ability to sustain momentary attention.

In both studies, however, we also fit an exploratory (i.e., non-preregistered) hierarchical structure, which modeled general attention consistency as a higher-order factor over the objective indicator and subjective-indicator latent variables. This hierarchical approach allowed us to model the individual-differences overlap in objective and subjective indicators as a general attention consistency construct. Unlike the bifactor models, the hierarchical model adequately fit the data in both Studies 1 and 2, and its fit and nomological network associations were robust across multiverse iterations.

Regarding our second goal, we found evidence for convergent and discriminant validity of the common attention consistency factor. First, individual differences in cognitive ability (i.e., WMC and processing speed) correlated moderately to strongly with the common factor in hypothesized ways: subjects with better cognitive abilities showed better attention consistency. Second, individual difference in contextual variables, such as self-reported motivation and alertness, also correlated strongly with the common attention consistency factor: subjects who reported being more motivated and alert during the cognitive tasks also showed better attention consistency. Finally, dispositional characteristics provided evidence of both convergent and discriminant validity: self-reported everyday cognitive failures and positive schizotypy experiences reliably (if moderately) correlated with the common attention consistency factor, indicating that subjects who reported more of these behaviors and experiences also demonstrate poorer momentary sustained attention. Although big-five personality traits like neuroticism, conscientiousness, and agreeableness correlated with the common factor in some models, but not in others, extraversion and openness did not significantly correlate with the common factor in any model.

In exploratory (*non-preregistered*) analyses, we were able to also assess how a (reduced) attention control factor, derived from a few response-conflict tasks, correlated with attention consistency. Attention control correlated moderately to strongly with the general attention consistency factor, providing more evidence for convergent validity. Further, the attention control correlations provided additional evidence for discriminant validity. Specifically, the general attention consistency factor correlated more strongly with attention control (in both bifactor and hierarchical models) than with WMC. WMC tasks involve processes like memory retrieval and strategy choices that aren’t necessary in attention tasks, which may contribute to the weaker WMC correlation. Given the exploratory nature of this analysis, we suggest that future research attempt to replicate the findings and further explore the dissociation between attention control (as captured by response- or distractor-conflict tasks) and attention consistency.

### General Ability to Sustain Attention Consistency as the Covariation Between Objective and Subjective Indicators

Both studies found that objective and subjective indicators of attention consistency are moderately correlated with each other, replicating prior work (e.g., Kane et al., 2016; Unsworth, 2015; Unsworth et al., 2021; Welhaf et al., 2020). Moreover, we argue that this covariation indicates the presence of a common underlying factor of attention consistency that is psychologically meaningful. That is, individual differences in a general ability to sustain momentary attention can partly explain RT variability and mind-wandering propensity during simple cognitive tasks. Our findings extend prior work that has investigated these measures (e.g., RT variability/performance and TUTs) as separate but related constructs (or that has used objective attention consistency measures to validate subjective measures). This covariation approach is important because objective and subjective measures of attention consistency use two very different methods to assess the same proposed ability. Because each of these measurement types may capture different degrees of task disengagement (à la Cheyne et al., 2009), and each is influenced by different confounding processes, relying on either type of measurement alone may lead to improper conclusions about how the ability to sustain momentary attention relates to other psychological constructs.

At the task level, many potential objective indicators of attention consistency are redundant with one another (see also Unsworth et al., 2020). In both studies, correlations among the individual measurement types (e.g., RTsd, *τ*, slowest RTs) were high in nearly all tasks. For many of the sustained attention tasks used in the literature, then, researchers may use any of several indicators to objectively measure attention consistency. However, in the SART, different indicators might reflect different degrees of attention failures, as they shared some variance but were not redundant: RTsd, a commonly used measure from the SART, showed moderate to strong bivariate correlations (*r*s = 0.30–0.69) with omission errors (both in Study 1 and 2), and with *τ* (in Study 1). Thus, these different SART indicators may capture failures of attention consistency that range from subtle fluctuations to instances of more complete disengagement (Cheyne et al., 2009; Unsworth et al., 2021).

### The Attention Consistency Factor: Construct Validity and Measurement Recommendations

Our initially proposed model of attention consistency was a bifactor structure in which common variance across objective and subjective indicators could be captured by a general factor, and residual variance unique to each indicator type could be modeled as orthogonal measurement-specific factors. This was our preregistered and theoretical starting point because bifactor models allow researchers to assess incremental validity, that is, the general factor’s associations with other construct that are independent of any specific factors. The WMC correlation with the general attention consistency factor in the Study 2 bifactor model (and its lack of correlation with the measurement-specific factors) indicates that performance measures capturing processing speed do not account for any attention consistency–WMC relationship. Unfortunately, from a pragmatic perspective, the bifactor approach turned out to be inappropriate in Study 1 and its fit was not robust to varied outlier treatments in Study 2 (although, where the bifactor model fit it demonstrated stable paths between latent factors). We therefore provisionally recommend against taking a bifactor approach for measuring general attention consistency in most studies (unless sample size is large, multiverse analyses test robustness, and theoretical claims about the nature of the general factor are central).

Instead, when researchers are primarily interested in the general factor of attention consistency, a hierarchical model is a worthy alternative. Studies 1 and 2 suggest that attention consistency can be robustly modeled as a higher-order factor representing the variance shared between objective indicator and subjective-indicator factors. Our hierarchical models provided adequate fit and stood up well across outlier definitions and decisions in mini-multiverse analyses. Thus, *although not preregistered*, the hierarchical approach can allow researchers to assess individual differences in general ability to sustain momentary attention.

With caveats about both bifactor and hierarchical models in mind (see additional discussion of limitations below), the present results speak to the construct validity of the general attention consistency factor. We focus this discussion on the hierarchical models of Study 1 and Study 2 and the full bifactor model in Study 2, as they provided the most unbiased estimates of the general factor.

While many correlations with the general factor were slightly stronger than those with the separate objective-measure or subjective-measure factor, they were much stronger than the weakest correlations in the simple two-factor model. This increase in correlation magnitudes, and the ability to model shared variance using the bifactor approach in Study 2, provides incremental validity evidence of the higher-order factor. That is, the general factor, and the common factor from the bifactor model, show stronger correlations than either of the individual factors (and either of the residual method-specific factors in the bifactor model). Specifically, WMC correlated moderately with the objective factor and weakly with subjective factor in both studies (see also Kane et al., 2016; Unsworth, 2015; Unsworth et al., 2021). WMC correlated substantially, however, with the general factor of attention consistency across models (*r*s = 0.40–0.50). By typically focusing on only objective or subjective indicators, then, prior work may have misestimated the WMC association with the ability to sustain momentary attention. If TUT rates reflect a study’s only measure of attention consistency, researchers may interpret its relation to WMC to be weak. Using the overlap in objective and subjective indicators, in contrast, provides evidence that the link between WMC and attention consistency is rather strong, and perhaps even stronger than associations with either indicator on their own.

Processing speed similarly showed much stronger correlations with the objective than the subjective measures, but its correlation with the common factor was strong (*r* = − 0.59), and stronger than with either method-specific correlation. Thus, processing speed may be more tied to general attention consistency than previous work has shown, especially given the previously mixed results (Unsworth et al., 2021; Welhaf et al., 2020). Of theoretical importance, when attention consistency is defined as a general factor derived from both TUT rates and performance variability, it cannot be reduced to a processing speed construct. When attention consistency is instead only defined by RT variability measures, however, the close link between *M* RT and RT variability makes it difficult to differentiate attention consistency from processing speed.

Attention consistency correlations with dispositional measures provided the clearest evidence of discriminant validity. In Study 1, conscientiousness and agreeableness both correlated with the subjective (TUT rate) factor. Neither variable, however, correlated with the general factor in the hierarchical model, suggesting that these traits are not related to the general ability to sustained momentary attention. If a study used only TUT rates to measure attention consistency, it might erroneously infer a robust association. Further, neuroticism correlated weakly with the subjective factor and nonsignificantly with the objective factor, but it correlated with the general attention consistency factor as strongly as it had with the subjective factor, suggesting a modest relationship with attention consistency that goes beyond possible self-report biases. Finally, self-reported cognitive failures and positive schizotypy both correlated more strongly with the common factor than with either the individual objective or subjective factors, again suggesting associations that reflect more than shared method variance. Using the covariation between objective and subjective indicators as a measure of attention consistency, then, correlations with some trait factors appear to be reliable. Future research should aim to replicate attention consistency × personality relationships and consider other dispositional factors that may inform the nomological network of sustained attention measures, such as daily stress, rumination-proneness, trait anxiety, and ADHD-related symptoms.

The present studies leave us, however, with a lingering theoretical question: how are attention control and attention consistency related? Some definitions of attention control and attention consistency share considerable conceptual overlap when the former invoke goal maintenance or proactive control mechanisms. As well, errors in attention control tasks (due to goal-maintenance or proactive-control failures) manifest in task errors and long RTs, which we have used to measure attention consistency in the present studies. Thus, these abilities may be empirically indistinguishable in some contexts, perhaps especially when attention consistency measures are taken from tasks that have a strong goal-maintenance or proactive control requirement. Future studies may therefore need to model attention control using tasks that have a strong goal-maintenance/proactive control requirement due to response or stimulus conflict (e.g., antisaccade, high congruency Stroop, AX-CPT tasks), while modeling attention consistency using tasks that don’t (e.g., PVT, MRT, and simple/low-choice RT tasks; see Unsworth et al., 2021). If these constructs are still highly correlated after making their measures as independent as possible, then it might indicate that these two abilities are two sides of the same coin.

## Conclusions

Attention consistency is an understudied individual-differences construct (Unsworth & Miller, 2021), given its important contributions to successful performance of many laboratory tasks and everyday activities. The results of the current re-analyses suggest that individual differences in attention consistency, as measured by the shared variance across objective (performance) and subjective (self-report) indicators, can provide a more construct valid measurement than either of these methods separately. Specifically, hierarchical models of attention consistency may be a suitable—if not optimal—approach for most researchers interested in estimating general sustained attention ability. Objective and subjective measures of attention consistency may capture different attentional states along a continuum of disengagement (Cheyne et al., 2009) and each measurement type has its own unique sources of measurement error. So, relying only on one of these measurement approaches can lead to biased, improper conclusions about the association between the ability to sustain momentary attention and other nomological network constructs. Indeed, we found that some constructs’ correlations were *stronger* with the general attention consistency factor than with either the objective or subjective factor alone, which suggests a possible underestimation of the link between sustained attention and other factors in its nomological network in prior research.

### Electronic supplementary material

Below is the link to the electronic supplementary material.

Supplementary file1 (DOCX 2058 KB)

## Data Availability

All data and R scripts for reproducing the analyses can be found on Open Science Framework (https://osf.io/xeu63/).

## Appendix A. Trial cleaning descriptive statistics for Study 1

**Table:**

Task	*M*	SD	Median	Min	Max
**PVT**					
Post-error/probe trials removed	16.78	6.22	16.00	5.00	48.00
Anticipations removed	0.07	0.26	0.00	0.00	2.00
Censored trials	1.66	1.54	1.00	0.00	10.00
**SART**					
Post-error/probe trials removed	47.93	16.69	46.00	22.00	109.00
Anticipations removed	9.90	17.97	3.00	0.00	141.00
Censored trials	2.14	3.27	1.00	0.00	18.00
**CRT**					
Post-error/probe trials removed	7.64	5.79	6.00	0.00	45.00
Anticipations removed	0.28	0.89	0.00	0.00	6.00
Censored trials	2.58	2.59	2.00	0.00	15.00
**Continuous tracking**					
Post-error/probe trials removed	–	–	–	–	–
Anticipations removed	–	–	–	–	–
Censored trials	423.25	162.98	416.00	0.00	1144.00

## Appendix B. Trial cleaning descriptive statistics for Study 2

**Table:**

Task	*M*	SD	Median	Min	Max
**SART**					
Post-error/probe trials removed	84.63	32.04	73.50	45.00	226.00
Anticipations removed	14.25	25.03	3.00	0.00	181.00
Censored trials	8.76	9.31	6.00	0.00	60.00
**Number Stroop**					
Post-error/probe trials removed	36.78	11.05	34.00	24.00	123.00
Anticipations removed	0.07	0.78	0.00	0.00	13.00
Censored trials	4.52	3.89	4.00	0.00	33.00
**Spatial Stroop**					
Post-error/probe trials removed	3.71	4.75	2.00	0.00	33.00
Anticipations removed	0.01	0.11	0.00	0.00	2.00
Censored trials	1.51	1.55	1.00	0.00	10.00
**Arrow flanker**					
Post-error/probe trials removed	45.26	21.37	40.00	19.00	188.00
Anticipations removed	0.10	0.75	0.00	0.00	11.00
Censored trials	1.88	1.58	2.00	0.00	8.00
**Letter flanker**					
Post-error/probe trials removed	14.56	4.53	14.00	11.00	62.00
Anticipations removed	0.00	0.05	0.00	0.00	1.00
Censored trials	1.45	1.44	1.00	0.00	7.00
**Circle flanker**					
Post-error/probe trials removed	8.03	5.85	7.00	0.00	40.00
Anticipations removed	0.01	0.24	0.00	0.00	5.00
Censored trials	1.73	1.60	1.00	0.00	10.00

## Footnotes

1 The heart rate literature provides variability indicators that might be profitably considered in attention-consistency research (Pham et al., 2021). Indices like the root mean square of successive difference (RMSSD) capture differences between successive intervals and capture short-term variations in heartrate.

2 The SART presents a unique case for assessing lapses of attention. Namely, because of the high frequency of “go” responses that build up habitual, mindless responding, extremely fast responses might also be indicative of attention lapses. Indeed, prior research has found that TUT rates in a SART are significantly correlated with the fastest 20% of responses, in addition to the slowest; that is, individuals who mind wander more in that SART also have shorter “short” RTs along with longer “long” RTs (McVay & Kane, 2012a; Welhaf et al., 2020). Thus, the fastest 20% of SART RTs has been included as a possible indicator of attention consistency.

3 Unsworth et al. (2021) also measured lapses in a whole report working memory task, as the number of trials where subjects recalled ≤ 1 item. We did not include this lapses measure because some may have reflected working memory failures. Additionally, we did not include self-reports of sleep quality, boredom proneness, or mindfulness, the latter due to multicollinearity problems (reported in Unsworth et al., 2021).

4 We dropped the fastest 20% variable rather than other SART measures as it correlated only weakly with RTsd and Omissions (*r*s < 0.20). RTsd, *τ*, and Omissions all correlated with each other at *r* ≥ 0.25.

5 *Although not preregistered*, we also tested whether a single-factor Attention Consistency model fit the data, by specifying a model with all the objective and subjective indicators loaded onto a single latent variable. This model fit the data poorly, *χ*^2^(28) = 237.438, CFI = 0.745, TLI = 0.590, RMSEA [90% CI] = 0.147 [0.130–0.165], SRMR = 0.117. A chi-square differences test also indicated that the two-factor model fit significantly better than the one-factor model (Δ*χ*^2^ (1) = 183.08, *p* < 0.001).

6 As a preregistered exploratory set of analyses, we also investigated what the objective residual factor might reflect. However, the results here were especially complicated by the need to use the reduced bifactor model with a general factor biased toward the TUT indicators. Our original hypothesis was that the objective residual factor might primarily represent processing speed, given that many of the contributing indicators are RT-based. Or, that it may reflect strategy choice in attention tasks (i.e., speed-accuracy trade-off). Although the reduced bifactor structure of the model prevented us from drawing clear conclusions, we present the results of these preregistered analyses in Supplemental Table 7.

7 *Although not preregistered*, we tested whether a single-factor Attention Consistency model fit the data, as we did in Study 1. To do so, we specified a model where all the objective and subjective indicators loaded onto one latent variable. This model did not adequately fit the data, *χ*^2^(46) = 317.381, CFI = 0.825, TLI = 0.749, RMSEA [90% CI] = 0.106 [0.095–0.117], SRMR = 0.101. A chi-square differences test also indicated that the two-factor model was a significantly better fitting model (Δ*χ*^2^ (1) = 208.17, *p* < 0.001).

8 We are unsure why the attention control × attention consistency correlation differed so much between the bifactor and hierarchical models in Study 2. Perhaps it is because the bifactor approach models both shared objective–subjective variance and variance that is unique to each. As another possibility, a closer look at the confidence intervals (CI) suggests that the hierarchical model may have provided a better estimate, as the CI was smaller than in the bifactor model (Hierarchical CI = [0.56, 0.85]; Bifactor CI = [0.09, 0.53]).
