# Supplementary material for: A Nomothetic Span Approach to the Construct Validation of Sustained Attention Consistency: Re-Analyzing Two Latent-Variable Studies of Performance Variability and Mind-Wandering Self-Reports
Source: Psychol Res. 2023 Jun 14;88(1):39–80. doi: 10.1007/s00426-023-01820-0 (PMC10805875; doi:10.1007/s00426-023-01820-0)
Supplement: Supplementary file 1 — Supplementary file1 (DOCX 2058 KB) [file 426_2023_1820_MOESM1_ESM.docx]

Supplemental Table 1. Descriptive Statistics for all potential Objective Attention Consistency Measures for Study 1

|  | Mean | SD | Min | Max | Skew | Kurtosis | Reliability | N | N_censored_ |
| --- | --- | --- | --- | --- | --- | --- | --- | --- | --- |
| **PVT** |  |  |  |  |  |  |  |  |  |
| Slowest 20% | 459.48 | 99.29 | 307.08 | 789.01 | 1.37 | 2.18 | .98 | 345 | 10 |
| Tau | 63.52 | 34.42 | 0.00 | 167.62 | 1.27 | 1.40 | -- | 345 | 12 |
| Lapses | 2.83 | 3.70 | 0.00 | 13.00 | 1.33 | 0.82 | .93 | 345 | 15 |
| Blocks | 0.00 | 0.00 | 0.00 | 0.00 | -- | -- | .93 | 345 | 39 |
| RTsd | 65.45 | 31.60 | 17.69 | 163.00 | 1.38 | 1.69 | .97 | 345 | 12 |
| RTmad | 50.79 | 23.98 | 17.05 | 126.02 | 1.34 | 1.68 | .85 | 345 | 10 |
| **CRT** |  |  |  |  |  |  |  |  |  |
| Slowest 20% | 562.93 | 110.83 | 340.62 | 917.29 | 1.07 | 1.32 | .99 | 347 | 6 |
| Tau | 91.56 | 38.05 | 26.32 | 229.59 | 1.27 | 2.15 | .92 | 347 | 4 |
| Blocks | 0.00 | 0.00 | 0.00 | 0.00 | -- | -- | .92 | 347 | 55 |
| RTsd | 99.50 | 33.31 | 33.52 | 213.82 | 1.21 | 1.89 | .98 | 347 | 6 |
| RTmad | 89.19 | 29.88 | 25.20 | 201.63 | 1.06 | 1.74 | .94 | 347 | 3 |
| RMSSD | 128.59 | 41.49 | 46.83 | 274.75 | 1.24 | 2.17 | .96 | 347 | 6 |
| **SART** |  |  |  |  |  |  |  |  |  |
| Slowest 20% | 670.60 | 145.75 | 324.00 | 1149.72 | 0.46 | 1.05 | .99 | 334 | 0 |
| Tau | 101.18 | 67.14 | 2.30 | 328.24 | 1.01 | 1.28 | .94 | 334 | 4 |
| Blocks | 1.42 | 2.20 | 0.00 | 6.00 | 1.24 | -0.09 | .96 | 334 | 44 |
| RTsd | 133.25 | 49.33 | 44.07 | 299.83 | 1.11 | 1.80 | .99 | 334 | 8 |
| RTmad | 118.10 | 48.58 | 28.17 | 279.47 | 0.86 | 0.94 | .96 | 334 | 5 |
| RMSSD | 102.55 | 30.36 | 31.24 | 219.06 | 0.67 | 0.82 | .87 | 334 | 0 |
| Fastest 20% | 307.55 | 69.24 | 204.58 | 551.09 | 1.05 | 1.16 | 1.00 | 334 | 3 |
| Omissions | 19.02 | 14.70 | 0.00 | 70.00 | 1.30 | 1.77 | .93 | 334 | 4 |
| **Continuous Tracking** |  |  |  |  |  |  |  |  |  |
| Tracking Distance Variability (RRT) | 1.15 | 0.41 | 0.24 | 2.56 | 0.67 | 0.59 | .95 | 334 | 2 |
| Flat Spots | 0.00 | 0.00 | 0.00 | 0.00 | 0.00 | 0.00 |  | 334 | 80 |
| Mean Tracking Error | 15.00 | 6.16 | 7.26 | 29.62 | 1.31 | 0.68 | .98 | 334 | 30 |
| Tracking Error iSD | 11.02 | 5.07 | 5.32 | 22.62 | 1.37 | 0.62 | .95 | 334 | 34 |

Supplemental Table 2. Descriptive Statistics for all potential Processing Speed Measures for Study 1

|  | Mean | SD | Min | Max | Skew | Kurtosis | Reliability | N | N_censored_ |
| --- | --- | --- | --- | --- | --- | --- | --- | --- | --- |
| **PVT** |  |  |  |  |  |  |  |  |  |
| Mu | 287.04 | 29.07 | 227.26 | 393.63 | 0.78 | 0.66 | -- | 345 | 2 |
| Fastest 20% | 283.78 | 25.34 | 236.64 | 377.50 | 0.96 | 1.63 | .98 | 345 | 5 |
| **CRT** |  |  |  |  |  |  |  |  |  |
| Mu | 316.95 | 49.24 | 226.21 | 501.23 | 0.98 | 1.38 | .93 | 347 | 2 |
| Fastest 20% | 294.95 | 36.68 | 227.19 | 434.92 | 1.06 | 1.51 |  | 347 | 2 |
| **SART** |  |  |  |  |  |  |  |  |  |
| Mu | 670.60 | 145.75 | 324.00 | 1149.72 | 0.46 | 1.05 | .99 | 334 | 0 |
| **Stroop** |  |  |  |  |  |  |  |  |  |
| Fastest 20% | 440.66 | 65.96 | 304.45 | 675.58 | 0.95 | 1.26 | .96 | 352 | 3 |
| Mu | 463.80 | 77.11 | 294.24 | 724.05 | 1.00 | 1.11 | -- | 352 | 3 |
| **Prosaccade** |  |  |  |  |  |  |  |  |  |
| Median RT | 713.04 | 252.06 | 305.50 | 1628. | 1.10 | 1.66 | .85 | 323 | 3 |

Supplemental Table 3. Zero-order correlations of new objective indicator variables in Study 1

| Variable | 1 | 2 | 3 | 4 | 5 | 6 | 7 | 8 | 9 | 10 | 11 | 12 |
| --- | --- | --- | --- | --- | --- | --- | --- | --- | --- | --- | --- | --- |
| 1 PVT_Bin5 | 1 |  |  |  |  |  |  |  |  |  |  |  |
| 2 PVT_RTsd | 0.97 | 1 |  |  |  |  |  |  |  |  |  |  |
| 3 PVT_Tau | 0.94 | 0.98 | 1 |  |  |  |  |  |  |  |  |  |
| 4 PVT_RTmad | 0.92 | 0.93 | 0.90 | 1 |  |  |  |  |  |  |  |  |
| 5 PVT_Lapse | 0.94 | 0.92 | 0.91 | 0.87 | 1 |  |  |  |  |  |  |  |
| 6 PVT_Blocks |  |  |  |  |  | 1 |  |  |  |  |  |  |
| 7 CRT_Tau | 0.41 | 0.38 | 0.36 | 0.39 | 0.36 |  | 1 |  |  |  |  |  |
| 8 CRT_RMSSD | 0.42 | 0.38 | 0.35 | 0.39 | 0.36 |  | 0.89 | 1 |  |  |  |  |
| 9 CRT_RTsd | 0.45 | 0.42 | 0.39 | 0.43 | 0.40 |  | 0.93 | 0.97 | 1 |  |  |  |
| 10 CRT_Bin5 | 0.41 | 0.36 | 0.33 | 0.39 | 0.36 |  | 0.83 | 0.95 | 0.95 | 1 |  |  |
| 11 CRT_Blocks |  |  |  |  |  |  |  |  |  |  | 1 |  |
| 12 CRT_RTmad | 0.43 | 0.39 | 0.35 | 0.40 | 0.36 |  | 0.85 | 0.94 | 0.93 | 0.90 |  | 1 |
| 13 SART_Tau | 0.26 | 0.25 | 0.22 | 0.27 | 0.27 |  | 0.18 | 0.15 | 0.18 | 0.13 |  | 0.19 |
| 14 SART_RTsd | 0.30 | 0.29 | 0.27 | 0.30 | 0.32 |  | 0.19 | 0.22 | 0.25 | 0.23 |  | 0.27 |
| 15 SART_RMSSD | 0.33 | 0.33 | 0.30 | 0.34 | 0.32 |  | 0.33 | 0.35 | 0.38 | 0.35 |  | 0.36 |
| 16 SART_RTmad | 0.24 | 0.22 | 0.19 | 0.24 | 0.27 |  | 0.13 | 0.17 | 0.20 | 0.19 |  | 0.23 |
| 17 SART_Bin5 | 0.16 | 0.15 | 0.13 | 0.14 | 0.18 |  | 0.09 | 0.15 | 0.16 | 0.20 |  | 0.19 |
| 18 SART_Bin1 | -0.19 | -0.21 | -0.20 | -0.23 | -0.19 |  | -0.18 | -0.11 | -0.13 | -0.02 |  | -0.11 |
| 19 SART_Blocks | 0.25 | 0.24 | 0.19 | 0.24 | 0.27 |  | 0.20 | 0.24 | 0.26 | 0.27 |  | 0.27 |
| 20 SART_Omissions | 0.26 | 0.28 | 0.25 | 0.28 | 0.25 |  | 0.36 | 0.37 | 0.37 | 0.32 |  | 0.37 |
| 21 Continuous Tracking iSD | 0.36 | 0.37 | 0.37 | 0.34 | 0.34 |  | 0.37 | 0.42 | 0.42 | 0.37 |  | 0.42 |
| 22 Continuous Tracking Average Error | 0.34 | 0.34 | 0.34 | 0.32 | 0.32 |  | 0.36 | 0.42 | 0.42 | 0.38 |  | 0.42 |
| 23 Continuous Tracking RRT | 0.38 | 0.39 | 0.38 | 0.37 | 0.36 |  | 0.39 | 0.45 | 0.45 | 0.39 |  | 0.46 |
| 24 Continuous Tracking FlatSpots |  |  |  |  |  |  |  |  |  |  |  |  |

Note. Missing correlations are due to censoring creating a 0 score for each subject on the count variables.

Supplemental Table 3. Zero-order correlations of new objective indicator variables in Study 1

| Variable | 13 | 14 | 15 | 16 | 17 | 18 | 19 | 20 | 21 | 22 | 23 | 24 |
| --- | --- | --- | --- | --- | --- | --- | --- | --- | --- | --- | --- | --- |
| 1 PVT_Bin5 |  |  |  |  |  |  |  |  |  |  |  |  |
| 2 PVT_RTsd |  |  |  |  |  |  |  |  |  |  |  |  |
| 3 PVT_Tau |  |  |  |  |  |  |  |  |  |  |  |  |
| 4 PVT_RTmad |  |  |  |  |  |  |  |  |  |  |  |  |
| 5 PVT_Lapse |  |  |  |  |  |  |  |  |  |  |  |  |
| 6 PVT_Blocks |  |  |  |  |  |  |  |  |  |  |  |  |
| 7 CRT_Tau |  |  |  |  |  |  |  |  |  |  |  |  |
| 8 CRT_RMSSD |  |  |  |  |  |  |  |  |  |  |  |  |
| 9 CRT_RTsd |  |  |  |  |  |  |  |  |  |  |  |  |
| 10 CRT_Bin5 |  |  |  |  |  |  |  |  |  |  |  |  |
| 11 CRT_Blocks |  |  |  |  |  |  |  |  |  |  |  |  |
| 12 CRT_RTmad |  |  |  |  |  |  |  |  |  |  |  |  |
| 13 SART_Tau | 1 |  |  |  |  |  |  |  |  |  |  |  |
| 14 SART_RTsd | 0.63 | 1 |  |  |  |  |  |  |  |  |  |  |
| 15 SART_RMSSD | 0.59 | 0.74 | 1 |  |  |  |  |  |  |  |  |  |
| 16 SART_RTmad | 0.46 | 0.83 | 0.60 | 1 |  |  |  |  |  |  |  |  |
| 17 SART_Bin5 | 0.35 | 0.86 | 0.65 | 0.72 | 1 |  |  |  |  |  |  |  |
| 18 SART_Bin1 | -0.45 | -0.18 | -0.07 | -0.11 | 0.34 | 1 |  |  |  |  |  |  |
| 19 SART_Blocks | 0.42 | 0.76 | 0.59 | 0.66 | 0.75 | 0.06 | 1 |  |  |  |  |  |
| 20 SART_Omissions | 0.37 | 0.27 | 0.20 | 0.07 | -0.01 | -0.55 | 0.15 | 1 |  |  |  |  |
| 21 Continuous Tracking iSD | 0.23 | 0.32 | 0.35 | 0.22 | 0.21 | -0.17 | 0.33 | 0.35 | 1 |  |  |  |
| 22 Continuous Tracking Average Error | 0.23 | 0.30 | 0.32 | 0.21 | 0.20 | -0.15 | 0.32 | 0.33 | 0.97 | 1 |  |  |
| 23 Continuous Tracking RRT | 0.23 | 0.31 | 0.34 | 0.20 | 0.18 | -0.22 | 0.30 | 0.34 | 0.74 | 0.73 | 1 |  |
| 24 Continuous Tracking FlatSpots |  |  |  |  |  |  |  |  |  |  |  | 1 |

Note. Missing correlations are due to censoring creating a 0 score for each subject on the count variables.

Supplemental Table 4. Zero-order correlations of new processing speed measures in Study 1

| Variable | 1 | 2 | 3 | 4 | 5 | 6 | 7 | 8 |
| --- | --- | --- | --- | --- | --- | --- | --- | --- |
| 1 CRT_Mu | 1 |  |  |  |  |  |  |  |
| 2 CRT Bin1 | 0.91 | 1 |  |  |  |  |  |  |
| 3 PVT_Mu | 0.28 | 0.32 | 1 |  |  |  |  |  |
| 4 PVT Bin1 | 0.26 | 0.32 | 0.89 | 1 |  |  |  |  |
| 5 SART_Mu | 0.24 | 0.22 | -0.03 | -0.05 | 1 |  |  |  |
| 6 Stroop Bin1 | 0.55 | 0.59 | 0.40 | 0.43 | 0.10 | 1 |  |  |
| 7 Stroop_Mu | 0.50 | 0.54 | 0.29 | 0.31 | 0.09 | 0.87 | 1 |  |
| 8 Prosaccade Med RT | 0.21 | 0.21 | -0.02 | 0.01 | -0.02 | 0.24 | 0.24 | 1 |

Supplemental Table 5. Latent variable correlations of predictor variables from Study 1 Bifactor Subjective-Residual (below diagonal) and Bifactor Objective residual (above diagonal).

| Construct/Measure | 1 | 2 | 3 | 4 | 5 | 6 | 7 | 8 | 9 | 10 | 11 |
| --- | --- | --- | --- | --- | --- | --- | --- | --- | --- | --- | --- |
| 1) WMC |  | .49 | -.32 | .19 | .21 | .01 | -.06 | -.02 | .00 | -.18 | -.01 |
| 2) Attention Control | .50 |  | -.67 | .42 | .33 | -.01 | -.02 | .02 | -.07 | -.25 | -.09 |
| 3) Processing Speed | -.33 | -.67 |  | -.17 | -.14 | -.05 | .04 | -.00 | .02 | .06 | -.04 |
| 4) Alertness | .19 | .42 | -.17 |  | .77 | .14 | .13 | .05 | .19 | -.13 | -.12 |
| 5) Motivation | .21 | .33 | -.14 | .77 |  | .04 | .14 | .03 | .13 | -.08 | -.08 |
| 6) Openness | .01 | -.00 | -.05 | .15 | .04 |  | -.04 | .15 | -.01 | -.03 | .01 |
| 7) Conscientiousness | -.07 | -.02 | .04 | .14 | .14 | -.04 |  | .10 | .25 | -.19 | -.40 |
| 8) Extraversion | -.02 | .02 | -.00 | .05 | .03 | .15 | .10 |  | .11 | -.26 | -.05 |
| 9) Agreeableness | .00 | -.08 | .02 | .19 | .13 | -.01 | .25 | -.11 |  | -.32 | -.19 |
| 10) Neuroticism | -.18 | -.25 | .06 | -.13 | -.08 | -.03 | -.19 | -.26 | -.32 |  | .40 |
| 11) Cognitive Failures | -.01 | -.09 | -.04 | -.12 | -.08 | .01 | -.40 | .05 | -.19 | .40 |  |

Notes. WMC = Working Memory Capacity

Supplemental Table 6. Latent variable correlations of predictor variables from Study 1 reduced hierarchical model.

| Construct/Measure | 1 | 2 | 3 | 4 | 5 | 6 | 7 |
| --- | --- | --- | --- | --- | --- | --- | --- |
| 1) WMC |  |  |  |  |  |  |  |
| 2) Processing Speed | -.32 |  |  |  |  |  |  |
| 3) Openness | .01 | -.05 |  |  |  |  |  |
| 4) Conscientiousness | -.07 | .03 | -.04 |  |  |  |  |
| 5) Extraversion | -.02 | .00 | .15 | .10 |  |  |  |
| 6) Agreeableness | -.01 | .01 | -.01 | .25 | .12 |  |  |
| 7) Neuroticism | -.18 | .06 | -.03 | -.19 | -.26 | -.32 |  |
| 8) Cognitive Failures | -.01 | -.03 | .01 | -.40 | -.05 | -.19 | -.40 |

Notes. WMC = Working Memory Capacity

Supplemental Table 7. What Does the Objective-Residual Factor Represent?

As a preregistered exploratory analysis, we investigated what the objective-residual factor reflects. One possibility is that this factor primarily represents processing speed, given that many of the contributing indicators are RT based. Another is that this factor reflects strategy choice in sustained attention tasks (i.e., speed-accuracy trade-off). However, because the bifactor models in Study 1 were ultimately biased by an imbalance in the factor loadings, we were not able to reliably estimate the general or residual factors, thus making this additional analysis also biased.

Given this caveat, we ran a new bifactor model that included measures of processing speed as indicators in the model. Here, our objective measures of attention consistency now loaded onto three latent variables: the common attention consistency latent variable, the objective-residual, and a new speed of processing residual (see Supplemental Figure 1). This model provided similar fit to our other measurement models, χ^2^ (df) = 132.037 (56), χ^2^ /df= 2.36, CFI =.935, TLI = .894, RMSEA [90% CI] = .063 [.049-.077], SRMR = .057.

|  |
| --- |

Supplemental Figure 1. Measurement model of the Alternative Processing Speed Bifactor Model. For clarity, factor loadings are not presented here; see Supplemental Table 7.1 for factor loadings.

The objective attention consistency measures loaded onto each factor in varying degrees. All the objective measures (aside from SART τ) significantly loaded onto the speed factor, suggesting that they partially reflect variance in processing speed after accounting for general attention consistency. Critically, there was still enough variance shared among the objective measures to successfully model an objective-residual beyond the processing speed factor. This suggests that our objective attention consistency indicators captured something unique from processing speed (after accounting for general ability to sustain momentary attention). Finally, with the objective measures loading onto the objective residual and speed residual factors, their loadings onto the general attention consistency factor were weak and so that factor was primarily driven by the TUT-rate measures.

|   Supplemental Figure 2. Confirmatory factor analysis of the Alternative Processing Speed Bifactor Model. WMC = Working Memory Capacity. Standardized path estimates are presented. For clarity, factor loadings are not presented here; see Supplemental Table 7.1 for factor loadings. |
| --- |

We next ran a CFA with WMC, Attention Control, and the contextual and dispositional variables included. Model fit was again acceptable and in-line with our other models χ^2^ (df) = 737.850 (433), χ^2^ /df= 1.70, CFI =.911, TLI = .884, RMSEA [90% CI] = .045 [.039-.051], SRMR = .056. Perhaps the biggest changes in the pattern of correlations centered around WMC and Attention Control. As shown in Supplemental Figure 2, after allowing the objective indicators to load onto the Speed factor, WMC and Attention Control no longer significantly correlated with general attention consistency (which reflected primarily variation in subjective measures, i.e., TUT rates). Consistent with our other models, many of the contextual and dispositional variables still correlated with the general attention consistency factor. WMC and Attention Control still correlated with the objective residual as well as with the speed residual. Motivation and Alertness also modestly correlated with the objective residual but did not correlate with the speed residual. Finally, only one dispositional variable significantly correlated with either of the residual factors, and it was weak (extraversion with the objective-residual).

Supplemental Table 8. Standardized factor loadings (and standard errors) for alternative processing speed bifactor models for Study 1

| Construct and Measure | Model Name | |
| --- | --- | --- |
|  | Alternative Processing Speed  Measurement | Alternative Processing Speed  CFA |
| **Working Memory Capacity** |  |  |
| OPERSPAN |  | .71 (.05) |
| READSPAN |  | .64 (.05) |
| SYMSPAN |  | .61 (.05) |
| **Attention Control** |  |  |
| Antisaccade |  | .55 (.05) |
| Cued Visual Search |  | -.56 (.05) |
| Stroop |  | -.17 (.06) |
| **Processing Speed** |  |  |
| CRT Bin 1 | .71 (.05) | .71 (.04) |
| PVT μ | .46 (.05) | .46 (.05) |
| Stroop Bin 1 | .81 (.05) | .80 (.04) |
| Prosaccade M RT | .23 (.06) | .27 (.06) |
| **Alertness** |  |  |
| PVT |  | .84 (.03) |
| CRT |  | .68 (.04) |
| Continuous Tracking |  | .55 (.04) |
| Antisaccade |  | .58 (.04) |
| **Motivation** |  |  |
| PVT |  | .82 (.03) |
| CRT |  | .68 (.04) |
| Continuous Tracking |  | .53 (.04) |
| Antisaccade |  | .55 (.05) |
| **Attention Consistency** |  |  |
| PVT Bin 5 | .20 (.06) | .24 (.06) |
| SART RTSD | .03 (.07) | .03 (.06) |
| SART Omissions | .02 (.07) | .02 (.06) |
| SART τ | .09 (.07) | .09 (.06) |
| CRT τ | .14 (.06) | .12 (.06) |
| Continuous Tracking Variability | .26 (.06) | .23 (.06) |

Supplemental Table 8 (continued). Standardized factor loadings (and standard errors) for alternative processing speed bifactor models for Study 1

| Construct and Measure | Model Name | |  |
| --- | --- | --- | --- |
|  | Alternative Processing Speed  Measurement | Alternative Processing Speed  CFA | |
| PVT TUTs | .62 (.05) | .74 (.04) | |
| SART TUTs | .74 (.04) | .66 (.04) | |
| WRWM TUTs | .65 (.04) | .61 (.05) | |
| Stroop TUTs | .68 (.04) | .66 (.04) | |
| **Objective/Objective^resid^/Speed** |  |  | |
| PVT Bin 5 | .48 (.06) /.30 (.06) | .49 (.05) /.30 (.06) | |
| SART RTSD | .41 (.07) /.23 (.06) | .42 (.07) /.24 (.06) | |
| SART Omissions | .49 (.07) /.17 (.06) | .47 (.06) /.17 (.06) | |
| SART τ | .36 (.07) /.08 (.06) | .37 (.07) /.09 (.06) | |
| CRT τ | .43 (.06) /.39 (.06) | .41 (.06) /.40 (.06) | |
| Continuous Tracking Variability | .59 (.06) /.20 (.06) | .60 (.05) /.21 (.06) | |
| **Subjective/Subjective^resid^** |  |  | |
| PVT TUTs |  |  | |
| SART TUTs |  |  | |
| WRWM TUTs |  |  | |
| Stroop TUTs |  |  | |

*Note*. Bifactor Sub-Res = bifactor model with a subjective-indicator residual factor; Bifactor Obj-Res = bifactor model with an objective-indicator residual factor; OPERSPAN = operation span; READSPAN = reading span; SYMMSPAN = symmetry span; PVT Bin 1 = Mean RT of the fastest 20% of trials in the PVT; PVT Bin 5 = Mean RT of the slowest 20% of trials in the PVT; SART RTSD = intrasubject standard deviation in RT from SART; PVT = Psychomotor Vigilance Task; SART = Sustained Attention to Response Task. CRT = Choice Reaction Time Task; WRWM = Whole Report Working Memory task; TUTs = TUT rate from task.

Supplemental Table 9. Figures for Study 1 multiverse analyses

| *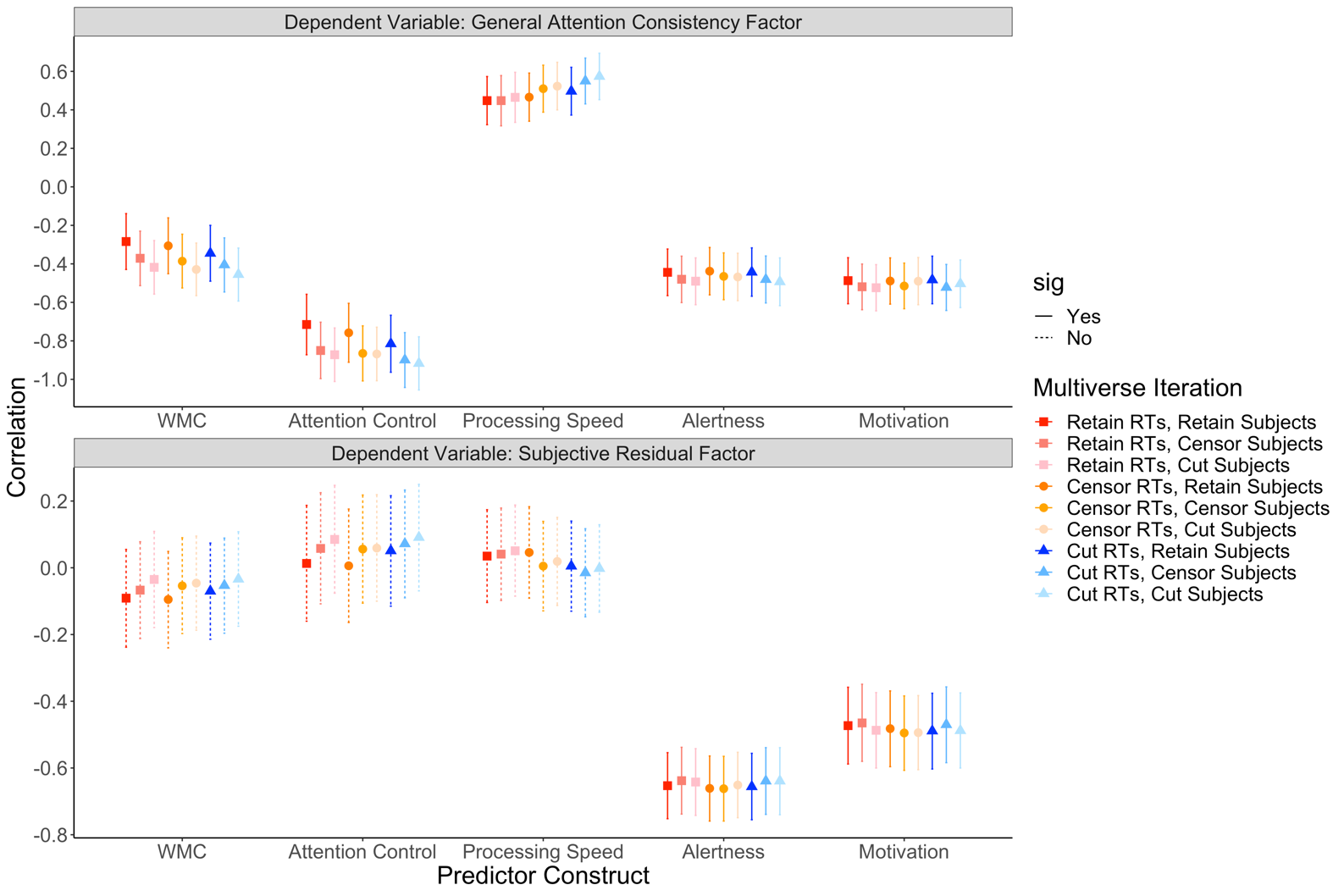*  Supplemental Figure 3. Multiverse analysis results for Study 1 Subjective-Residual Bifactor Model for Cognitive and Contextual Predictors. |
| --- |

| 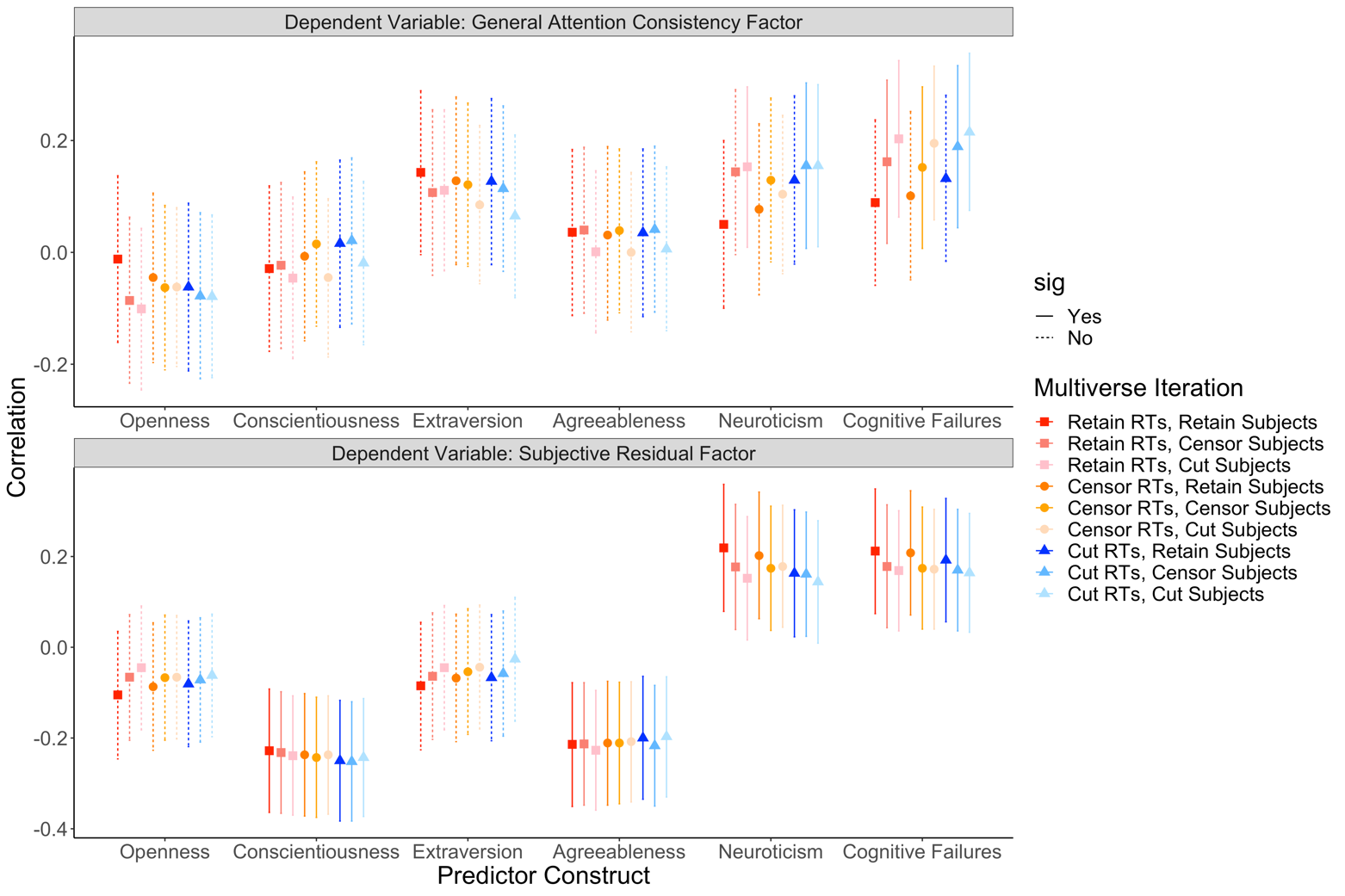  Supplemental Figure 4. Multiverse analysis results for Study 1 Subjective-Residual Bifactor Model for Dispositional Predictors. |
| --- |

| 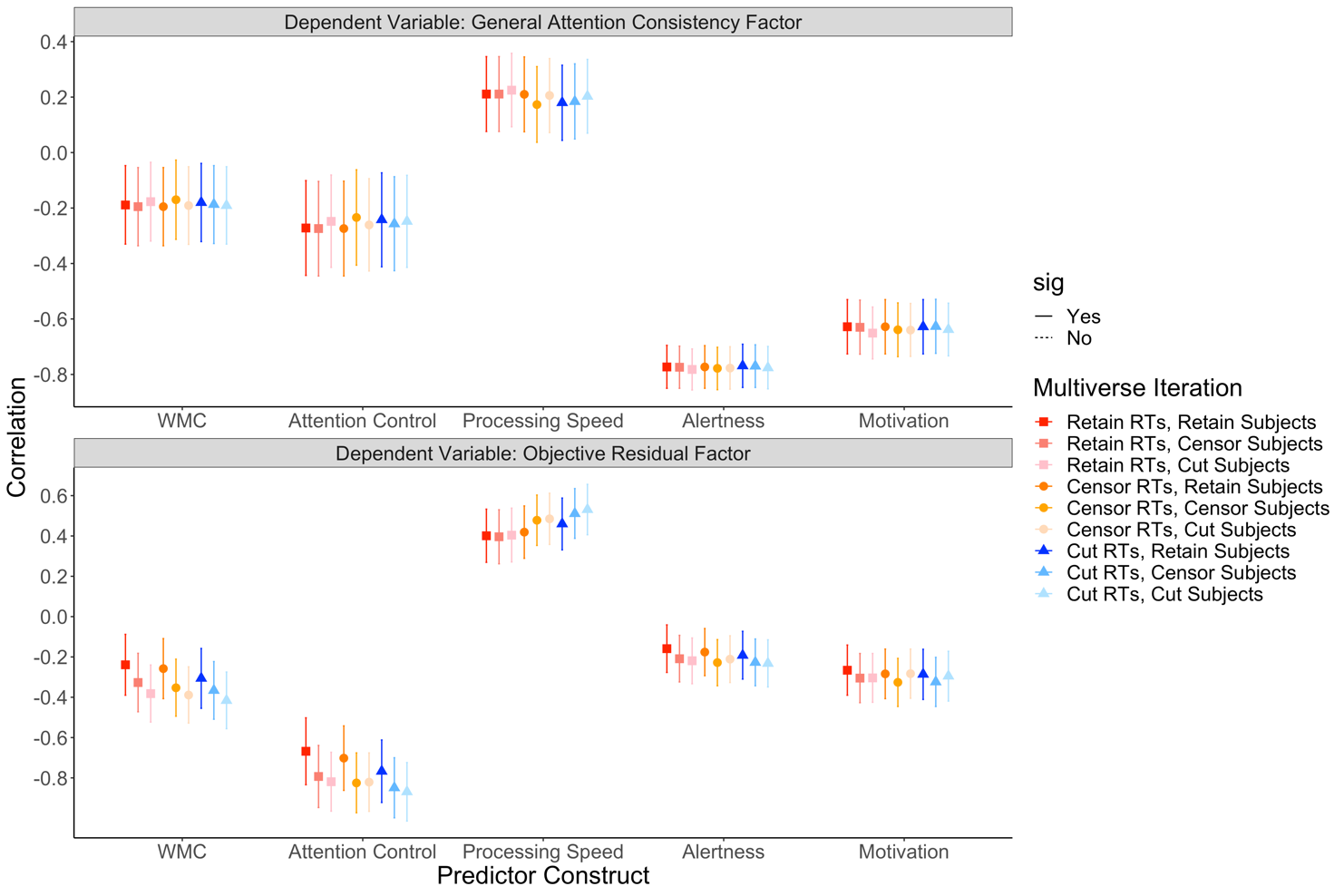  Supplemental Figure 5. Multiverse analysis results for Study 1 Objective-Residual Bifactor Model for Cognitive and Contextual Predictors. |
| --- |

| 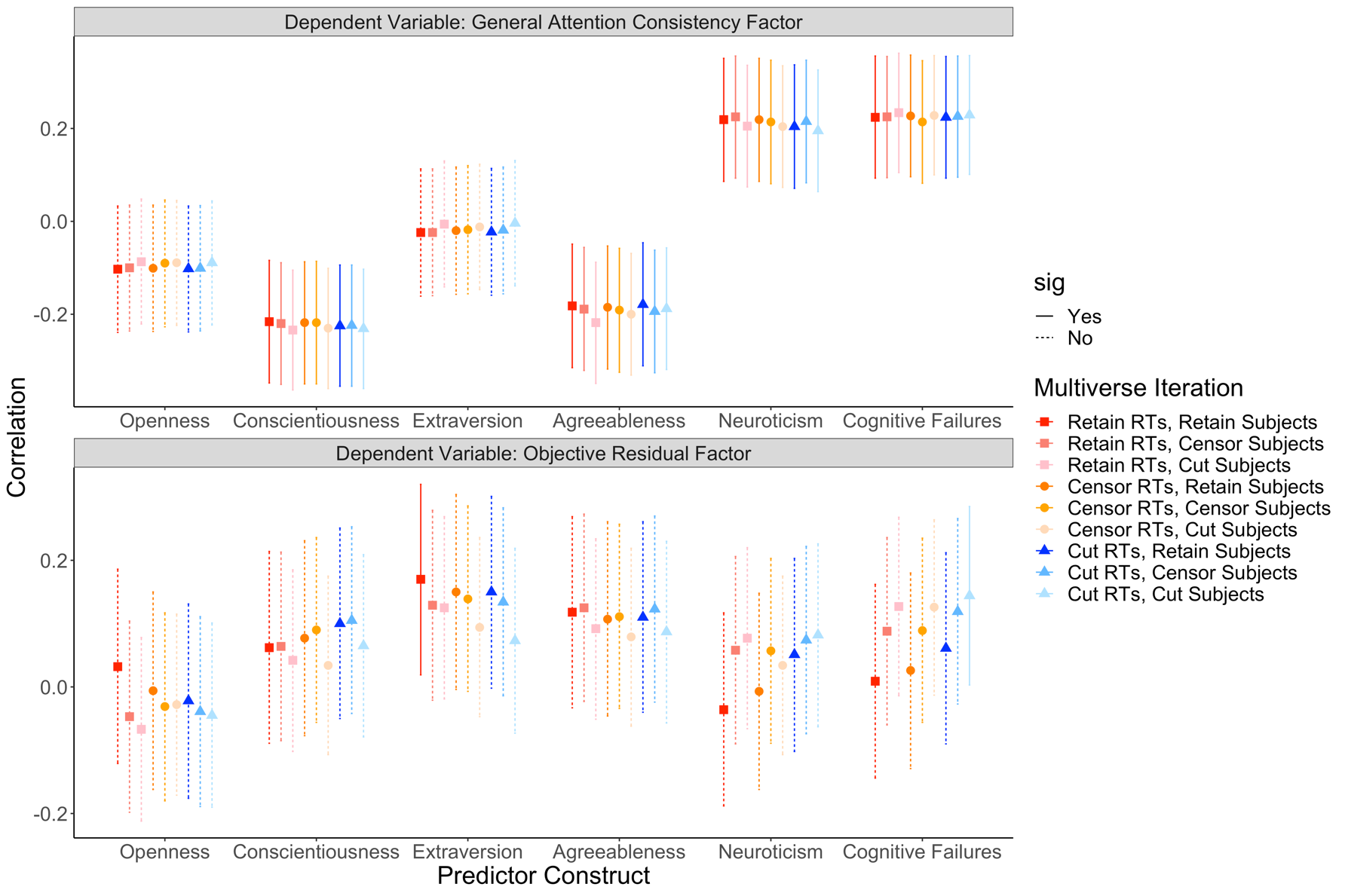  Supplemental Figure 6. Multiverse analysis results for Study 1 Objective-Residual Bifactor Model for Dispositional Predictors. |
| --- |

| 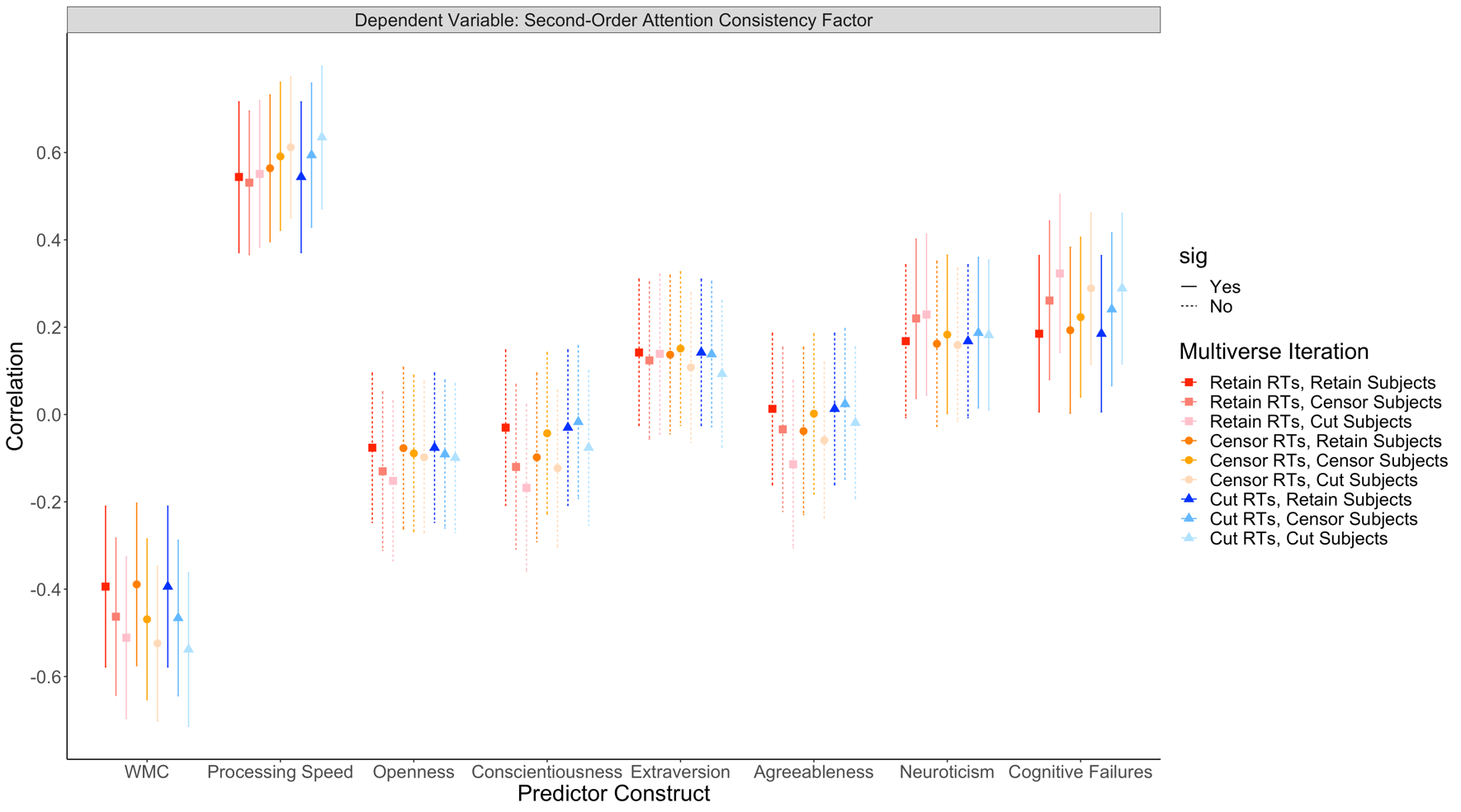  Supplemental Figure 7. Multiverse analysis results for Study 1 Hierarchical Model and Reduced set of Cognitive and Dispositional Predictors. |
| --- |

Supplemental Table 10. Descriptive Statistics for all potential Objective Attention Consistency Measures for Study 2

|  | Mean | SD | Min | Max | Skew | Kurtosis | Reliability | N | N_censored_ |
| --- | --- | --- | --- | --- | --- | --- | --- | --- | --- |
| **SART** |  |  |  |  |  |  |  |  |  |
| Tau | 139.09 | 68.41 | 0.00 | 360.23 | 1.02 | 1.31 | .98 | 520 | 9 |
| RTsd | 160.22 | 60.15 | 36.64 | 361.34 | 1.22 | 1.69 | .99 | 520 | 9 |
| RMSSD | 140.78 | 50.47 | 39.32 | 300.28 | 1.15 | 1.22 | .97 | 520 | 9 |
| RTmad | 135.16 | 49.89 | 31.13 | 308.94 | 0.90 | 1.04 | .97 | 520 | 4 |
| Bin5 | 768.84 | 175.01 | 323.71 | 1381.29 | 0.83 | 1.49 | 1.00 | 520 | 7 |
| Bin1 | 336.53 | 70.94 | 213.40 | 634.30 | 0.68 | 0.47 | 1.00 | 520 | 0 |
| Blocks | 8.29 | 12.17 | 0.00 | 39.00 | 1.46 | 0.88 | .98 | 520 | 34 |
| Omissions | 23.41 | 25.32 | 0.00 | 94.00 | 1.41 | 1.05 | .98 | 520 | 19 |
| **Number Stroop** |  |  |  |  |  |  |  |  |  |
| Tau | 91.67 | 40.17 | 14.96 | 220.92 | 1.28 | 1.52 | .92 | 468 | 8 |
| RTsd | 110.79 | 36.96 | 53.85 | 226.79 | 1.28 | 1.47 | .97 | 468 | 7 |
| RTmad | 90.16 | 24.24 | 48.93 | 170.50 | 1.07 | 1.35 | .88 | 468 | 6 |
| Bin5 | 710.58 | 119.78 | 503.40 | 1110.74 | 1.11 | 1.35 | .99 | 468 | 6 |
| **Spatial Stroop** |  |  |  |  |  |  |  |  |  |
| Tau | 160.23 | 93.08 | 0.07 | 448.73 | 1.26 | 1.42 | -- | 456 | 12 |
| RTsd | 170.68 | 86.24 | 47.71 | 427.41 | 1.37 | 1.46 | .96 | 456 | 15 |
| RTmad | 134.30 | 62.01 | 41.51 | 324.32 | 1.34 | 1.63 | .77 | 456 | 14 |
| Bin5 | 976.34 | 291.92 | 511.50 | 1857.01 | 1.33 | 1.49 | .98 | 456 | 14 |
| **Letter Flanker** |  |  |  |  |  |  |  |  |  |
| Tau | 111.78 | 62.27 | 0.00 | 324.67 | 1.14 | 1.56 | -- | 462 | 7 |
| RTsd | 122.84 | 56.61 | 40.56 | 307.44 | 1.25 | 1.45 | .94 | 462 | 9 |
| RTmad | 97.98 | 40.58 | 30.39 | 228.51 | 1.13 | 1.28 | .85 | 462 | 9 |
| Bin5 | 769.22 | 194.97 | 449.92 | 1426.82 | 1.11 | 1.22 | .98 | 462 | 5 |

Supplemental Table 10 (Continued). Descriptive Statistics for all potential Objective Attention Consistency Measures for Study 2

|  | Mean | SD | Min | Max | Skew | Kurtosis | Reliability | N | N_censored_ |
| --- | --- | --- | --- | --- | --- | --- | --- | --- | --- |
| **Arrow Flanker** |  |  |  |  |  |  |  |  |  |
| Tau | 79.17 | 35.38 | 0.00 | 192.15 | 0.94 | 0.69 | -- | 474 | 4 |
| RTsd | 89.08 | 33.07 | 33.50 | 203.48 | 1.11 | 1.12 | .89 | 474 | 1 |
| RTmad | 70.20 | 25.20 | 25.20 | 166.05 | 1.05 | 1.13 | .78 | 474 | 3 |
| Bin5 | 629.82 | 110.53 | 426.90 | 1011.21 | 0.94 | 0.74 | .97 | 474 | 1 |
| **Circle Flanker** |  |  |  |  |  |  |  |  |  |
| Tau | 113.61 | 58.74 | 0.00 | 284.81 | 1.16 | 1.32 | -- | 468 | 15 |
| RTsd | 125.77 | 52.94 | 41.42 | 282.57 | 1.33 | 1.48 | .94 | 468 | 15 |
| RTmad | 98.55 | 35.83 | 32.62 | 205.71 | 1.18 | 1.25 | .82 | 468 | 14 |
| Bin5 | 768.19 | 172.87 | 476.07 | 1303.73 | 1.22 | 1.47 | .98 | 468 | 12 |

Supplemental Table 11. Zero-order correlations of new objective indicator variables in Study 2

| Variable | 1 | 2 | 3 | 4 | 5 | 6 | 7 | 8 | 9 | 10 | 11 | 12 | 13 | 14 | 15 | 16 |
| --- | --- | --- | --- | --- | --- | --- | --- | --- | --- | --- | --- | --- | --- | --- | --- | --- |
| 1 SART_Tau | 1 |  |  |  |  |  |  |  |  |  |  |  |  |  |  |  |
| 2 SART_RTsd | 0.89 | 1 |  |  |  |  |  |  |  |  |  |  |  |  |  |  |
| 3 SART_RMSSD | 0.82 | 0.88 | 1 |  |  |  |  |  |  |  |  |  |  |  |  |  |
| 4 SART_RTmad | 0.68 | 0.84 | 0.70 | 1 |  |  |  |  |  |  |  |  |  |  |  |  |
| 5 SARTBin5 | 0.71 | 0.90 | 0.85 | 0.79 | 1 |  |  |  |  |  |  |  |  |  |  |  |
| 6 SARTBin1 | -0.27 | -0.09 | 0.07 | 0.02 | 0.34 | 1 |  |  |  |  |  |  |  |  |  |  |
| 7 SART_Blocks | 0.73 | 0.85 | 0.84 | 0.69 | 0.86 | 0.15 | 1 |  |  |  |  |  |  |  |  |  |
| 8 SART_Omissions | 0.56 | 0.52 | 0.55 | 0.28 | 0.39 | -0.24 | 0.45 | 1 |  |  |  |  |  |  |  |  |
| 9 NumStroop_Tau | 0.38 | 0.38 | 0.40 | 0.28 | 0.29 | -0.13 | 0.37 | 0.3 | 1 |  |  |  |  |  |  |  |
| 10 NumStroop_RTsd | 0.40 | 0.39 | 0.42 | 0.28 | 0.29 | -0.17 | 0.37 | 0.32 | 0.97 | 1 |  |  |  |  |  |  |
| 11 NumStroop_RTmad | 0.38 | 0.37 | 0.41 | 0.26 | 0.27 | -0.15 | 0.35 | 0.30 | 0.87 | 0.93 | 1 |  |  |  |  |  |
| 12 NumStroopBin5 | 0.37 | 0.38 | 0.41 | 0.28 | 0.31 | -0.09 | 0.37 | 0.29 | 0.92 | 0.95 | 0.87 | 1 |  |  |  |  |
| 13 SpatialStroop_Tau | 0.20 | 0.21 | 0.23 | 0.14 | 0.16 | -0.07 | 0.17 | 0.16 | 0.34 | 0.35 | 0.32 | 0.36 | 1 |  |  |  |
| 14 SpatialStroop_RTsd | 0.21 | 0.23 | 0.25 | 0.16 | 0.18 | -0.06 | 0.20 | 0.17 | 0.34 | 0.35 | 0.32 | 0.37 | 0.97 | 1 |  |  |
| 15 SpatialStroop_RTmad | 0.16 | 0.19 | 0.22 | 0.15 | 0.16 | -0.03 | 0.17 | 0.15 | 0.32 | 0.33 | 0.31 | 0.36 | 0.90 | 0.93 | 1 |  |
| 16 SpatialStroopBin5 | 0.15 | 0.20 | 0.22 | 0.14 | 0.19 | 0.02 | 0.19 | 0.13 | 0.29 | 0.31 | 0.28 | 0.36 | 0.91 | 0.95 | 0.91 | 1 |
| 17 LetterFlanker_Tau | 0.32 | 0.31 | 0.41 | 0.26 | 0.26 | -0.05 | 0.31 | 0.31 | 0.35 | 0.36 | 0.29 | 0.38 | 0.27 | 0.30 | 0.26 | 0.30 |
| 18 LetterFlanker_RTsd | 0.37 | 0.36 | 0.46 | 0.30 | 0.31 | -0.07 | 0.35 | 0.35 | 0.36 | 0.38 | 0.30 | 0.40 | 0.29 | 0.32 | 0.29 | 0.32 |
| 19 LetterFlanker_RTmad | 0.36 | 0.36 | 0.44 | 0.29 | 0.31 | -0.06 | 0.35 | 0.34 | 0.36 | 0.39 | 0.33 | 0.41 | 0.29 | 0.32 | 0.31 | 0.33 |
| 20 LetterFlankerBin5 | 0.35 | 0.35 | 0.44 | 0.29 | 0.32 | -0.02 | 0.36 | 0.33 | 0.35 | 0.38 | 0.30 | 0.43 | 0.30 | 0.33 | 0.31 | 0.35 |
| 21 ArrowFlanker_Tau | 0.26 | 0.21 | 0.25 | 0.15 | 0.15 | -0.08 | 0.22 | 0.14 | 0.35 | 0.37 | 0.35 | 0.39 | 0.32 | 0.35 | 0.34 | 0.35 |
| 22 ArrowFlanker_RTsd | 0.31 | 0.26 | 0.30 | 0.18 | 0.18 | -0.12 | 0.26 | 0.17 | 0.37 | 0.42 | 0.40 | 0.44 | 0.34 | 0.37 | 0.36 | 0.37 |
| 23 ArrowFlanker_RTmad | 0.28 | 0.23 | 0.26 | 0.15 | 0.14 | -0.15 | 0.24 | 0.16 | 0.38 | 0.42 | 0.41 | 0.45 | 0.33 | 0.35 | 0.34 | 0.36 |
| 24 ArrowFlankerBin5 | 0.28 | 0.24 | 0.28 | 0.18 | 0.19 | -0.05 | 0.26 | 0.14 | 0.33 | 0.38 | 0.37 | 0.46 | 0.33 | 0.37 | 0.37 | 0.41 |
| 25 CircleFlanker_Tau | 0.26 | 0.29 | 0.34 | 0.25 | 0.26 | 0.01 | 0.28 | 0.25 | 0.48 | 0.49 | 0.47 | 0.46 | 0.32 | 0.33 | 0.33 | 0.30 |
| 26 CircleFlanker_RTsd | 0.31 | 0.32 | 0.38 | 0.27 | 0.29 | -0.01 | 0.31 | 0.26 | 0.49 | 0.52 | 0.49 | 0.50 | 0.32 | 0.33 | 0.34 | 0.31 |
| 27 CircleFlanker_RTmad | 0.27 | 0.29 | 0.34 | 0.24 | 0.26 | 0.00 | 0.28 | 0.22 | 0.45 | 0.48 | 0.48 | 0.47 | 0.28 | 0.29 | 0.31 | 0.28 |
| 28 CircleFlankerBin5 | 0.32 | 0.35 | 0.40 | 0.29 | 0.32 | 0.02 | 0.35 | 0.26 | 0.50 | 0.53 | 0.51 | 0.55 | 0.33 | 0.35 | 0.36 | 0.35 |

Supplemental Table 11 (Continued). Zero-order correlations of new objective indicator variables in Study 2

| Variable | 17 | 18 | 19 | 20 | 21 | 22 | 23 | 24 | 25 | 26 | 27 |
| --- | --- | --- | --- | --- | --- | --- | --- | --- | --- | --- | --- |
| SART_Tau |  |  |  |  |  |  |  |  |  |  |  |
| SART_RTsd |  |  |  |  |  |  |  |  |  |  |  |
| SART_RMSSD |  |  |  |  |  |  |  |  |  |  |  |
| SART_RTmad |  |  |  |  |  |  |  |  |  |  |  |
| SARTBin5 |  |  |  |  |  |  |  |  |  |  |  |
| SARTBin1 |  |  |  |  |  |  |  |  |  |  |  |
| SART_Blocks |  |  |  |  |  |  |  |  |  |  |  |
| SART_Omissions |  |  |  |  |  |  |  |  |  |  |  |
| NumStroop_Tau |  |  |  |  |  |  |  |  |  |  |  |
| NumStroop_RTsd |  |  |  |  |  |  |  |  |  |  |  |
| NumStroop_RTmad |  |  |  |  |  |  |  |  |  |  |  |
| NumStroopBin5 |  |  |  |  |  |  |  |  |  |  |  |
| SpatialStroop_Tau |  |  |  |  |  |  |  |  |  |  |  |
| SpatialStroop_RTsd |  |  |  |  |  |  |  |  |  |  |  |
| SpatialStroop_RTmad |  |  |  |  |  |  |  |  |  |  |  |
| SpatialStroopBin5 |  |  |  |  |  |  |  |  |  |  |  |
| LetterFlanker_Tau | 1 |  |  |  |  |  |  |  |  |  |  |
| LetterFlanker_RTsd | 0.95 | 1 |  |  |  |  |  |  |  |  |  |
| LetterFlanker_RTmad | 0.88 | 0.93 | 1 |  |  |  |  |  |  |  |  |
| LetterFlankerBin5 | 0.91 | 0.97 | 0.91 | 1 |  |  |  |  |  |  |  |
| ArrowFlanker_Tau | 0.32 | 0.32 | 0.35 | 0.33 | 1 |  |  |  |  |  |  |
| ArrowFlanker_RTsd | 0.35 | 0.38 | 0.40 | 0.40 | 0.96 | 1 |  |  |  |  |  |
| ArrowFlanker_RTmad | 0.33 | 0.37 | 0.39 | 0.40 | 0.87 | 0.92 | 1 |  |  |  |  |
| ArrowFlankerBin5 | 0.36 | 0.39 | 0.41 | 0.43 | 0.92 | 0.95 | 0.88 | 1 |  |  |  |
| CircleFlanker_Tau | 0.34 | 0.37 | 0.36 | 0.35 | 0.44 | 0.45 | 0.40 | 0.43 | 1 |  |  |
| CircleFlanker_RTsd | 0.36 | 0.39 | 0.39 | 0.39 | 0.46 | 0.49 | 0.43 | 0.47 | 0.97 | 1 |  |
| CircleFlanker_RTmad | 0.33 | 0.37 | 0.38 | 0.36 | 0.43 | 0.46 | 0.42 | 0.45 | 0.88 | 0.91 | 1 |
| CircleFlankerBin5 | 0.39 | 0.43 | 0.43 | 0.44 | 0.49 | 0.52 | 0.48 | 0.53 | 0.94 | 0.97 | 0.89 |

Supplemental Table 12. Figures for Study 2 multiverse analyses

|   Supplemental Figure 8. Multiverse analysis results for Study 2 Bifactor Model. |
| --- |

| 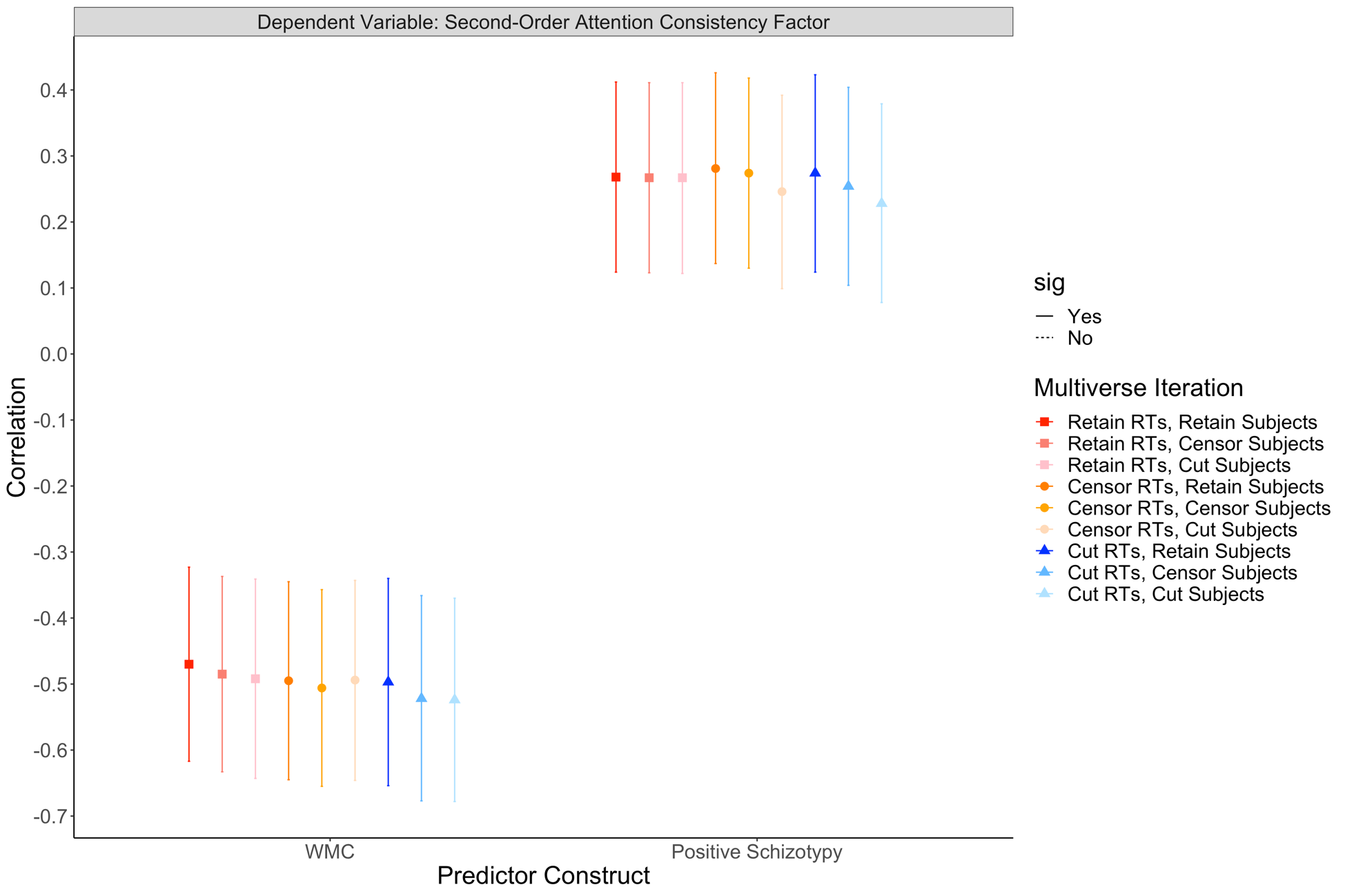  Supplemental Figure 9. Multiverse analysis results for Study 2 Hierarchical Model. |
| --- |
